# Identification of Quantitative Trait Loci and Candidate Genes for Maize Starch Granule Size through Association Mapping

**DOI:** 10.1038/s41598-018-31863-y

**Published:** 2018-09-24

**Authors:** Na Liu, Zhanhui Zhang, Yadong Xue, Shujun Meng, Yubi Huang, Weihua Li, Jihong Huang, Jihua Tang

**Affiliations:** 1 Key Laboratory of Wheat and Maize Crops Science, Collaborative Innovation Center of Henan Grain Crops, College of Agronomy, Henan Agricultural University, Zhengzhou, 450002 China; 2 College of Biological engineering, Henan University of Technology, Zhengzhou, 450001 China; 3 College of Agronomy, Sichuan Agricultural University, Wenjiang, 611130 China; 4 Hubei Collaborative Innovation Center for Grain Industry, Yangtze University, Jingzhou, 434025 China

## Abstract

Starch is an important nutrient component of maize kernels, and starch granule size largely determines kernel waxiness, viscosity, and other physiochemical and processing properties. To explore the genetic basis of maize starch granule size, 266 tropical, subtropical, and temperate inbred lines were subjected to genome-wide association analyses with an array of 56,110 random single nucleotide polymorphisms (SNPs). In the present panel, the kernel starch granule size ranged from 7–15.8 µm long and 6.8–14.3 µm wide. Fourteen significant SNPs were identified as being associated with the length of starch granules and 9 with their width. One linkage disequilibrium block flanking both sides of a significant SNP was defined as a quantitative trait locus (QTL) interval, and seven QTLs were mapped for both granule length and width. A total of 79 and 88 candidate genes associated with starch length and width, respectively, were identified as being distributed on QTL genomic regions. Among these candidate genes, six with high scores were predicted to be associated with maize starch granule size. A candidate gene association analysis identified significant SNPs within genes *GRMZM*2*G419655* and *GRMZM*2*G511067*, which could be used as functional markers in screening starch granule size for different commercial uses.

## Introduction

Starch is a widely and naturally occurring biopolymer. It is composed of D-glucose units that form two types of polymers: amylose and amylopectin. Many cereals and tuber crops produce different types of starch. For instance, maize is an important crop worldwide^1^, and starch is the main component of maize kernels, comprising ~70% of the total weight^2^. Maize starch is not only an important food source but is also one of the most industrially used resources^3^. Its uses include the preparation of soups, sauces, baked goods, dairy, confectionery, snacks, pasta, coatings, and meat-containing products^4,5^, as well as adhesives, paper, and textiles^6^.

Starch formation in cereal grains involves the synthesis of ADP-glucose (Glc) by ADP-Glc pyrophosphorylase and the incorporation of ADP-Glc into starch by ADP-Glc starch synthase^7^. Developing seeds synthesise storage compounds from imported sucrose during their maturation phase^8^. The assimilation of sucrose, which is imported through the phloem, by the endosperm may involve sucrose synthase to form ADP-Glc or UDP-Glc and fructose or, alternatively, involve invertase to form free hexose. Many studies have focused on the physicochemical properties of maize starch and their influencing factors^9–12^. Starch type^13^ and amylose content^14^ have important effects on starch properties. Other influencing factors include granular structure (shape, size, and porosity), molecular structure (organisation of growth rings and degree of crystallinity), and the presence of non-starch materials^15^.

The size of the starch granules, which depends on the plant species, is an important factors affecting starch characteristics^16^ and ultimately determines the industrial application^17^. Small starch granules can be used to replace fat in food applications because of their fat mimetic properties^18^. In food production, granule size affects the pasting properties of starch, with smaller granules showing lower peaks, troughs, and final viscosities than larger granules^19^. The starch granule size may also influence gelatinisation temperature^20^, viscosity^19^, and enzymatic susceptibility^21–23^. Additionally, it determines the grain milling yield in hard wheat^24^. In maize, the size of the starch granules varies according to chemical composition^25,26^.

Quantitative trait loci (QTLs) of starch granule sizes in *Triticeae* crops have been identified, including a major QTL related to the A:B ratio of wheat starch granules on chromosome 4S^27^ and a QTL on barley chromosome 2^28^. Recently, genome-wide association studies (GWASs) have been proven to be useful tools for the identification of candidate loci associated with traits in animal and plant species^29^. For example, an analysis of maize oil biosynthesis identified 74 loci significantly associated with kernel oil concentration and fatty acid composition in a GWAS using 1 million single nucleotide polymorphisms (SNPs) characterised in 368 inbred maize lines^30^. Furthermore, a GWAS and QTL mapping were found to be complementary, overcoming each other’s limitations, in *Arabidopsis*^31^.

Compared with starches having a bimodal size distribution^17,32–34^, few studies have investigated the unimodal starches, particularly that of maize^35^. Although the sizes of maize starch granules are highly linked to the end-use quality of the products, many studies on maize starch have focused on its processing and nutritional properties^35^, with little attention paid to the study of granule size^34^. Here, we used a set of associated populations to identify significant SNP markers for starch granule size with the aim of predicting associated candidate genes.

## Results

### Phenotypic analysis of maize starch granule size

**Figure 1:**
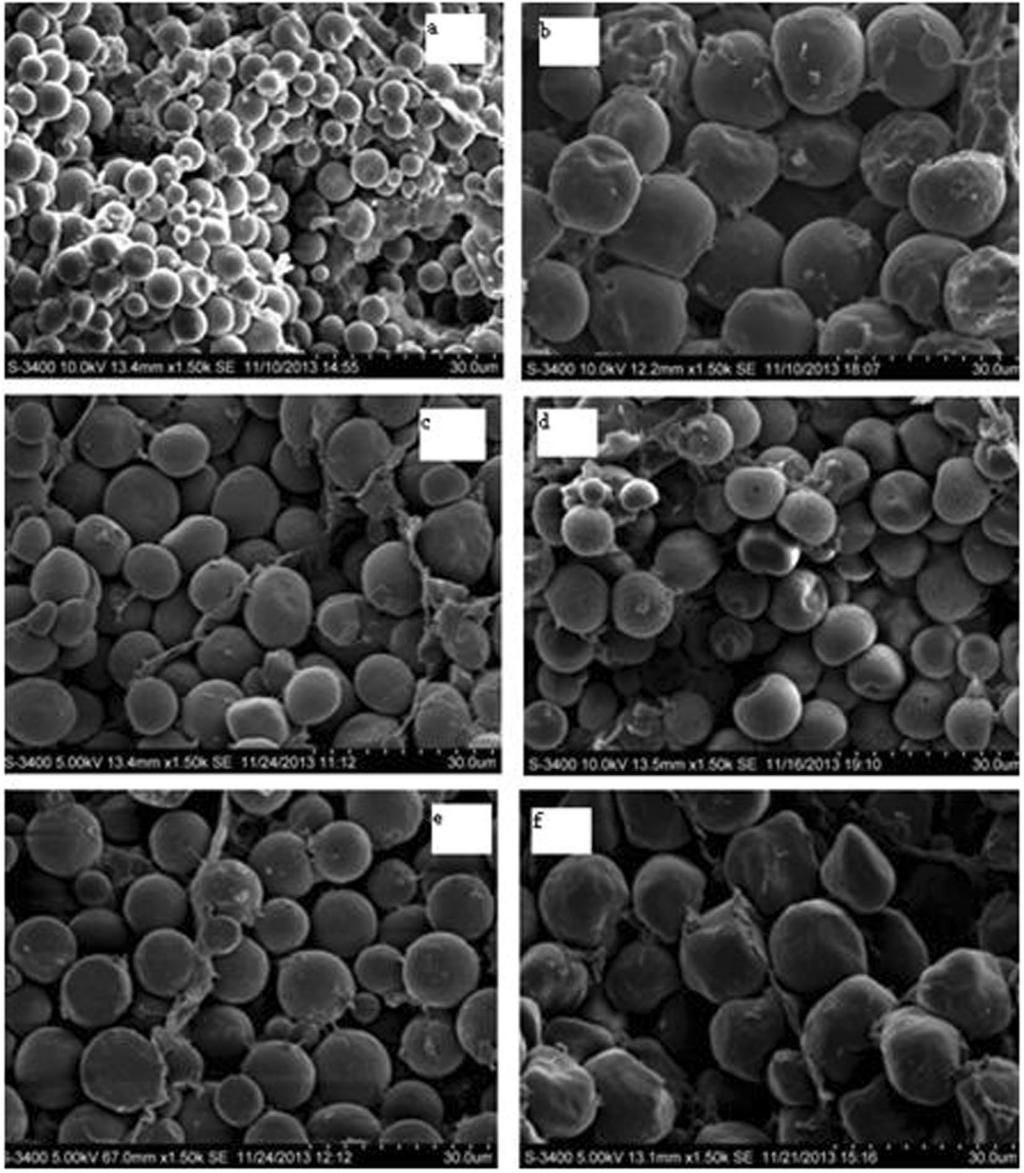
Scanning electron micrographs of starch granules in kernels of inbred maize lines. (**a**) CIMBL30; (**b**) CML470; (**c**) GEMS52; (**d**) 7884-4Ht; (**e**) 526018; (**f**) GEMS65.

**Figure 2:**
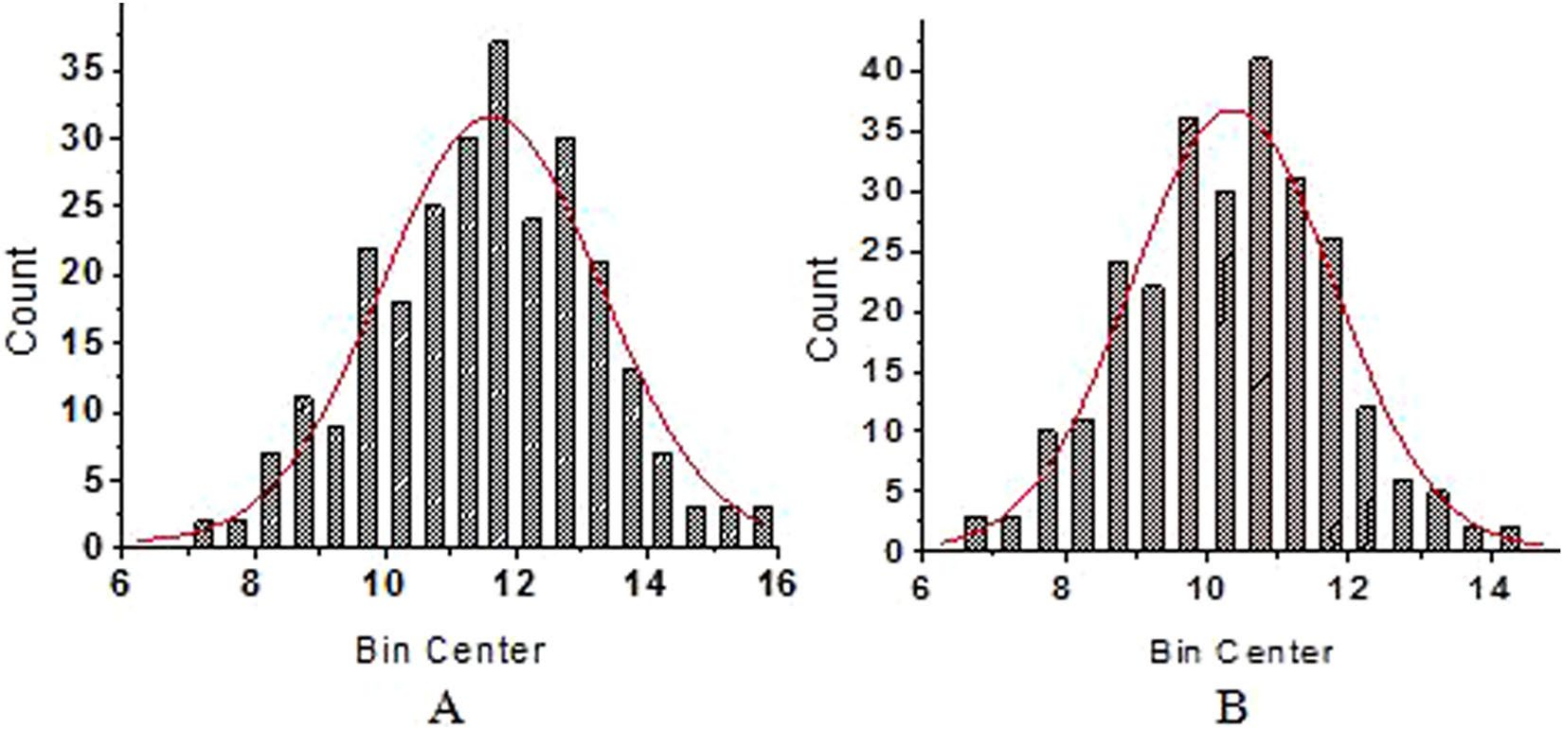
Frequency map of starch granule sizes. (**a**) Frequency of granule lengths; (**b**) Frequency of granule widths.

A total of 266 maize lines were used for association mapping. Although the starch granules of these inbred maize lines varied largely in size, more than 75% of the granules were 10–13.5 µm long and 9.7–11.8 µm wide (Table S1). The inbred line CIMBL30 had the smallest granule size (7 µm long × 6.8 µm wide; Fig. 1a), while the inbred line CML470 had the largest granule size (15.8 µm long × 14.3 µm wide; Fig. 1b). The starch granules of most inbred lines had a smooth surface (Fig. 1a–c), although some were rough or porous/cracked (Fig. 1d). The shapes varied, including rounded, spherical (Fig. 1e), or irregular (Fig. 1f). Thus, the sizes and shapes of the starch granules varied among different inbred lines (Fig. 2, Table 1), which may affect starch processing characteristics and seed unit weight.

**Table 1:** ANOVA of starch granule length and width in inbred maize lines.

Trait		SS	DF	MF	F	P
Starch granule length	Between Groups	2211.491	266	8.314	122.387	0
	Within Group	36.071	531	0.068		
	Total	2247.562	797			
Starch granule width	Between Groups	1554.199	266	5.843	104.063	0
	Within Group	29.814	531	0.056		
	Total	2247.562	797			

### Evaluation of starch pasting viscosity characteristics

The rapid visco analyser (Newport Scientific, Australia) profile revealed the paste viscosity characteristics of maize starch (Table S2). Seven parameters showed that large starch granules (such as in ‘CIMBL12’ and ‘Zheng58’) have smaller final viscosity levels than smaller starch granules.

### Association analysis

**Figure 3:**
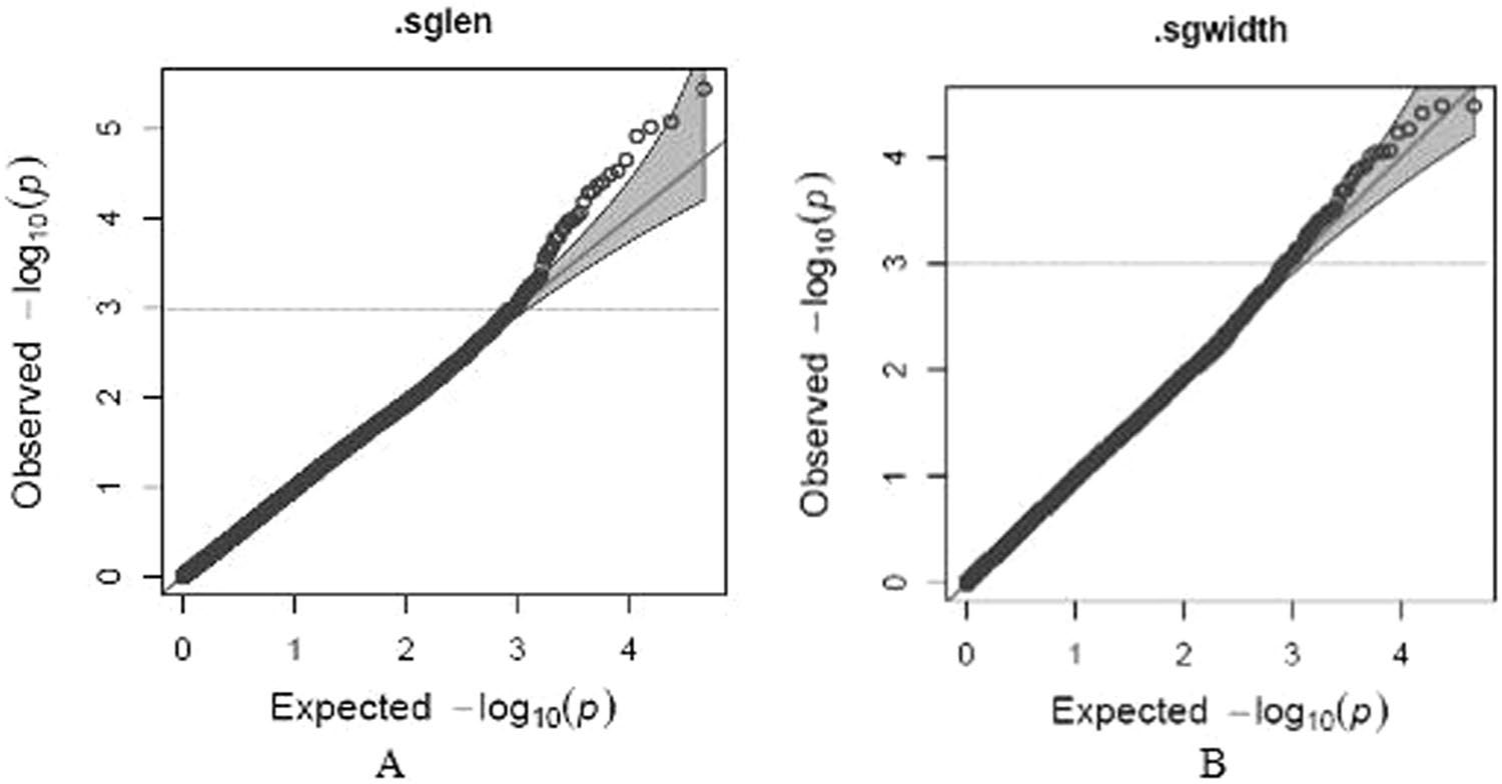
Quantile–quantile plot of associations with starch granule size. The *p*-values are shown on a −a rapid visco analyser accordinglog10 scale, and the dashed line indicates a Bonferroni-corrected threshold of 0.1/N. (**a**) Starch granule length; (**b**) Starch granule width.

**Figure 4:**
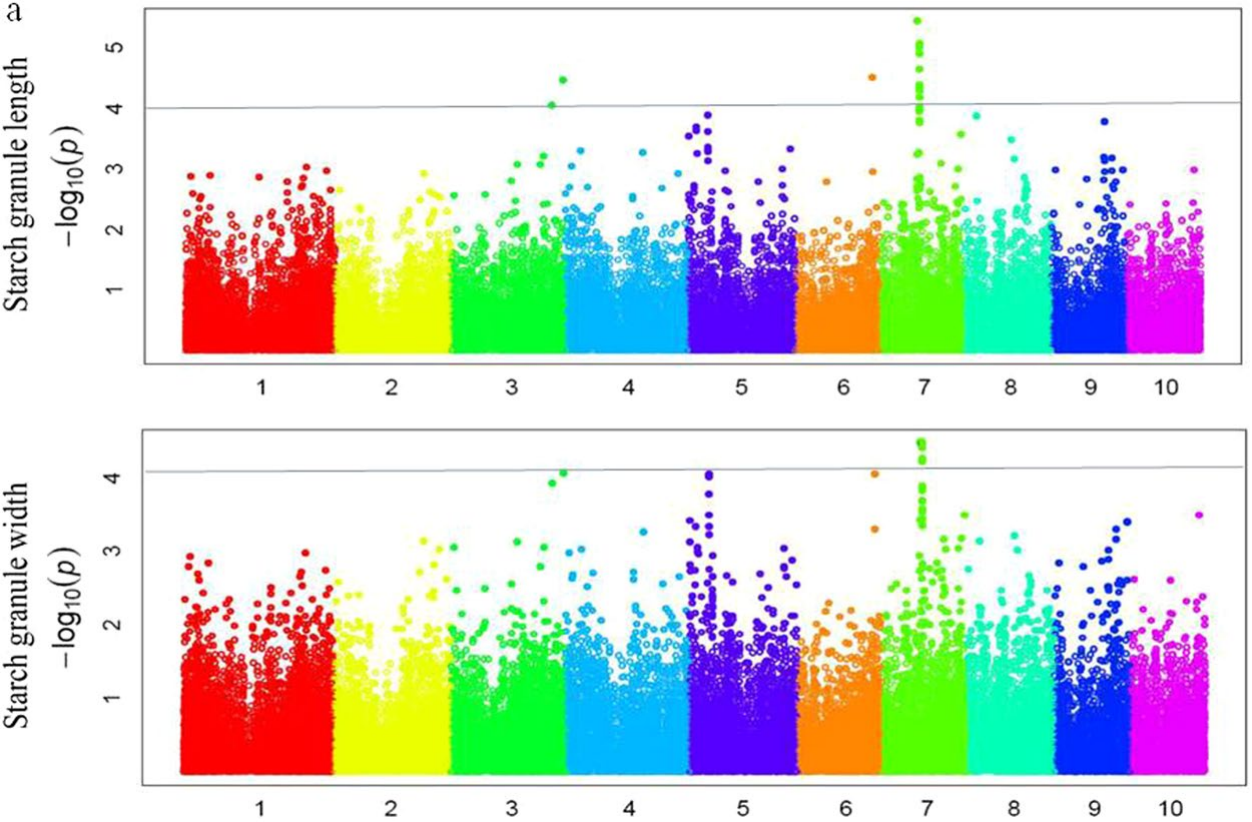
Manhattan plot of starch granule size.

The average data from different replicates of each inbred line were used for association analysis (Figs 3 and 4). For starch granule size, 14 significant SNPs were identified (p < 2.25 × 10^−4^; Fig. 4, Table 2), with 1, 2, and 11 SNPs distributed on chromosomes 6, 3, and 7, respectively. Seven QTLs, distributed over 79 candidate genes, were identified for starch granule length (Table 2).

**Table 2:** Quantitative trait loci, single nucleotide polymorphisms, and candidate genes for maize starch granule size.

QTL	Position of QTL	SNP	Position of SNP	Candidate gene
*qSGL7-1*	75753279–75953279	SNP30343	75853279	GRMZM2G180104, GRMZM5G866141, GRMZM2G544148, GRMZM2G020156, GRMZM2G172345, GRMZM2G172307
*qSGL7-2*	80321800–80573170	SNP30418 SNP30419 SNP30423 SNP30425	80421800 80421839 80493311 80573170	GRMZM2G324991, AC206845.3_FG004
*qSGL7-3*	80645735–80882295	SNP43662 SNP30432 SNP30430	80745735 80782295 80748880	AC209906.3_FG001, GRMZM2G061010, GRMZM2G061014, GRMZM2G003165, GRMZM2G486223, GRMZM2G306371, GRMZM2G486201
*qSGL7-4*	80935113–81135283	SNP30436 SNP30438 SNP30437	81035068 81035283 81035113	GRMZM2G419655, GRMZM2G419660, AC196428.3_FG003, GRMZM2G177900, GRMZM2G542753
*qSGL3-1*	202581272–202781272	SNP1148	202681272	GRMZM2G511067, GRMZM2G048290, GRMZM2G347659, GRMZM2G347675, GRMZM2G054340, GRMZM2G054351, GRMZM2G054221, GRMZM5G858429, GRMZM2G054115, GRMZM5G857098, GRMZM2G591200, GRMZM2G181135, GRMZM2G702146, GRMZM2G017308, GRMZM2G017310
*qSGL3-2*	225599972–225799972	SNP17473	225699972	GRMZM2G12277, GRMZM2G033304, AC200626.3_FG008, GRMZM2G303999,GRMZM2G168049, GRMZM5G810209, GRMZM2G702310, GRMZM2G003254, GRMZM2G076245, GRMZM2G070775, GRMZM2G529436, GRMZM5G837511, GRMZM2G369956, AC209364.3_FG009, GRMZM2G369931, GRMZM2G070405, AC209364.3_FG007, GRMZM2G524240, GRMZM2G369839
*qSGL6*	154943436–155143436	SNP28873	155043436	GRMZM2G522194, GRMZM2G067073, GRMZM2G522185, GRMZM2G501825, GRMZM2G037343, GRMZM5G897015, GRMZM2G037286, GRMZM2G501821, GRMZM2G037111, GRMZM2G129031, GRMZM2G167673, GRMZM2G429714, GRMZM2G129064, GRMZM5G851528, GRMZM2G559994, GRMZM2G429720, GRMZM2G129166, AC206988.3_FG005, GRMZM2G129230, GRMZM5G834657, GRMZM2G310552, GRMZM5G896682, GRMZM2G010764, GRMZM2G010357, GRMZM2G488711
*qSGW7-1*	75753279–75953279	SNP30343	75853279	same as qSGL7-1
*qSGW7-2*	80321800–80673170	SNP30418 SNP30423 SNP30425	80421800 80493311 80573170	same as qSGL7-2
*qSGW7-3*	80935068–81135068	SNP30436	81035068	same as qSGL7-3
*qSGW3*	225599972–225799972	SNP17473	225699972	same as qSGL3-2
*qSGW6*	154943436–155143436	SNP28873	155043436	same as qSGL6
*qSGW5-1*	39834864–40034864	SNP22884	39934864	GRMZM2G012167, GRMZM5G861448, GRMZM2G012209, AC206260.3_FG004, GRMZM2G012926, GRMZM2G012933, GRMZM2G012958, GRMZM2G013196, AC203800.3_FG005, GRMZM2G134623, GRMZM2G563606, GRMZM2G134629, GRMZM2G134597, GRMZM2G000005, AC203800.3_FG001, GRMZM2G524940, GRMZM2G435497, GRMZM2G000005, GRMZM2G492258, AC203800.3_FG006, GRMZM2G552085, GRMZM2G000007, GRMZM2G482454, GRMZM2G000009, GRMZM2G482456, GRMZM2G482457
*qSGW5-2*	39979303–40179301	SNP22889	40079301	GRMZM2G320731, GRMZM2G494899, GRMZM2G014180

Note: SGL, starch granule length; SGW, starch granule width.

**Figure 5:**
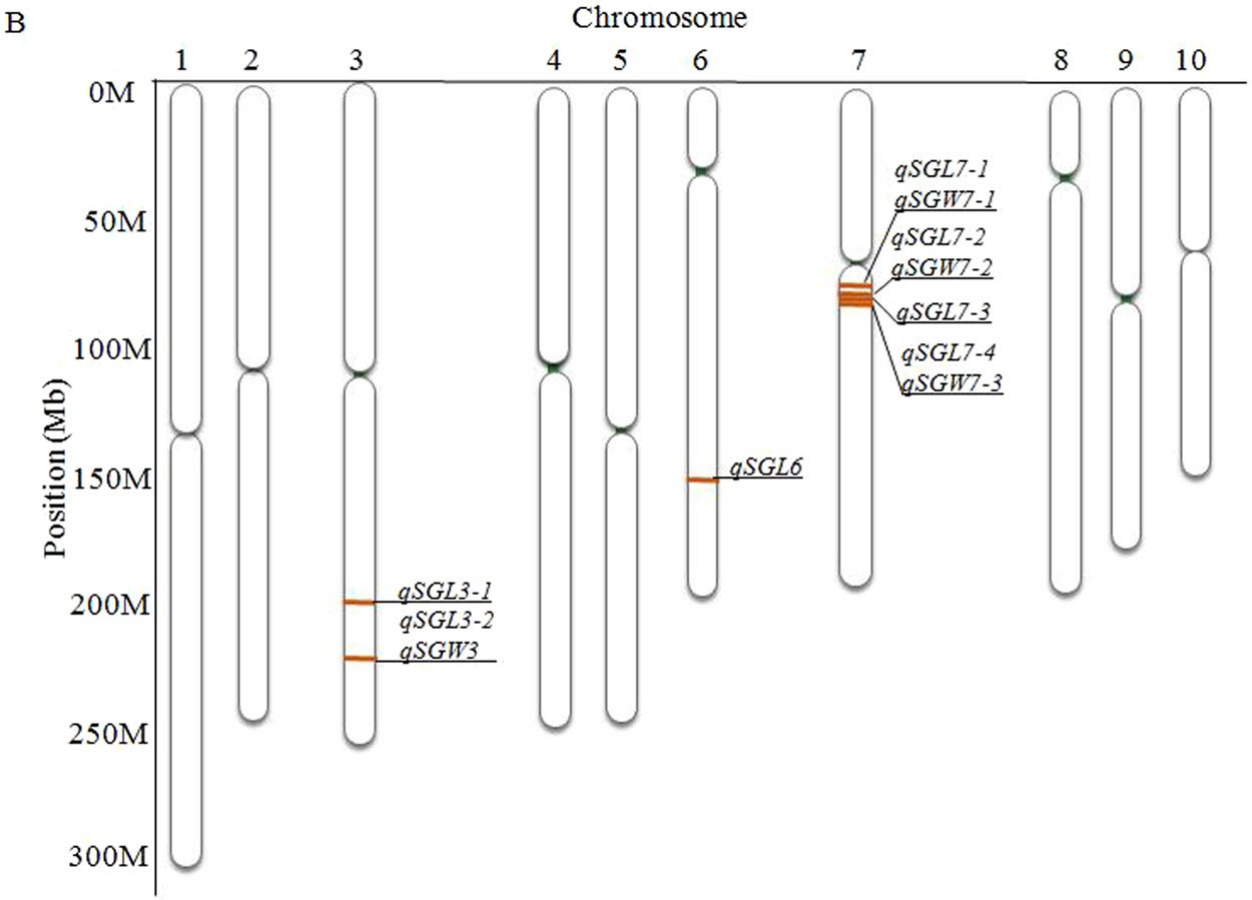
The chromosomal locations of the identified QTL for maize kernel starch granule.

Nine significant SNPs were identified to be associated with starch granule width, as well as seven QTLs and 88 candidate genes (Fig. 5, Table 2). Seven SNPs were identified for both starch granule length and width: one (*PZE-103182712*) on chromosome 3, one (*PZE-106103012*) on chromosome 6, and five (*PZE-107043911*, *PZE-107044857*, *PZE-107044898*, *PZE-107044943*, and *PZE-107045024*) on chromosome 7.

### Gene ontology analysis

The QTLs analysis led to the indentification of 108 candidated genes that were either associated with granele length or width. Among these, six genes with higher scores were located close to associated SNPs (Table 3). *GRMZM2G180104* was located between 75,849,776–75,850,502 bp on chromosome 7 and 3,503 bp upstream of significant SNP30343, while *GRMZM2G419655* and *GRMZM2G419660*, also on chromosome 7, were identified as being associated with starch granule size.

**Table 3:** Candidate genes for maize starch granule size.

Score	Gene ID	Chr.	SNP pos(bp)	Distance to SNP (bp)
5.44	*GRMZM2G180104*	7	75853279	3503
5.07	*GRMZM2G419655*	7	81035068	3127
5.07	*GRMZM2G419660*	7	81035068	3097
4.52	*GRMZM2G167673*	6	155043436	8547
4.47	*GRMZM6G663759*	3	225699972	7418
4.06	*GRMZM2G511067*	3	202681272	2118

The Blast2Go program was used to predict the functions of these candidate genes (Tables 3 and S3, S4). The candidate gene on chromosome 6, *GRMZM2G167673*, was predicted to be involved in gibberellin synthesis and electron transport as a p450 cytochrome. *GRMZM2G419655* and *GRMZM2G419660* on chromosome 7 were predicted to encode phytosulfokine receptor precursors. *GRMZM2G511067* and *GRMZM6G663759* on chromosome 3 were predicted to encode a zinc finger CCCH domain-containing protein that binds metal ions and may repress the inhibitor of the phytosulfokine receptor protein kinase.

The results of the GWAS analysis revealed that maize kernel starch granule size is a typical quantitative trait determined by multiple genes.

### Association between candidate genes and maize starch granule size

**Figure 6:**
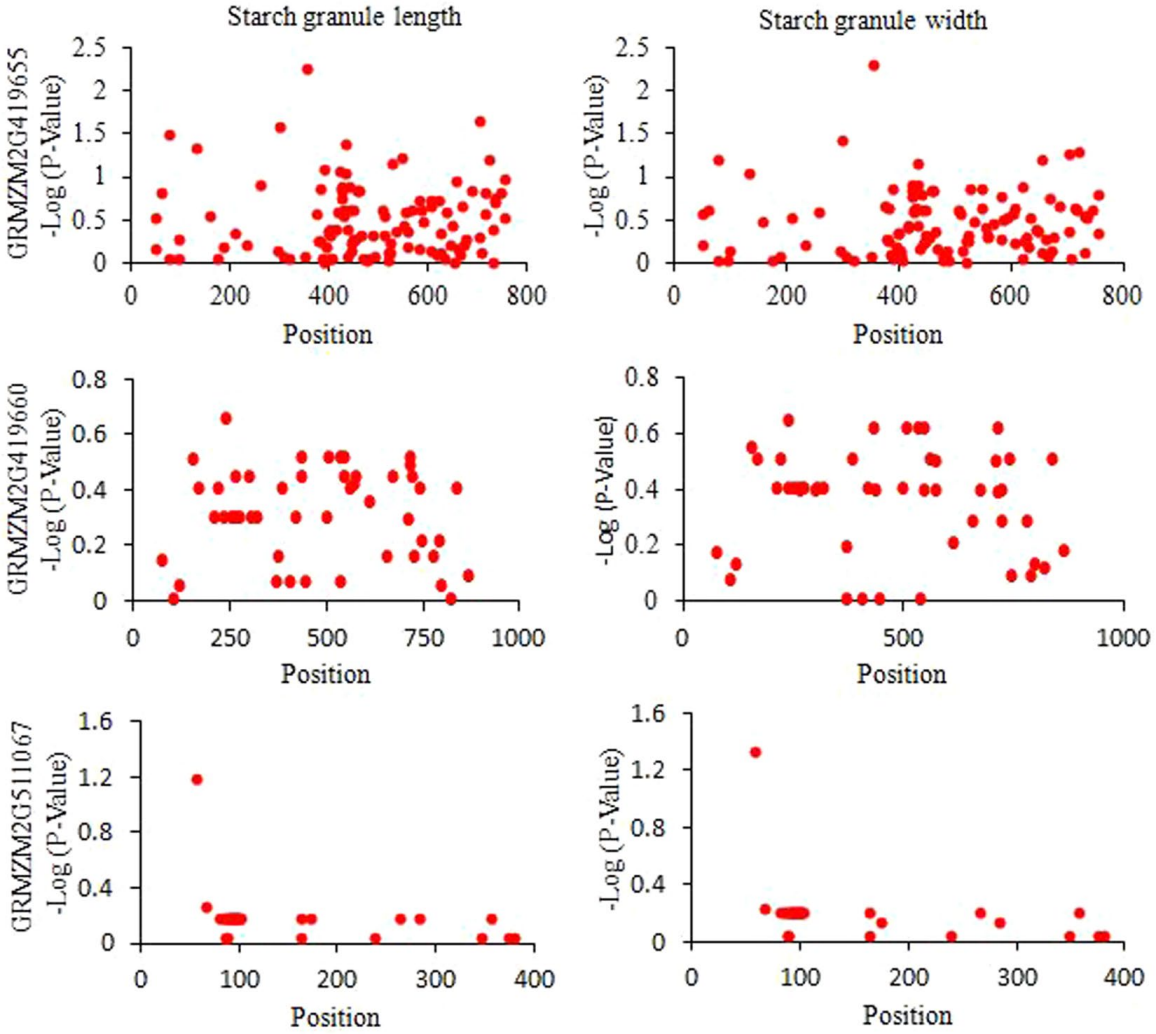
Candidate gene association analysis for three candidate genes and maize granule size.

An association analysis beween three candidate genes and maize starch granule size revealed that the SNP at 352 bp of the *GRMZM2G419655* genomic sequence and the SNP at 58 bp of the *GRMZM2G511067* genomic sequence were significantly associated with maize starch granule length and width (Fig. 6). No significant SNP was identified after an association analysis between *GRMZM2G419660* and maize starch granule size. All of the sequences of the three candidate genes are shown in Supplementary file S5.

### Expression levels of candidate genes in maize lines with different size starch granules

**Figure 7:**
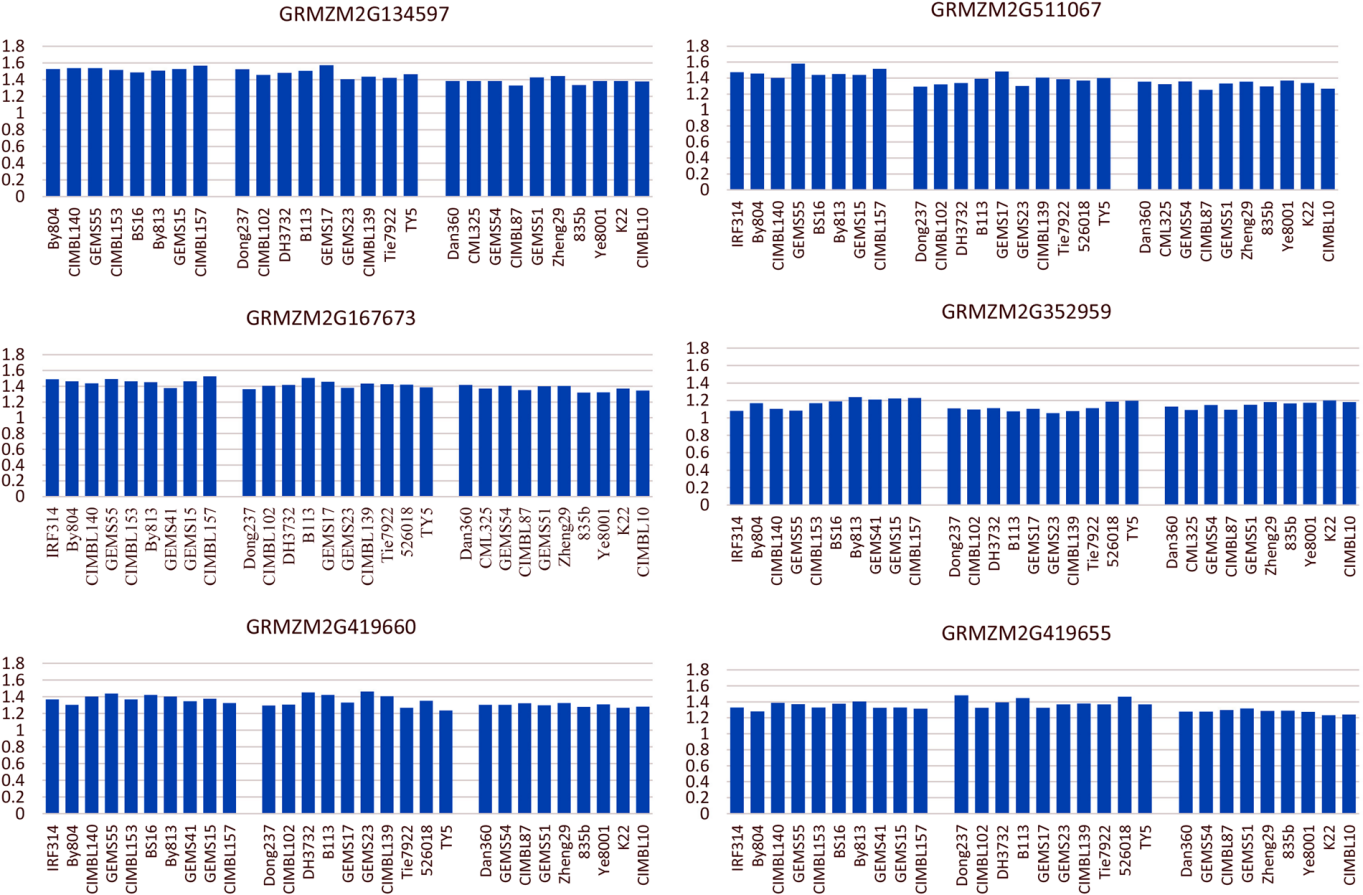
Different expression level of candidate genes.

To verify the predicted candidate genes, 10 of them were chosen to study the differences in their expression levels within different starch granule size groups using reverse transcription and fluorescence quantitative PCR. The result are shown in Table 4 and Fig. 7. Six in the 10 selected genes, including *GRMZM2G134597*, *GRMZM2G167673*, *GRMZM2G419660*, *GRMZM2G511067*, *GRMZM2G352959* and *GRMZM2G419655*, showed significant difference at 20 d after pollination.

**Table 4:** Expression levels of different candidate genes for maize starch granule size.

Gene ID	Mean _SSG_	Mean _MSG_	Mean _LSG_	t-value SSG/MSG	t-value SSG/LSG	t-value MSG/LSG
*GRMZM2G134597*	1.54 ± 0.02	1.47 ± 0.05	1.38 ± 0.05	1.36	5.54**	−0.42
*GRMZM2G167673*	1.46 ± 0.04	1.42 ± 0.04	1.37 ± 0.04	1.55	5.26**	2.43*
*GRMZM2G369956*	1.20 ± 0.03	1.19 ± 0.02	1.18 ± 0.02	1.48	2.92*	1.31
*GRMZM2G419660*	1.37 ± 0.05	1.35 ± 0.08	1.29 ± 0.02	1.43	7.96**	2.30*
*GRMZM2G511067*	1.46 ± 0.05	1.37 ± 0.05	1.32 ± 0.05	3.92**	4.34**	1.71
*GRMZM2G003165*	1.48 ± 0.06	1.51 ± 0.05	1.51 ± 0.07	−0.69	−0.37	0.45
*GRMZM2G014180*	1.15 ± 0.02	1.14 ± 0.02	1.14 ± 0.02	1.20	0.79	−0.52
*GRMZM2G352959*	1.17 ± 0.07	1.11 ± 0.05	1.14 ± 0.04	2.89**	1.30	−2.74*
*GRMZM2G419655*	1.35 ± 0.05	1.40 ± 0.06	1.28 ± 0.03	−2.52*	6.75**	5.55**
*GRMZM2G542753*	1.23 ± 0.07	1.23 ± 0.05	1.21 ± 0.05	0.17	1.26	1.20

Note: SSG, gene expression of the small starch granule inbred line group; MSG, gene expression of the medium starch granule inbred line group; LSG, gene expression of the large starch granule inbred line group;

t-value SSG/MSG, t-value of the t-test between SSG and MSG; t-value SSG/LSG, t-value of the t-test between SSG and LSG; t-value MSG/LSG, t-value of t-test between MSG and LSG;

*, 0.05 significance level; **, 0.01 significance level.

## Discussion

### Analysis of the maize starch granule size

Starch is the major storage carbohydrate in cereal seeds, and the size of the starch granules is strongly associated with its end use. However, it is difficult to accurately determine the size of starch granules. To date, two techniques have been developed to analyse granule sizes: laser light scattering (LDS)^36^ and digital image analysis (IA)^37^. LDS for particle size analysis is simple to perform; however, the starch granule’s oblate shape can cause the laser to diffract from the flat surface or narrow edge, or at obtuse angles to these surfaces, leading to system errors. Comparing LDS with IA, Wilson *et al*.^38^ reported that LDS underestimated A-type granule’ diameters by ~40% and B-type granule’ diameters by ~50% in wheat. Edwards *et al*.^24^ revealed that LDS measurements underestimated C-, B-, and A-type granules by maximum averages of 0.83, 3, and 23 mm, respectively. Additionally, LDS requires a prior starch extraction, which may cause artefacts to develop during extraction or precipitation^39^. Therefore, LDS is more suitable for the analysis of totally spherical granules. Thus, LDS has often been used in the study of wheat starch but rarely in the study of maize starch.

Starch granule size has been measured by other direct methods, such as light microscopy and scanning electron microscopy (SEM). Chen *et al*.^40^ analysed the size of starch granules in *Brachypodium distachyon* by SEM, while Zhang *et al*.^26^ used a light microscope with the Zeiss software AxioVision to observe the starch granule size in potato. Compared with LDS, IA coupled with light microscopy or SEM is more direct and more readily distinguishes among individual granules, agglomerated granules, and non-starch particles. It can also simultaneously record the surface features of individual particles. Considering the shape diversity of maize starch granules, IA with SEM was chosen as a more accurate method of obtaining direct data in this study.

In cereal, the size of the starch granules is an important property affecting the appropriate industrial use^17^. Variations in starch granule size mainly exist among inbred lines of maize, which allows for the selection of different commercial hybrids having the required granule size, resulting in improved industrial use.

### Association analysis of maize starch granule size

Association analyses are effective tools in finding candidate genes and putative functional markers for simple and complex plant traits^41,42^. In the current study, 14 and 9 SNPs were significantly associated with maize starch granule length and width, respectively. Among them, seven significant SNPs and five QTLs were shared between starch granule length and width (Table 2).

In a candidate gene analysis for starch granule size, *GRMZM2G167673* was predicted to encode cytochrome P450 (CYP) 714D (CYP714D). In plants, CYP is involved in several cellular processes. A homologous gene in rice encodes the CYP protein, which regulates the embryo to endosperm ratio and increases the proportion of kernel endosperm^43^. More recently, the insertion of a 247-bp transposable element into the 3′-untranslated region of *ZmGIANT EMBRYO 2* (*ZmGE2*, encodes a CYP protein) was associated with an increased embryo to endosperm ratio^44^. In the present study, the gene ontology analysis revealed that GRMZM2G167673 has monooxygenase activity. Subfamily members of CYP, including CYP78A in maize also have monooxygenase activities, with CYP78A5, CYP78A7, and CYP78A9 regulating organ size by generating mobile growth signals that stimulate cell proliferation^45,46^. Thus, GRMZM2G167673 may regulate endosperm growth, determining the size of the starch granules.

Another candidate gene associated with starch granule size, *GRMZM2G419660*, encodes a protein with serine/threonine kinase activity. Serine/threonine kinases are a subfamily of calcium-dependent protein kinases (CDPKs) in plants^47^; moreover, overexpressing and silencing the CDPK gene *OsCPK31* indicated that it regulates grain filling and early maturation in the Taipei 309 rice cultivar. A SEM examination showed that the starch granules increased in size when *OsCPK31* was overexpressed compared with in non-transformed controls^48^. Thus, GRMZM2G419660 in maize may also be an essential factor for the phosphorylation of sucrose synthase, which is a major enzyme involved in the starch biosynthetic pathway, similar to protein kinases in rice.

The size of the starch granules is an important factor influencing the industrial applications of starch. In the present study, inbred maize lines with different starch granules sizes were evaluated. Maize lines with different sized starch granules can be used in different industries. Moreover, SNPs or candidate genes identified in this study could be used as molecular markers to accelerate the breeding and production of plants with starch granules appropriate for different commercial purposes.

## Methods

### Plant materials

The investigation was based on a set of 266 inbred maize lines, containing a wide range of temperate, subtropical, and tropical germplasm^49,50^. Because some tropical germplasm cannot mature in temperate zones, affecting the starch content and granule size, all of the inbred lines were cultivated in Sanya (Hainan Province, PR China; E 18°37′, N 18°09′) during the winters of 2012 and 2013. The field experiment followed a randomised complete block design with two replications. Plots were 4 m × 0.67 m and comprised 16 plants at a density of 65,250 plants per hectare. During the growing seasons, plants were irrigated and underwent common field management practices to avoid any stress.

### Evaluation of starch granule size and starch paste viscosity characteristics

All of the inbred lines were self-pollinated by hand in the field, harvested when physiologically mature, and dried under natural conditions; those ears that showed abnormal development were subsequently discarded. Kernels in the middle part of each ear were then hand-threshed for starch granule size evaluation. Ten representative matured and dried kernels were selected (five kernels from each ear) and affixed to aluminium specimen stubs using double-sided adhesive tape. The samples were then sprayed with gold powder and screened using SEM (Hitachi S-3400, Tokyo, Japan) at the Centre of Biotechnology, Henan Agricultural University, Zhengzhou, China. The sizes of 20 randomly selected maize starch granules were evaluated for length and width. Data were analysed using the analysis of variance method with SPSS software (IBM Corp., Armonk, NY, USA). A frequency map was constructed by Origin 8.0 software (OriginLab Corporation, Northampton, MA, USA). Sample means were used as phenotypic data for an association mapping analysis.

Four maize lines were selected to extract starch, and the pasting properties of the starch were measured using a rapid visco analyser according to Hao *et al*.^51^.

### Genotyping and association analysis

All statistical analyses were performed using the R statistical environment (www.r-project.org). Frequency plots were also constructed by the plot function in R. Averaged data for each inbred line were used in the association analysis.

Selected inbred lines were genotyped using two genotyping platforms (RNA-sequencing and SNP array) containing 56,110 SNPs according to the method described by Yang *et al*.^52^. SNP data are available from http://www.maizego.org/Resources.html. SNPs with more than 12% missing data and a minor allele frequency <5% were excluded, resulting in 47,237 SNPs for further analyses. The linkage disequilibrium (LD) between SNPs on each chromosome was estimated with *r*^*2*^ using TASSEL 5.0^53^. A mixed linear model with the obtained SNPs, principal components, kinships, and the mean starch granule sizes was used for the GWAS. The relative distribution of −log_10_ *p*-values was observed for each SNP association and compared individually with the expected distribution using a quantile–quantile plot. The adjusted *p*-value threshold of significance in each trait was corrected. SNP loci in significant LD regions were identified by revealing significant contributions to the phenotypic variations of the agronomic traits with the highest *r*^*2*^ values (magnitude of marker–trait association) and lowest adjusted *p*-values (threshold *p* < 1 × 10^−4^).

The overall LD decay across the genome of this panel was 100 kb^54^, thus a 100-kb region flanking the left and right sides of a SNP was defined as a QTL. If several SNPs were located closely within one LD block, the middle coordinate was chosen.

### Analysis of candidate genes

The available maize genome sequence (B73) was used as the reference genome for candidate gene identification. SNP probe sequences of ~120 bp (Illumina Inc., San Diego, CA, USA) were used as queries in a BLAST algorithm-based search against the reference genome sequence in MaizeGDB (http://www.maizegdb.org/gbrowse). Based on the LD decay, a 200-kb window for the significant SNPs (100-kb upstream and downstream of the lead SNP) was selected to identify candidate genes. Genes within the region were identified according to the position of the closest flanking significant SNP (*p* < 1 × 10^−4^). The Blast2Go program was used to predict the functions of corresponding genes (http://www.geneontology.org/).

### Sequencing and candidate gene association analysis

Three candidate genes, *GRMZM2G419655*, *GRMZM2G419660*, and *GRMZM2G511067*, were selected for sequencing based on the GWAS. DNA was extracted from seedlings of 26 maize lines with the largest starch granule sizes and 21 maize lines with the smallest starch granule sizes^55^. PCR reaction mixes (20 µl) contained 1 µl of NEB (New England BioLabs Inc., Ipswich, MA, USA) Taq DNA Polymerase, 4 µl of 5× NEB PCR Buffer, 0.5 µl of dNTP mixture, 0.5 µl each of the two primers, and 1 µl of template DNA. The PCR reaction was carried out in a Bio-Rad Thermal cycler (Bio-Rad Laboratories, Inc., Hercules, CA, USA)with an initial denaturation at 94 °C for 3 min followed by 34 cycles of denaturation for 10 s at 94 °C, annealing for 1 min at 64 °C and extension for 1 min at 68 °C, with a final extension for 10 min at 68 °C. SNPs within the three candidate gene sequences were selected for the association analysis. Primers for amplification of the three genes are listed in Table 5.

**Table 5:** PCR primers for candidate maize genes.

Candidate gene		PCR Primer
*GRMZM2G511067*	Froward	5′-AAACTTGTTCGATTTGGT-3′
	Reverse	5′-CACATATAAAGGATCATAA-3′
*GRMZM2G419655*	Froward	5′-AATCAGTTCACTGGCAACC-3′
	Reverse	5′-TCGCTTGCTCGAGCCATTAT-3′
*GRMZM2G419660*	Froward	5′-CTGAGAGAGGTTACCAAG-3′
	Reverse	5′-CTTGGTAACCTCTCTCAG-3′

### Expression levels of candidate genes in maize lines having different starch granule sizes

In total, 30 maize lines with different starch granule sizes (small starch granule group: CIMBL140, CIMBL157, IRF314, CIMBL153, GEMS41, GEMS15, By804, GEMS55, BS16, and By813; medium starch granule group: 526018, Dong237, DH3732, GEMS23, TY5, CIMBL139, CIMBL102, Tie7922, GEMS17, and B113; large starch granule group: Dan360, CML325, GEMS54, CIMBL87, GEMS51, Zheng29, 835b, Ye8001, K22, and CIMBL10) were selected from the association panel to study the expression levels of candidate genes. Maize kernels (20 d after pollination) were used for RNA extraction according to the manufacturer’s user manual (Transgene Biotech, Beijing, China). The primers for the selected 10 candidate genes are shown in Table S6. A reverse transcription and fluorescence quantitative PCR analysis was conducted according to the user manual for the qPCR Master Mix (Vazyme Biotech, Nanjing, China). The actin gene was used as a reference, and all samples were analysed three times. The mean value of every sample was used for analysis.

## Electronic supplementary material

Supplementary Information

## Data Availability

https://pan.baidu.com/s/1bpFjeLD#list/path=%2F.
