# Supplementary material for: Identification of Quantitative Trait Loci and Candidate Genes for Maize Starch Granule Size through Association Mapping
Source: Sci Rep. 2018 Sep 24;8:14236. doi: 10.1038/s41598-018-31863-y (PMC6155146; doi:10.1038/s41598-018-31863-y)
Supplement: Supplementary file 1 — Supplementary Information [file 41598_2018_31863_MOESM1_ESM.pdf]

# Identification of Quantitative Trait Loci and Candidate Genes for Maize Starch Granule Size through Association Mapping

Na Liu, Zhanhui Zhang, Yadong Xue, Shujun Meng, Yubi Huang, Weihua Li, Jihong Huang*, Jihua Tang*

## Table S1 AVONA for starch granules length

| Varieties | Repeats | Average starch granules length | Standard Deviation | Standard Error | 95% confidence level | | Mini. value | Maxi. value |
|---|---|---|---|---|---|---|---|---|
| | | | | | Upper limit | Upper limit | | |
| TX5 | 10 | 12.5333 | .11547 | .06667 | 12.2465 | 12.8202 | 12.40 | 12.60 |
| Liao138 | 10 | 12.8000 | .10000 | .05774 | 12.5516 | 13.0484 | 12.70 | 12.90 |
| By807 | 10 | 10.2333 | .11547 | .06667 | 9.9465 | 10.5202 | 10.10 | 10.30 |
| GEMS18 | 10 | 11.6333 | .11547 | .06667 | 11.3465 | 11.9202 | 11.50 | 11.70 |
| GEMS29 | 10 | 12.0000 | .17321 | .10000 | 11.5697 | 12.4303 | 11.80 | 12.10 |
| CIMBL109 | 10 | 12.8333 | .05774 | .03333 | 12.6899 | 12.9768 | 12.80 | 12.90 |
| CML170 | 10 | 8.7667 | .04041 | .02333 | 8.6663 | 8.8671 | 8.72 | 8.79 |
| CIMBL2 | 10 | 10.2333 | .11547 | .06667 | 9.9465 | 10.5202 | 10.10 | 10.30 |
| Shen137 | 10 | 11.5667 | .11547 | .06667 | 11.2798 | 11.8535 | 11.50 | 11.70 |
| CIMBL77 | 10 | 9.4167 | .13317 | .07688 | 9.0859 | 9.7475 | 9.33 | 9.57 |
| CIMBL146 | 10 | 11.5000 | .10000 | .05774 | 11.2516 | 11.7484 | 11.40 | 11.60 |
| CIMBL17 | 10 | 12.5333 | .11547 | .06667 | 12.2465 | 12.8202 | 12.40 | 12.60 |
| CIMBL56 | 10 | 10.6333 | .11547 | .06667 | 10.3465 | 10.9202 | 10.50 | 10.70 |
| CML163 | 10 | 11.8667 | .05774 | .03333 | 11.7232 | 12.0101 | 11.80 | 11.90 |
| CIMBL154 | 10 | 10.6000 | .10000 | .05774 | 10.3516 | 10.8484 | 10.50 | 10.70 |
| CIMBL63 | 10 | 11.4000 | .00000 | .00000 | 11.4000 | 11.4000 | 11.40 | 11.40 |
| CML480 | 10 | 12.3667 | .15275 | .08819 | 11.9872 | 12.7461 | 12.20 | 12.50 |
| CIMBL22 | 10 | 11.6000 | .10000 | .05774 | 11.3516 | 11.8484 | 11.50 | 11.70 |
| CML118 | 10 | 11.4667 | .05774 | .03333 | 11.3232 | 11.6101 | 11.40 | 11.50 |
| CML223 | 10 | 11.1333 | .30551 | .17638 | 10.3744 | 11.8922 | 10.80 | 11.40 |
| Qi319 | 10 | 11.1667 | .25166 | .14530 | 10.5415 | 11.7918 | 10.90 | 11.40 |
| CIMBL52 | 10 | 10.5333 | .15275 | .08819 | 10.1539 | 10.9128 | 10.40 | 10.70 |
| CIMBL120 | 10 | 13.2000 | .30000 | .17321 | 12.4548 | 13.9452 | 12.90 | 13.50 |
| CML479 | 10 | 8.2600 | .09644 | .05568 | 8.0204 | 8.4996 | 8.19 | 8.37 |
| CML122 | 10 | 9.1833 | .19858 | .11465 | 8.6900 | 9.6766 | 9.04 | 9.41 |
| CML31 | 10 | 12.7667 | .15275 | .08819 | 12.3872 | 13.1461 | 12.60 | 12.90 |
| CML324 | 10 | 12.5667 | .11547 | .06667 | 12.2798 | 12.8535 | 12.50 | 12.70 |
| CIMBL30 | 10 | 7.4067 | .09866 | .05696 | 7.1616 | 7.6517 | 7.34 | 7.52 |
| CIMBL91 | 10 | 13.2333 | .20817 | .12019 | 12.7162 | 13.7504 | 13.00 | 13.40 |
| CIMBL143 | 10 | 10.4333 | .05774 | .03333 | 10.2899 | 10.5768 | 10.40 | 10.50 |
| CIMBL84 | 10 | 15.1333 | .20817 | .12019 | 14.6162 | 15.6504 | 14.90 | 15.30 |
| CIMBL60 | 10 | 12.5000 | .10000 | .05774 | 12.2516 | 12.7484 | 12.40 | 12.60 |
| CIMBL112 | 10 | 9.7433 | .09074 | .05239 | 9.5179 | 9.9687 | 9.66 | 9.84 |
| CIMBL150 | 10 | 12.0333 | .15275 | .08819 | 11.6539 | 12.4128 | 11.90 | 12.20 |

| | | | | | | | | |
|---|---|---|---|---|---|---|---|---|
| CIMBL97 | 10 | 11.9000 | .26458 | .15275 | 11.2428 | 12.5572 | 11.70 | 12.20 |
| CIMBL67 | 10 | 11.6333 | .05774 | .03333 | 11.4899 | 11.7768 | 11.60 | 11.70 |
| CIMBL1 | 10 | 12.3667 | .11547 | .06667 | 12.0798 | 12.6535 | 12.30 | 12.50 |
| CIMBL76 | 10 | 12.1667 | .05774 | .03333 | 12.0232 | 12.3101 | 12.10 | 12.20 |
| CIMBL9 | 10 | 13.3000 | .20000 | .11547 | 12.8032 | 13.7968 | 13.10 | 13.50 |
| CML165 | 10 | 10.9000 | .10000 | .05774 | 10.6516 | 11.1484 | 10.80 | 11.00 |
| CML470 | 10 | 16.1000 | .10000 | .05774 | 15.8516 | 16.3484 | 16.00 | 16.20 |
| CIMBL54 | 10 | 13.7000 | .34641 | .20000 | 12.8395 | 14.5605 | 13.50 | 14.10 |
| CIMBL80 | 10 | 9.7333 | .05774 | .03333 | 9.5899 | 9.8768 | 9.70 | 9.80 |
| CIMBL96 | 10 | 11.1000 | .00000 | .00000 | 11.1000 | 11.1000 | 11.10 | 11.10 |
| CIMBL127 | 10 | 8.6267 | .07767 | .04485 | 8.4337 | 8.8196 | 8.54 | 8.69 |
| CIMBL136 | 10 | 9.9400 | .23065 | .13317 | 9.3670 | 10.5130 | 9.76 | 10.20 |
| CIMBL106 | 10 | 10.7333 | .15275 | .08819 | 10.3539 | 11.1128 | 10.60 | 10.90 |
| CML304 | 10 | 12.6333 | .15275 | .08819 | 12.2539 | 13.0128 | 12.50 | 12.80 |
| CIMBL122 | 10 | 13.0000 | .26458 | .15275 | 12.3428 | 13.6572 | 12.80 | 13.30 |
| CIMBL58 | 10 | 12.9333 | .11547 | .06667 | 12.6465 | 13.2202 | 12.80 | 13.00 |
| CIMBL142 | 10 | 14.3667 | .20817 | .12019 | 13.8496 | 14.8838 | 14.20 | 14.60 |
| Yun46 | 10 | 11.4333 | .35119 | .20276 | 10.5609 | 12.3057 | 11.10 | 11.80 |
| CIMBL117 | 10 | 11.5000 | .10000 | .05774 | 11.2516 | 11.7484 | 11.40 | 11.60 |
| CIMBL83 | 10 | 11.8000 | .17321 | .10000 | 11.3697 | 12.2303 | 11.60 | 11.90 |
| CIMBL93 | 10 | 13.2000 | .52915 | .30551 | 11.8855 | 14.5145 | 12.60 | 13.60 |
| CIMBL48 | 10 | 13.0000 | .10000 | .05774 | 12.7516 | 13.2484 | 12.90 | 13.10 |
| CIMBL59 | 10 | 10.2667 | .05774 | .03333 | 10.1232 | 10.4101 | 10.20 | 10.30 |
| CIMBL92 | 10 | 9.7400 | .15133 | .08737 | 9.3641 | 10.1159 | 9.62 | 9.91 |
| 647 | 10 | 13.2000 | .20000 | .11547 | 12.7032 | 13.6968 | 13.00 | 13.40 |
| CML121 | 10 | 13.2333 | .20817 | .12019 | 12.7162 | 13.7504 | 13.00 | 13.40 |
| Nan21-3 | 10 | 7.0667 | .04933 | .02848 | 6.9441 | 7.1892 | 7.01 | 7.10 |
| CML162 | 10 | 10.3000 | .10000 | .05774 | 10.0516 | 10.5484 | 10.20 | 10.40 |
| CIMBL72 | 10 | 8.7333 | .05508 | .03180 | 8.5965 | 8.8701 | 8.68 | 8.79 |
| CIMBL107 | 10 | 8.8900 | .12166 | .07024 | 8.5878 | 9.1922 | 8.75 | 8.97 |
| CIMBL148 | 10 | 13.8333 | .20817 | .12019 | 13.3162 | 14.3504 | 13.60 | 14.00 |
| CIMBL10 | 10 | 9.3367 | .02517 | .01453 | 9.2742 | 9.3992 | 9.31 | 9.36 |
| P138 | 10 | 11.5667 | .15275 | .08819 | 11.1872 | 11.9461 | 11.40 | 11.70 |
| CIMBL139 | 10 | 10.3333 | .15275 | .08819 | 9.9539 | 10.7128 | 10.20 | 10.50 |
| CIMBL145 | 10 | 9.5967 | .06429 | .03712 | 9.4370 | 9.7564 | 9.55 | 9.67 |
| CIMBL126 | 10 | 11.6000 | .10000 | .05774 | 11.3516 | 11.8484 | 11.50 | 11.70 |
| CIMBL102 | 10 | 10.0067 | .09018 | .05207 | 9.7826 | 10.2307 | 9.92 | 10.10 |
| CML139 | 10 | 10.1267 | .12702 | .07333 | 9.8111 | 10.4422 | 9.98 | 10.20 |
| CML168 | 10 | 10.2067 | .29006 | .16746 | 9.4861 | 10.9272 | 9.92 | 10.50 |
| CML113 | 10 | 13.0000 | .17321 | .10000 | 12.5697 | 13.4303 | 12.90 | 13.20 |
| CML426 | 10 | 7.6667 | .10693 | .06173 | 7.4010 | 7.9323 | 7.55 | 7.76 |
| CIMBL140 | 10 | 9.8633 | .21127 | .12197 | 9.3385 | 10.3881 | 9.62 | 10.00 |
| CML338 | 10 | 13.6667 | .35119 | .20276 | 12.7943 | 14.5391 | 13.30 | 14.00 |

| | | | | | | | | |
|---|---|---|---|---|---|---|---|---|
| CIMBL88 | 10 | 10.1333 | .11547 | .06667 | 9.8465 | 10.4202 | 10.00 | 10.20 |
| CML423 | 10 | 8.6967 | .07638 | .04410 | 8.5069 | 8.8864 | 8.63 | 8.78 |
| CIMBL153 | 10 | 9.4000 | .15100 | .08718 | 9.0249 | 9.7751 | 9.26 | 9.56 |
| CIMBL71 | 10 | 11.5333 | .25166 | .14530 | 10.9082 | 12.1585 | 11.30 | 11.80 |
| CIMBL144 | 10 | 10.4333 | .20817 | .12019 | 9.9162 | 10.9504 | 10.20 | 10.60 |
| CIMBL138 | 10 | 11.9000 | .40000 | .23094 | 10.9063 | 12.8937 | 11.50 | 12.30 |
| CIMBL50 | 10 | 9.8067 | .12583 | .07265 | 9.4941 | 10.1192 | 9.69 | 9.94 |
| CIMBL4 | 10 | 13.2000 | .10000 | .05774 | 12.9516 | 13.4484 | 13.10 | 13.30 |
| CIMBL23 | 10 | 12.3333 | .11547 | .06667 | 12.0465 | 12.6202 | 12.20 | 12.40 |
| CIMBL105 | 10 | 12.4333 | .50332 | .29059 | 11.1830 | 13.6837 | 11.90 | 12.90 |
| CIMBL49 | 10 | 12.2333 | .11547 | .06667 | 11.9465 | 12.5202 | 12.10 | 12.30 |
| CIMBL8 | 10 | 10.8667 | .05774 | .03333 | 10.7232 | 11.0101 | 10.80 | 10.90 |
| CIMBL98 | 10 | 14.4000 | .10000 | .05774 | 14.1516 | 14.6484 | 14.30 | 14.50 |
| CIMBL12 | 10 | 10.5333 | .15275 | .08819 | 10.1539 | 10.9128 | 10.40 | 10.70 |
| CIMBL61 | 10 | 9.0800 | .13748 | .07937 | 8.7385 | 9.4215 | 8.93 | 9.20 |
| CIMBL66 | 10 | 13.4000 | .10000 | .05774 | 13.1516 | 13.6484 | 13.30 | 13.50 |
| CIMBL87 | 10 | 8.1800 | .16000 | .09238 | 7.7825 | 8.5775 | 8.02 | 8.34 |
| CIMBL157 | 10 | 11.8667 | .11547 | .06667 | 11.5798 | 12.1535 | 11.80 | 12.00 |
| CIMBL45 | 10 | 11.5000 | .36056 | .20817 | 10.6043 | 12.3957 | 11.10 | 11.80 |
| CML290 | 10 | 12.4333 | .32146 | .18559 | 11.6348 | 13.2319 | 12.20 | 12.80 |
| CIMBL11 | 10 | 11.9667 | .05774 | .03333 | 11.8232 | 12.1101 | 11.90 | 12.00 |
| CIMBL27 | 10 | 13.5333 | .15275 | .08819 | 13.1539 | 13.9128 | 13.40 | 13.70 |
| CIMBL21 | 10 | 14.1333 | .25166 | .14530 | 13.5082 | 14.7585 | 13.90 | 14.40 |
| CML325 | 10 | 11.8000 | .10000 | .05774 | 11.5516 | 12.0484 | 11.70 | 11.90 |
| CML473 | 10 | 11.5333 | .15275 | .08819 | 11.1539 | 11.9128 | 11.40 | 11.70 |
| L3180 | 10 | 15.0333 | .72342 | .41767 | 13.2363 | 16.8304 | 14.20 | 15.50 |
| Xun971 | 10 | 14.1667 | .50332 | .29059 | 12.9163 | 15.4170 | 13.70 | 14.70 |
| Ye478 | 10 | 12.1667 | .05774 | .03333 | 12.0232 | 12.3101 | 12.10 | 12.20 |
| TY3 | 10 | 13.7667 | .15275 | .08819 | 13.3872 | 14.1461 | 13.60 | 13.90 |
| K22 | 10 | 10.2800 | .33045 | .19079 | 9.4591 | 11.1009 | 9.94 | 10.60 |
| JH96C | 10 | 13.8000 | .10000 | .05774 | 13.5516 | 14.0484 | 13.70 | 13.90 |
| TT16 | 10 | 13.2000 | .10000 | .05774 | 12.9516 | 13.4484 | 13.10 | 13.30 |
| Zheng653 | 10 | 15.7000 | .51962 | .30000 | 14.4092 | 16.9908 | 15.10 | 16.00 |
| zheng58 | 10 | 11.5667 | .05774 | .03333 | 11.4232 | 11.7101 | 11.50 | 11.60 |
| GEMS53 | 10 | 11.6000 | .10000 | .05774 | 11.3516 | 11.8484 | 11.50 | 11.70 |
| ZZ03 | 10 | 11.6333 | .11547 | .06667 | 11.3465 | 11.9202 | 11.50 | 11.70 |
| Sy1035 | 10 | 10.2667 | .20817 | .12019 | 9.7496 | 10.7838 | 10.10 | 10.50 |
| Ji53 | 10 | 12.6333 | .30551 | .17638 | 11.8744 | 13.3922 | 12.30 | 12.90 |
| Ye52106 | 10 | 11.9000 | .10000 | .05774 | 11.6516 | 12.1484 | 11.80 | 12.00 |
| Wu109 | 10 | 12.7667 | .11547 | .06667 | 12.4798 | 13.0535 | 12.70 | 12.90 |
| By843 | 10 | 11.8667 | .05774 | .03333 | 11.7232 | 12.0101 | 11.80 | 11.90 |
| GEMS25 | 10 | 12.0667 | .20817 | .12019 | 11.5496 | 12.5838 | 11.90 | 12.30 |
| Ry737 | 10 | 12.8667 | .05774 | .03333 | 12.7232 | 13.0101 | 12.80 | 12.90 |

| | | | | | | | | |
|---|---|---|---|---|---|---|---|---|
| Sy3073 | 10 | 12.2667 | .05774 | .03333 | 12.1232 | 12.4101 | 12.20 | 12.30 |
| Sy1052 | 10 | 12.3333 | .15275 | .08819 | 11.9539 | 12.7128 | 12.20 | 12.50 |
| 7381 | 10 | 13.6000 | .10000 | .05774 | 13.3516 | 13.8484 | 13.50 | 13.70 |
| Qi205 | 10 | 11.0667 | .15275 | .08819 | 10.6872 | 11.4461 | 10.90 | 11.20 |
| Liao5263 | 10 | 12.8667 | .20817 | .12019 | 12.3496 | 13.3838 | 12.70 | 13.10 |
| 1323 | 10 | 13.0667 | .05774 | .03333 | 12.9232 | 13.2101 | 13.00 | 13.10 |
| ZZ01 | 10 | 9.6100 | .10583 | .06110 | 9.3471 | 9.8729 | 9.53 | 9.73 |
| LXN | 10 | 13.3333 | .05774 | .03333 | 13.1899 | 13.4768 | 13.30 | 13.40 |
| By4839 | 10 | 12.9667 | .05774 | .03333 | 12.8232 | 13.1101 | 12.90 | 13.00 |
| U8112 | 10 | 12.6500 | .07071 | .05000 | 12.0147 | 13.2853 | 12.60 | 12.70 |
| 4019 | 10 | 12.1000 | .10000 | .05774 | 11.8516 | 12.3484 | 12.00 | 12.20 |
| Ji853 | 10 | 14.1000 | .26458 | .15275 | 13.4428 | 14.7572 | 13.90 | 14.40 |
| Zhong69 | 10 | 11.6333 | .15275 | .08819 | 11.2539 | 12.0128 | 11.50 | 11.80 |
| GEMS30 | 10 | 11.0667 | .15275 | .08819 | 10.6872 | 11.4461 | 10.90 | 11.20 |
| 7884-4Ht | 10 | 10.8000 | .10000 | .05774 | 10.5516 | 11.0484 | 10.70 | 10.90 |
| Gy462 | 10 | 10.4667 | .35119 | .20276 | 9.5943 | 11.3391 | 10.10 | 10.80 |
| chuan48-2 | 10 | 11.4000 | .17321 | .10000 | 10.9697 | 11.8303 | 11.20 | 11.50 |
| GEMS14 | 10 | 12.2333 | .20817 | .12019 | 11.7162 | 12.7504 | 12.00 | 12.40 |
| GEMS17 | 10 | 12.4000 | .26458 | .15275 | 11.7428 | 13.0572 | 12.20 | 12.70 |
| Liao159 | 10 | 10.3975 | 1.40239 | .70119 | 8.1660 | 12.6290 | 9.66 | 12.50 |
| Dan4245 | 10 | 13.6667 | .11547 | .06667 | 13.3798 | 13.9535 | 13.60 | 13.80 |
| 835b | 10 | 11.0333 | .15275 | .08819 | 10.6539 | 11.4128 | 10.90 | 11.20 |
| GEMS49 | 10 | 11.3333 | .05774 | .03333 | 11.1899 | 11.4768 | 11.30 | 11.40 |
| By809 | 10 | 12.3333 | .15275 | .08819 | 11.9539 | 12.7128 | 12.20 | 12.50 |
| Si444 | 10 | 13.9667 | .05774 | .03333 | 13.8232 | 14.1101 | 13.90 | 14.00 |
| 04K5702 | 10 | 10.4333 | .05774 | .03333 | 10.2899 | 10.5768 | 10.40 | 10.50 |
| GEMS47 | 10 | 9.7900 | .02646 | .01528 | 9.7243 | 9.8557 | 9.76 | 9.81 |
| WMR | 10 | 15.8333 | .15275 | .08819 | 15.4539 | 16.2128 | 15.70 | 16.00 |
| FCD0602 | 10 | 9.8200 | .18055 | .09028 | 9.5327 | 10.1073 | 9.59 | 10.00 |
| 04K5672 | 10 | 11.0333 | .15275 | .08819 | 10.6539 | 11.4128 | 10.90 | 11.20 |
| Dan3130 | 10 | 13.3000 | .36056 | .20817 | 12.4043 | 14.1957 | 12.90 | 13.60 |
| 9642 | 10 | 14.1333 | .11547 | .06667 | 13.8465 | 14.4202 | 14.00 | 14.20 |
| Ye8001 | 10 | 12.8667 | .35119 | .20276 | 11.9943 | 13.7391 | 12.50 | 13.20 |
| Mo17 | 10 | 12.1667 | .15275 | .08819 | 11.7872 | 12.5461 | 12.00 | 12.30 |
| TY6 | 10 | 12.9667 | .30551 | .17638 | 12.2078 | 13.7256 | 12.70 | 13.30 |
| GEMS5 | 10 | 11.9000 | .10000 | .05774 | 11.6516 | 12.1484 | 11.80 | 12.00 |
| Dan340 | 10 | 10.5667 | .05774 | .03333 | 10.4232 | 10.7101 | 10.50 | 10.60 |
| Sy1039 | 10 | 10.9667 | .05774 | .03333 | 10.8232 | 11.1101 | 10.90 | 11.00 |
| GEMS12 | 10 | 8.8733 | .05033 | .02906 | 8.7483 | 8.9984 | 8.82 | 8.92 |
| B111 | 10 | 8.7967 | .14572 | .08413 | 8.4347 | 9.1586 | 8.63 | 8.90 |
| K10 | 10 | 13.4000 | .10000 | .05774 | 13.1516 | 13.6484 | 13.30 | 13.50 |
| DSB | 10 | 11.8333 | .05774 | .03333 | 11.6899 | 11.9768 | 11.80 | 11.90 |
| DH3732 | 10 | 15.3667 | .15275 | .08819 | 14.9872 | 15.7461 | 15.20 | 15.50 |

| | | | | | | | |
|---|---|---|---|---|---|---|---|
| GEMS51 | 10 | 12.6667 | .15275 | .08819 | 12.2872 | 13.0461 | 12.50 | 12.80 |
| Liao5114 | 10 | 11.1000 | .10000 | .05774 | 10.8516 | 11.3484 | 11.00 | 11.20 |
| Z2018F | 10 | 8.7800 | .16093 | .09292 | 8.3802 | 9.1798 | 8.63 | 8.95 |
| BS16 | 10 | 10.6667 | .05774 | .03333 | 10.5232 | 10.8101 | 10.60 | 10.70 |
| Dan598 | 10 | 8.8967 | .03055 | .01764 | 8.8208 | 8.9726 | 8.87 | 8.93 |
| By804 | 10 | 10.9667 | .11547 | .06667 | 10.6798 | 11.2535 | 10.90 | 11.10 |
| TY7 | 10 | 11.3667 | .30551 | .17638 | 10.6078 | 12.1256 | 11.10 | 11.70 |
| Gy246 | 10 | 11.9667 | .20817 | .12019 | 11.4496 | 12.4838 | 11.80 | 12.20 |
| 975-12 | 10 | 12.0667 | .15275 | .08819 | 11.6872 | 12.4461 | 11.90 | 12.20 |
| TY1 | 10 | 11.9667 | .11547 | .06667 | 11.6798 | 12.2535 | 11.90 | 12.10 |
| GEMS65 | 10 | 15.5000 | .26458 | .15275 | 14.8428 | 16.1572 | 15.20 | 15.70 |
| Dan360 | 10 | 12.1333 | .15275 | .08819 | 11.7539 | 12.5128 | 12.00 | 12.30 |
| MO113 | 10 | 12.3000 | .36056 | .20817 | 11.4043 | 13.1957 | 12.00 | 12.70 |
| 3411 | 10 | 8.5567 | .20429 | .11795 | 8.0492 | 9.0641 | 8.41 | 8.79 |
| GEMS41 | 10 | 11.3667 | .20817 | .12019 | 10.8496 | 11.8838 | 11.20 | 11.60 |
| ES40 | 10 | 9.6067 | .14012 | .08090 | 9.2586 | 9.9547 | 9.47 | 9.75 |
| J4112 | 10 | 11.9000 | .17321 | .10000 | 11.4697 | 12.3303 | 11.70 | 12.00 |
| CF3 | 10 | 9.5467 | .20207 | .11667 | 9.0447 | 10.0486 | 9.43 | 9.78 |
| Gy220 | 10 | 11.0333 | .30551 | .17638 | 10.2744 | 11.7922 | 10.70 | 11.30 |
| GEMS52 | 10 | 9.9000 | .09539 | .05508 | 9.6630 | 10.1370 | 9.79 | 9.96 |
| Gy923 | 10 | 11.8667 | .32146 | .18559 | 11.0681 | 12.6652 | 11.50 | 12.10 |
| GY386B | 10 | 9.2567 | .17474 | .10088 | 8.8226 | 9.6907 | 9.11 | 9.45 |
| GEMS55 | 10 | 11.0000 | .20000 | .11547 | 10.5032 | 11.4968 | 10.80 | 11.20 |
| GEMS31 | 10 | 11.0333 | .15275 | .08819 | 10.6539 | 11.4128 | 10.90 | 11.20 |
| Ry684 | 10 | 11.2667 | .15275 | .08819 | 10.8872 | 11.6461 | 11.10 | 11.40 |
| Zi330 | 10 | 11.5667 | .11547 | .06667 | 11.2798 | 11.8535 | 11.50 | 11.70 |
| 526018 | 10 | 12.5333 | .32146 | .18559 | 11.7348 | 13.3319 | 12.30 | 12.90 |
| W138 | 10 | 11.7000 | .17321 | .10000 | 11.2697 | 12.1303 | 11.50 | 11.80 |
| GEMS27 | 10 | 12.1333 | .41633 | .24037 | 11.0991 | 13.1676 | 11.80 | 12.60 |
| JH59 | 10 | 11.7333 | .15275 | .08819 | 11.3539 | 12.1128 | 11.60 | 11.90 |
| TY5 | 10 | 9.2100 | .16643 | .09609 | 8.7966 | 9.6234 | 9.02 | 9.33 |
| GEMS39 | 10 | 8.9500 | .07810 | .04509 | 8.7560 | 9.1440 | 8.86 | 9.00 |
| GEMS15 | 10 | 8.3367 | .11930 | .06888 | 8.0403 | 8.6330 | 8.20 | 8.42 |
| Ji846 | 10 | 11.7667 | .11547 | .06667 | 11.4798 | 12.0535 | 11.70 | 11.90 |
| 5213 | 10 | 12.7000 | .43589 | .25166 | 11.6172 | 13.7828 | 12.40 | 13.20 |
| Zong31 | 10 | 9.4650 | .00707 | .00500 | 9.4015 | 9.5285 | 9.46 | 9.47 |
| 150 | 10 | 11.7333 | .25166 | .14530 | 11.1082 | 12.3585 | 11.50 | 12.00 |
| GEMS4 | 10 | 14.7333 | .05774 | .03333 | 14.5899 | 14.8768 | 14.70 | 14.80 |
| M153 | 10 | 10.0933 | .35346 | .20407 | 9.2153 | 10.9714 | 9.86 | 10.50 |
| TY8 | 10 | 13.4667 | .37859 | .21858 | 12.5262 | 14.4071 | 13.20 | 13.90 |
| GEMS54 | 10 | 12.0333 | .28868 | .16667 | 11.3162 | 12.7504 | 11.70 | 12.20 |
| GEMS23 | 10 | 10.3000 | .10000 | .05774 | 10.0516 | 10.5484 | 10.20 | 10.40 |
| GEMS11 | 10 | 8.6400 | .28513 | .16462 | 7.9317 | 9.3483 | 8.35 | 8.92 |

| | | | | | | | | |
|---|---|---|---|---|---|---|---|---|
| IRF314 | 10 | 13.5000 | .20000 | .11547 | 13.0032 | 13.9968 | 13.30 | 13.70 |
| BGY | 10 | 11.1000 | .30000 | .17321 | 10.3548 | 11.8452 | 10.80 | 11.40 |
| TY2 | 10 | 8.6500 | .14731 | .08505 | 8.2841 | 9.0159 | 8.48 | 8.74 |
| Shen135 | 10 | 12.3000 | .10000 | .05774 | 12.0516 | 12.5484 | 12.20 | 12.40 |
| JY01 | 10 | 9.2733 | .15044 | .08686 | 8.8996 | 9.6471 | 9.13 | 9.43 |
| Zheng35 | 10 | 12.1000 | .17321 | .10000 | 11.6697 | 12.5303 | 11.90 | 12.20 |
| TY4 | 10 | 12.7000 | .10000 | .05774 | 12.4516 | 12.9484 | 12.60 | 12.80 |
| GEMS48 | 10 | 10.0667 | .05774 | .03333 | 9.9232 | 10.2101 | 10.00 | 10.10 |
| LK11 | 10 | 11.9000 | .87178 | .50332 | 9.7344 | 14.0656 | 10.90 | 12.50 |
| Tie7922 | 10 | 11.3667 | .15275 | .08819 | 10.9872 | 11.7461 | 11.20 | 11.50 |
| LY042 | 10 | 10.8333 | .40415 | .23333 | 9.8294 | 11.8373 | 10.40 | 11.20 |
| Sy1077 | 10 | 11.5000 | .55678 | .32146 | 10.1169 | 12.8831 | 11.00 | 12.10 |
| 384-2 | 10 | 11.3000 | .26458 | .15275 | 10.6428 | 11.9572 | 11.10 | 11.60 |
| Xi502 | 10 | 9.9567 | .05859 | .03383 | 9.8111 | 10.1022 | 9.89 | 10.00 |
| GEMS66 | 10 | 9.9467 | .18583 | .10729 | 9.4850 | 10.4083 | 9.74 | 10.10 |
| GEMS58 | 10 | 11.2333 | .15275 | .08819 | 10.8539 | 11.6128 | 11.10 | 11.40 |
| BZN | 10 | 10.0967 | .17898 | .10333 | 9.6521 | 10.5413 | 9.89 | 10.20 |
| GEMS1 | 10 | 8.5100 | .15395 | .08888 | 8.1276 | 8.8924 | 8.34 | 8.64 |
| Sy999 | 10 | 9.5267 | .10408 | .06009 | 9.2681 | 9.7852 | 9.41 | 9.61 |
| By855 | 10 | 12.9333 | .35119 | .20276 | 12.0609 | 13.8057 | 12.60 | 13.30 |
| GEMS6 | 10 | 13.2000 | .17321 | .10000 | 12.7697 | 13.6303 | 13.00 | 13.30 |
| Si446 | 10 | 12.4333 | .40415 | .23333 | 11.4294 | 13.4373 | 12.00 | 12.80 |
| B113 | 10 | 10.8333 | .25166 | .14530 | 10.2082 | 11.4585 | 10.60 | 11.10 |
| 812 | 10 | 10.6667 | .25166 | .14530 | 10.0415 | 11.2918 | 10.40 | 10.90 |
| 4F1 | 10 | 11.8667 | .15275 | .08819 | 11.4872 | 12.2461 | 11.70 | 12.00 |
| B151 | 10 | 13.2667 | .45092 | .26034 | 12.1465 | 14.3868 | 12.80 | 13.70 |
| B110 | 10 | 9.1333 | .21939 | .12667 | 8.5883 | 9.6783 | 8.88 | 9.26 |
| ZaC546 | 10 | 12.8000 | .26458 | .15275 | 12.1428 | 13.4572 | 12.50 | 13.00 |
| GEMS20 | 10 | 11.8000 | .17321 | .10000 | 11.3697 | 12.2303 | 11.60 | 11.90 |
| IRF291 | 10 | 10.5333 | .28868 | .16667 | 9.8162 | 11.2504 | 10.20 | 10.70 |
| MN | 10 | 13.0667 | .50332 | .29059 | 11.8163 | 14.3170 | 12.60 | 13.60 |
| TY11 | 10 | 12.4667 | .30551 | .17638 | 11.7078 | 13.2256 | 12.20 | 12.80 |
| HB | 10 | 9.9033 | .04619 | .02667 | 9.7886 | 10.0181 | 9.85 | 9.93 |
| 81162 | 10 | 14.1333 | .11547 | .06667 | 13.8465 | 14.4202 | 14.00 | 14.20 |
| Lv28 | 10 | 11.0667 | .66583 | .38442 | 9.4126 | 12.7207 | 10.50 | 11.80 |
| Zheng29 | 10 | 11.3000 | .00000 | .00000 | 11.3000 | 11.3000 | 11.30 | 11.30 |
| Tian77 | 10 | 10.0100 | .15588 | .09000 | 9.6228 | 10.3972 | 9.83 | 10.10 |
| 501 | 10 | 12.9667 | .30551 | .17638 | 12.2078 | 13.7256 | 12.70 | 13.30 |
| Si273 | 10 | 13.2667 | .32146 | .18559 | 12.4681 | 14.0652 | 12.90 | 13.50 |
| B73 | 10 | 7.4567 | .20429 | .11795 | 6.9492 | 7.9641 | 7.31 | 7.69 |
| 3H-2 | 10 | 10.7333 | .25166 | .14530 | 10.1082 | 11.3585 | 10.50 | 11.00 |
| Gy237 | 10 | 12.0667 | .11547 | .06667 | 11.7798 | 12.3535 | 12.00 | 12.20 |
| Ry732 | 10 | 12.3000 | .70000 | .40415 | 10.5611 | 14.0389 | 11.60 | 13.00 |

| | | | | | | | | |
|---|---|---|---|---|---|---|---|---|
| CML228 | 10 | 13.3667 | .49329 | .28480 | 12.1413 | 14.5921 | 12.80 | 13.70 |
| GEMS3 | 10 | 13.8333 | .68069 | .39299 | 12.1424 | 15.5243 | 13.30 | 14.60 |
| GEMS32 | 10 | 8.4067 | .15144 | .08743 | 8.0305 | 8.7829 | 8.30 | 8.58 |
| GEMS21 | 10 | 10.3667 | .47258 | .27285 | 9.1927 | 11.5406 | 10.00 | 10.90 |
| CIMBL38 | 10 | 11.5667 | .11547 | .06667 | 11.2798 | 11.8535 | 11.50 | 11.70 |
| CML189 | 10 | 12.7000 | .10000 | .05774 | 12.4516 | 12.9484 | 12.60 | 12.80 |
| CIMBL86 | 10 | 10.9000 | .17321 | .10000 | 10.4697 | 11.3303 | 10.80 | 11.10 |
| CML432 | 10 | 12.0333 | .37859 | .21858 | 11.0929 | 12.9738 | 11.60 | 12.30 |
| CIMBL85 | 10 | 13.1000 | .26458 | .15275 | 12.4428 | 13.7572 | 12.80 | 13.30 |
| K12 | 10 | 10.0267 | .31644 | .18270 | 9.2406 | 10.8127 | 9.68 | 10.30 |
| GEMS61 | 10 | 9.3900 | .05196 | .03000 | 9.2609 | 9.5191 | 9.33 | 9.42 |
| GEMS44 | 10 | 9.7467 | 1.09185 | .63038 | 7.0344 | 12.4590 | 8.62 | 10.80 |
| Sy1032 | 10 | 13.0333 | .37859 | .21858 | 12.0929 | 13.9738 | 12.60 | 13.30 |
| By813 | 10 | 11.7000 | .34641 | .20000 | 10.8395 | 12.5605 | 11.50 | 12.10 |
| GEMS28 | 10 | 13.1000 | .17321 | .10000 | 12.6697 | 13.5303 | 12.90 | 13.20 |
| D047 | 10 | 11.7333 | .11547 | .06667 | 11.4465 | 12.0202 | 11.60 | 11.80 |
| Total | 2660 | 11.5244 | 1.67929 | .05945 | 11.4077 | 11.6411 | 7.01 | 16.20 |

AVONA for starch granules width

| Varieties | Repeats | Average starch granules width | Standard Deviation | Standard Error | 95% confidence level | | Mini. value | Maxi. value |
|---|---|---|---|---|---|---|---|---|
| | | | | | Upper limit | Upper limit | | |
| TX5 | 10 | 12.5333 | .25166 | .14530 | 11.9082 | 13.1585 | 12.30 | 12.80 |
| Liao138 | 10 | 12.1333 | .11547 | .06667 | 11.8465 | 12.4202 | 12.00 | 12.20 |
| By807 | 10 | 9.4900 | .45211 | .26102 | 8.3669 | 10.6131 | 8.97 | 9.79 |
| GEMS18 | 10 | 10.3100 | .58506 | .33779 | 8.8566 | 11.7634 | 9.73 | 10.90 |
| GEMS29 | 10 | 11.1000 | .20000 | .11547 | 10.6032 | 11.5968 | 10.90 | 11.30 |
| CIMBL109 | 10 | 12.4333 | .15275 | .08819 | 12.0539 | 12.8128 | 12.30 | 12.60 |
| CML170 | 10 | 8.5833 | .02887 | .01667 | 8.5116 | 8.6550 | 8.55 | 8.60 |
| CIMBL2 | 10 | 9.5867 | .04509 | .02603 | 9.4747 | 9.6987 | 9.54 | 9.63 |
| Shen137 | 10 | 10.6000 | .62450 | .36056 | 9.0487 | 12.1513 | 10.10 | 11.30 |
| CIMBL77 | 10 | 8.4233 | .26502 | .15301 | 7.7650 | 9.0817 | 8.21 | 8.72 |
| CIMBL146 | 10 | 10.1333 | .05774 | .03333 | 9.9899 | 10.2768 | 10.10 | 10.20 |
| CIMBL17 | 10 | 11.3333 | .35119 | .20276 | 10.4609 | 12.2057 | 11.00 | 11.70 |
| CIMBL56 | 10 | 9.8400 | .06928 | .04000 | 9.6679 | 10.0121 | 9.80 | 9.92 |
| CML163 | 10 | 11.0333 | .23094 | .13333 | 10.4596 | 11.6070 | 10.90 | 11.30 |
| CIMBL154 | 10 | 8.4700 | .24269 | .14012 | 7.8671 | 9.0729 | 8.19 | 8.62 |
| CIMBL63 | 10 | 10.7333 | .15275 | .08819 | 10.3539 | 11.1128 | 10.60 | 10.90 |
| CML480 | 10 | 10.4000 | .36056 | .20817 | 9.5043 | 11.2957 | 10.10 | 10.80 |
| CIMBL22 | 10 | 11.3667 | .11547 | .06667 | 11.0798 | 11.6535 | 11.30 | 11.50 |
| CML118 | 10 | 9.5333 | .17388 | .10039 | 9.1014 | 9.9653 | 9.40 | 9.73 |
| CML223 | 10 | 10.5000 | .10000 | .05774 | 10.2516 | 10.7484 | 10.40 | 10.60 |
| Qi319 | 10 | 9.8300 | .24269 | .14012 | 9.2271 | 10.4329 | 9.55 | 9.98 |
| CIMBL52 | 10 | 9.6333 | .06110 | .03528 | 9.4816 | 9.7851 | 9.58 | 9.70 |
| CIMBL120 | 10 | 11.7000 | .20000 | .11547 | 11.2032 | 12.1968 | 11.50 | 11.90 |
| CML479 | 10 | 6.8567 | .30925 | .17854 | 6.0885 | 7.6249 | 6.60 | 7.20 |
| CML122 | 10 | 7.2600 | .26514 | .15308 | 6.6014 | 7.9186 | 7.03 | 7.55 |
| CML31 | 10 | 11.8000 | .00000 | .00000 | 11.8000 | 11.8000 | 11.80 | 11.80 |
| CML324 | 10 | 11.0333 | .05774 | .03333 | 10.8899 | 11.1768 | 11.00 | 11.10 |
| CIMBL30 | 10 | 7.0633 | .08505 | .04910 | 6.8521 | 7.2746 | 7.00 | 7.16 |
| CIMBL91 | 10 | 11.7333 | .11547 | .06667 | 11.4465 | 12.0202 | 11.60 | 11.80 |
| CIMBL143 | 10 | 9.4400 | .04583 | .02646 | 9.3262 | 9.5538 | 9.39 | 9.48 |
| CIMBL84 | 10 | 13.5000 | .17321 | .10000 | 13.0697 | 13.9303 | 13.30 | 13.60 |
| CIMBL60 | 10 | 11.1667 | .05774 | .03333 | 11.0232 | 11.3101 | 11.10 | 11.20 |
| CIMBL112 | 10 | 8.5533 | .02309 | .01333 | 8.4960 | 8.6107 | 8.54 | 8.58 |
| CIMBL150 | 10 | 10.8333 | .11547 | .06667 | 10.5465 | 11.1202 | 10.70 | 10.90 |
| CIMBL97 | 10 | 8.8500 | .14000 | .08083 | 8.5022 | 9.1978 | 8.71 | 8.99 |

| | | | | | | | | |
|---|---|---|---|---|---|---|---|---|
| CIMBL67 | 10 | 10.2333 | .05774 | .03333 | 10.0899 | 10.3768 | 10.20 | 10.30 |
| CIMBL1 | 10 | 10.7667 | .05774 | .03333 | 10.6232 | 10.9101 | 10.70 | 10.80 |
| CIMBL76 | 10 | 10.9333 | .05774 | .03333 | 10.7899 | 11.0768 | 10.90 | 11.00 |
| CIMBL9 | 10 | 11.4667 | .25166 | .14530 | 10.8415 | 12.0918 | 11.20 | 11.70 |
| CML165 | 10 | 10.0967 | .27025 | .15603 | 9.4253 | 10.7680 | 9.79 | 10.30 |
| CML470 | 10 | 13.9000 | .45826 | .26458 | 12.7616 | 15.0384 | 13.40 | 14.30 |
| CIMBL54 | 10 | 12.6333 | .11547 | .06667 | 12.3465 | 12.9202 | 12.50 | 12.70 |
| CIMBL80 | 10 | 9.1733 | .24090 | .13908 | 8.5749 | 9.7718 | 9.01 | 9.45 |
| CIMBL96 | 10 | 10.6000 | .10000 | .05774 | 10.3516 | 10.8484 | 10.50 | 10.70 |
| CIMBL127 | 10 | 8.2267 | .04619 | .02667 | 8.1119 | 8.3414 | 8.20 | 8.28 |
| CIMBL136 | 10 | 9.6067 | .06807 | .03930 | 9.4376 | 9.7758 | 9.53 | 9.66 |
| CIMBL106 | 10 | 9.9067 | .10066 | .05812 | 9.6566 | 10.1567 | 9.80 | 10.00 |
| CML304 | 10 | 11.6333 | .25166 | .14530 | 11.0082 | 12.2585 | 11.40 | 11.90 |
| CIMBL122 | 10 | 12.0333 | .20817 | .12019 | 11.5162 | 12.5504 | 11.80 | 12.20 |
| CIMBL58 | 10 | 11.3333 | .11547 | .06667 | 11.0465 | 11.6202 | 11.20 | 11.40 |
| CIMBL142 | 10 | 12.7667 | .15275 | .08819 | 12.3872 | 13.1461 | 12.60 | 12.90 |
| Yun46 | 10 | 10.2667 | .15275 | .08819 | 9.8872 | 10.6461 | 10.10 | 10.40 |
| CIMBL117 | 10 | 11.3000 | .10000 | .05774 | 11.0516 | 11.5484 | 11.20 | 11.40 |
| CIMBL83 | 10 | 11.6333 | .20817 | .12019 | 11.1162 | 12.1504 | 11.40 | 11.80 |
| CIMBL93 | 10 | 11.9333 | .15275 | .08819 | 11.5539 | 12.3128 | 11.80 | 12.10 |
| CIMBL48 | 10 | 11.5333 | .30551 | .17638 | 10.7744 | 12.2922 | 11.20 | 11.80 |
| CIMBL59 | 10 | 8.6100 | .12767 | .07371 | 8.2928 | 8.9272 | 8.50 | 8.75 |
| CIMBL92 | 10 | 9.1067 | .05686 | .03283 | 8.9654 | 9.2479 | 9.06 | 9.17 |
| 647 | 10 | 11.4000 | .10000 | .05774 | 11.1516 | 11.6484 | 11.30 | 11.50 |
| CML121 | 10 | 12.2667 | .28868 | .16667 | 11.5496 | 12.9838 | 12.10 | 12.60 |
| Nan21-3 | 10 | 7.0367 | .05508 | .03180 | 6.8999 | 7.1735 | 7.00 | 7.10 |
| CML162 | 10 | 10.3000 | .10000 | .05774 | 10.0516 | 10.5484 | 10.20 | 10.40 |
| CIMBL72 | 10 | 8.6600 | .14107 | .08145 | 8.3096 | 9.0104 | 8.51 | 8.79 |
| CIMBL107 | 10 | 8.4567 | .05686 | .03283 | 8.3154 | 8.5979 | 8.41 | 8.52 |
| CIMBL148 | 10 | 12.4667 | .11547 | .06667 | 12.1798 | 12.7535 | 12.40 | 12.60 |
| CIMBL10 | 10 | 9.1667 | .11547 | .06667 | 8.8798 | 9.4535 | 9.10 | 9.30 |
| P138 | 10 | 9.5400 | .40927 | .23629 | 8.5233 | 10.5567 | 9.19 | 9.99 |
| CIMBL139 | 10 | 9.1867 | .16042 | .09262 | 8.7882 | 9.5852 | 9.02 | 9.34 |
| CIMBL145 | 10 | 9.4867 | .04933 | .02848 | 9.3641 | 9.6092 | 9.43 | 9.52 |
| CIMBL126 | 10 | 10.6000 | .17321 | .10000 | 10.1697 | 11.0303 | 10.40 | 10.70 |
| CIMBL102 | 10 | 9.6267 | .25482 | .14712 | 8.9937 | 10.2597 | 9.46 | 9.92 |
| CML139 | 10 | 8.6267 | .30600 | .17667 | 7.8665 | 9.3868 | 8.45 | 8.98 |
| CML168 | 10 | 9.6333 | .07506 | .04333 | 9.4469 | 9.8198 | 9.59 | 9.72 |
| CML113 | 10 | 11.9667 | .15275 | .08819 | 11.5872 | 12.3461 | 11.80 | 12.10 |
| CML426 | 10 | 7.2900 | .11358 | .06557 | 7.0079 | 7.5721 | 7.16 | 7.37 |
| CIMBL140 | 10 | 8.6333 | .18475 | .10667 | 8.1744 | 9.0923 | 8.42 | 8.74 |
| CML338 | 10 | 12.7333 | .11547 | .06667 | 12.4465 | 13.0202 | 12.60 | 12.80 |
| CIMBL88 | 10 | 9.1067 | .00577 | .00333 | 9.0923 | 9.1210 | 9.10 | 9.11 |

| | | | | | | | | |
|---|---|---|---|---|---|---|---|---|
| CML423 | 10 | 8.3567 | .11504 | .06642 | 8.0709 | 8.6424 | 8.24 | 8.47 |
| CIMBL153 | 10 | 8.9733 | .05508 | .03180 | 8.8365 | 9.1101 | 8.91 | 9.01 |
| CIMBL71 | 10 | 10.6667 | .15275 | .08819 | 10.2872 | 11.0461 | 10.50 | 10.80 |
| CIMBL144 | 10 | 9.7400 | .19519 | .11269 | 9.2551 | 10.2249 | 9.54 | 9.93 |
| CIMBL138 | 10 | 11.4667 | .11547 | .06667 | 11.1798 | 11.7535 | 11.40 | 11.60 |
| CIMBL50 | 10 | 9.3000 | .36056 | .20817 | 8.4043 | 10.1957 | 8.90 | 9.60 |
| CIMBL4 | 10 | 10.9333 | .28868 | .16667 | 10.2162 | 11.6504 | 10.60 | 11.10 |
| CIMBL23 | 10 | 11.3000 | .20000 | .11547 | 10.8032 | 11.7968 | 11.10 | 11.50 |
| CIMBL105 | 10 | 11.8000 | .10000 | .05774 | 11.5516 | 12.0484 | 11.70 | 11.90 |
| CIMBL49 | 10 | 11.8000 | .00000 | .00000 | 11.8000 | 11.8000 | 11.80 | 11.80 |
| CIMBL8 | 10 | 10.8333 | .05774 | .03333 | 10.6899 | 10.9768 | 10.80 | 10.90 |
| CIMBL98 | 10 | 13.4333 | .05774 | .03333 | 13.2899 | 13.5768 | 13.40 | 13.50 |
| CIMBL12 | 10 | 10.2667 | .20817 | .12019 | 9.7496 | 10.7838 | 10.10 | 10.50 |
| CIMBL61 | 10 | 8.7633 | .04509 | .02603 | 8.6513 | 8.8753 | 8.72 | 8.81 |
| CIMBL66 | 10 | 11.6667 | .15275 | .08819 | 11.2872 | 12.0461 | 11.50 | 11.80 |
| CIMBL87 | 10 | 8.1800 | .16000 | .09238 | 7.7825 | 8.5775 | 8.02 | 8.34 |
| CIMBL157 | 10 | 10.4000 | .34641 | .20000 | 9.5395 | 11.2605 | 10.20 | 10.80 |
| CIMBL45 | 10 | 9.8367 | .13204 | .07623 | 9.5087 | 10.1647 | 9.72 | 9.98 |
| CML290 | 10 | 10.7333 | .05774 | .03333 | 10.5899 | 10.8768 | 10.70 | 10.80 |
| CIMBL11 | 10 | 10.8000 | .17321 | .10000 | 10.3697 | 11.2303 | 10.60 | 10.90 |
| CIMBL27 | 10 | 11.6333 | .11547 | .06667 | 11.3465 | 11.9202 | 11.50 | 11.70 |
| CIMBL21 | 10 | 12.6333 | .25166 | .14530 | 12.0082 | 13.2585 | 12.40 | 12.90 |
| CML325 | 10 | 11.5333 | .20817 | .12019 | 11.0162 | 12.0504 | 11.30 | 11.70 |
| CML473 | 10 | 12.5333 | .25166 | .14530 | 11.9082 | 13.1585 | 12.30 | 12.80 |
| L3180 | 10 | 10.6000 | .26458 | .15275 | 9.9428 | 11.2572 | 10.40 | 10.90 |
| Xun971 | 10 | 12.6000 | .30000 | .17321 | 11.8548 | 13.3452 | 12.30 | 12.90 |
| Ye478 | 10 | 12.5000 | .17321 | .10000 | 12.0697 | 12.9303 | 12.40 | 12.70 |
| TY3 | 10 | 10.7000 | .17321 | .10000 | 10.2697 | 11.1303 | 10.50 | 10.80 |
| K22 | 10 | 11.8667 | .05774 | .03333 | 11.7232 | 12.0101 | 11.80 | 11.90 |
| JH96C | 10 | 9.4833 | .28501 | .16455 | 8.7753 | 10.1913 | 9.20 | 9.77 |
| TT16 | 10 | 11.6000 | .20000 | .11547 | 11.1032 | 12.0968 | 11.40 | 11.80 |
| Zheng653 | 10 | 10.2000 | .10000 | .05774 | 9.9516 | 10.4484 | 10.10 | 10.30 |
| zheng58 | 10 | 12.5333 | .35119 | .20276 | 11.6609 | 13.4057 | 12.20 | 12.90 |
| GEMS53 | 10 | 11.5667 | .05774 | .03333 | 11.4232 | 11.7101 | 11.50 | 11.60 |
| ZZ03 | 10 | 9.6800 | .07937 | .04583 | 9.4828 | 9.8772 | 9.62 | 9.77 |
| Sy1035 | 10 | 10.2133 | .28024 | .16180 | 9.5172 | 10.9095 | 9.94 | 10.50 |
| Ji53 | 10 | 10.2667 | .20817 | .12019 | 9.7496 | 10.7838 | 10.10 | 10.50 |
| Ye52106 | 10 | 10.5000 | .10000 | .05774 | 10.2516 | 10.7484 | 10.40 | 10.60 |
| Wu109 | 10 | 11.6333 | .15275 | .08819 | 11.2539 | 12.0128 | 11.50 | 11.80 |
| By843 | 10 | 10.6333 | .15275 | .08819 | 10.2539 | 11.0128 | 10.50 | 10.80 |
| GEMS25 | 10 | 10.1333 | .20817 | .12019 | 9.6162 | 10.6504 | 9.90 | 10.30 |
| Ry737 | 10 | 11.9000 | .17321 | .10000 | 11.4697 | 12.3303 | 11.70 | 12.00 |
| Sy3073 | 10 | 12.7667 | .11547 | .06667 | 12.4798 | 13.0535 | 12.70 | 12.90 |

| | | | | | | | | |
|---|---|---|---|---|---|---|---|---|
| Sy1052 | 10 | 12.2667 | .05774 | .03333 | 12.1232 | 12.4101 | 12.20 | 12.30 |
| 7381 | 10 | 10.8000 | .00000 | .00000 | 10.8000 | 10.8000 | 10.80 | 10.80 |
| Qi205 | 10 | 12.4333 | .25166 | .14530 | 11.8082 | 13.0585 | 12.20 | 12.70 |
| Liao5263 | 10 | 10.4333 | .05774 | .03333 | 10.2899 | 10.5768 | 10.40 | 10.50 |
| 1323 | 10 | 11.1667 | .20817 | .12019 | 10.6496 | 11.6838 | 11.00 | 11.40 |
| ZZ01 | 10 | 11.6667 | .05774 | .03333 | 11.5232 | 11.8101 | 11.60 | 11.70 |
| LXN | 10 | 8.7633 | .18771 | .10837 | 8.2970 | 9.2296 | 8.56 | 8.93 |
| By4839 | 10 | 12.1333 | .25166 | .14530 | 11.5082 | 12.7585 | 11.90 | 12.40 |
| U8112 | 10 | 11.3667 | .32146 | .18559 | 10.5681 | 12.1652 | 11.00 | 11.60 |
| 4019 | 10 | 11.3000 | .00000 | .00000 | 11.3000 | 11.3000 | 11.30 | 11.30 |
| Ji853 | 10 | 10.5667 | .11547 | .06667 | 10.2798 | 10.8535 | 10.50 | 10.70 |
| Zhong69 | 10 | 12.4000 | .36056 | .20817 | 11.5043 | 13.2957 | 12.00 | 12.70 |
| GEMS30 | 10 | 11.6333 | .15275 | .08819 | 11.2539 | 12.0128 | 11.50 | 11.80 |
| 7884-4Ht | 10 | 10.3333 | .32146 | .18559 | 9.5348 | 11.1319 | 10.10 | 10.70 |
| Gy462 | 10 | 9.2033 | .12342 | .07126 | 8.8967 | 9.5099 | 9.10 | 9.34 |
| chuan48-2 | 10 | 9.8067 | .10017 | .05783 | 9.5578 | 10.0555 | 9.73 | 9.92 |
| GEMS14 | 10 | 11.1667 | .05774 | .03333 | 11.0232 | 11.3101 | 11.10 | 11.20 |
| GEMS17 | 10 | 9.7733 | .29143 | .16826 | 9.0494 | 10.4973 | 9.54 | 10.10 |
| Liao159 | 10 | 11.5333 | .20817 | .12019 | 11.0162 | 12.0504 | 11.30 | 11.70 |
| Dan4245 | 10 | 10.0975 | .80293 | .40146 | 8.8199 | 11.3751 | 9.66 | 11.30 |
| 835b | 10 | 12.4667 | .15275 | .08819 | 12.0872 | 12.8461 | 12.30 | 12.60 |
| GEMS49 | 10 | 9.7833 | .14503 | .08373 | 9.4231 | 10.1436 | 9.64 | 9.93 |
| By809 | 10 | 10.8333 | .05774 | .03333 | 10.6899 | 10.9768 | 10.80 | 10.90 |
| Si444 | 10 | 10.2133 | .38018 | .21949 | 9.2689 | 11.1577 | 9.84 | 10.60 |
| 04K5702 | 10 | 11.7667 | .11547 | .06667 | 11.4798 | 12.0535 | 11.70 | 11.90 |
| GEMS47 | 10 | 9.4867 | .05508 | .03180 | 9.3499 | 9.6235 | 9.43 | 9.54 |
| WMR | 10 | 8.7333 | .15503 | .08950 | 8.3482 | 9.1184 | 8.58 | 8.89 |
| FCD0602 | 10 | 13.2333 | .15275 | .08819 | 12.8539 | 13.6128 | 13.10 | 13.40 |
| 04K5672 | 10 | 8.6975 | .33797 | .16899 | 8.1597 | 9.2353 | 8.21 | 8.94 |
| Dan3130 | 10 | 9.3733 | .13051 | .07535 | 9.0491 | 9.6975 | 9.27 | 9.52 |
| 9642 | 10 | 12.2000 | .10000 | .05774 | 11.9516 | 12.4484 | 12.10 | 12.30 |
| Ye8001 | 10 | 13.6333 | .15275 | .08819 | 13.2539 | 14.0128 | 13.50 | 13.80 |
| Mo17 | 10 | 10.2333 | .15275 | .08819 | 9.8539 | 10.6128 | 10.10 | 10.40 |
| TY6 | 10 | 10.5667 | .25166 | .14530 | 9.9415 | 11.1918 | 10.30 | 10.80 |
| GEMS5 | 10 | 10.7000 | .00000 | .00000 | 10.7000 | 10.7000 | 10.70 | 10.70 |
| Dan340 | 10 | 11.2000 | .10000 | .05774 | 10.9516 | 11.4484 | 11.10 | 11.30 |
| Sy1039 | 10 | 9.6800 | .29138 | .16823 | 8.9562 | 10.4038 | 9.36 | 9.93 |
| GEMS12 | 10 | 10.1000 | .10000 | .05774 | 9.8516 | 10.3484 | 10.00 | 10.20 |
| B111 | 10 | 8.3600 | .25357 | .14640 | 7.7301 | 8.9899 | 8.07 | 8.54 |
| K10 | 10 | 7.2500 | .39585 | .22855 | 6.2666 | 8.2334 | 6.84 | 7.63 |
| DSB | 10 | 10.6000 | .20000 | .11547 | 10.1032 | 11.0968 | 10.40 | 10.80 |
| DH3732 | 10 | 11.6667 | .32146 | .18559 | 10.8681 | 12.4652 | 11.30 | 11.90 |
| GEMS51 | 10 | 13.2000 | .17321 | .10000 | 12.7697 | 13.6303 | 13.00 | 13.30 |

| | | | | | | | | |
|---|---|---|---|---|---|---|---|---|
| Liao5114 | 10 | 11.3000 | .17321 | .10000 | 10.8697 | 11.7303 | 11.10 | 11.40 |
| Z2018F | 10 | 10.6333 | .15275 | .08819 | 10.2539 | 11.0128 | 10.50 | 10.80 |
| BS16 | 10 | 8.5867 | .32347 | .18676 | 7.7831 | 9.3902 | 8.33 | 8.95 |
| Dan598 | 10 | 9.7267 | .27538 | .15899 | 9.0426 | 10.4107 | 9.41 | 9.91 |
| By804 | 10 | 8.8967 | .03055 | .01764 | 8.8208 | 8.9726 | 8.87 | 8.93 |
| TY7 | 10 | 10.1100 | .32909 | .19000 | 9.2925 | 10.9275 | 9.73 | 10.30 |
| Gy246 | 10 | 10.3333 | .15275 | .08819 | 9.9539 | 10.7128 | 10.20 | 10.50 |
| 975-12 | 10 | 9.6500 | .33808 | .19519 | 8.8102 | 10.4898 | 9.26 | 9.86 |
| TY1 | 10 | 10.6333 | .20817 | .12019 | 10.1162 | 11.1504 | 10.40 | 10.80 |
| GEMS65 | 10 | 9.8167 | .40723 | .23511 | 8.8051 | 10.8283 | 9.35 | 10.10 |
| Dan360 | 10 | 13.1333 | .05774 | .03333 | 12.9899 | 13.2768 | 13.10 | 13.20 |
| MO113 | 10 | 11.5667 | .25166 | .14530 | 10.9415 | 12.1918 | 11.30 | 11.80 |
| 3411 | 10 | 11.8333 | .15275 | .08819 | 11.4539 | 12.2128 | 11.70 | 12.00 |
| GEMS41 | 10 | 8.5567 | .20429 | .11795 | 8.0492 | 9.0641 | 8.41 | 8.79 |
| ES40 | 10 | 10.3000 | .10000 | .05774 | 10.0516 | 10.5484 | 10.20 | 10.40 |
| J4112 | 10 | 9.6067 | .14012 | .08090 | 9.2586 | 9.9547 | 9.47 | 9.75 |
| CF3 | 10 | 10.1900 | .51507 | .29738 | 8.9105 | 11.4695 | 9.67 | 10.70 |
| Gy220 | 10 | 8.4000 | .34395 | .19858 | 7.5456 | 9.2544 | 8.11 | 8.78 |
| GEMS52 | 10 | 10.6000 | .26458 | .15275 | 9.9428 | 11.2572 | 10.30 | 10.80 |
| Gy923 | 10 | 9.6367 | .33561 | .19376 | 8.8030 | 10.4704 | 9.29 | 9.96 |
| GY386B | 10 | 10.0167 | .17559 | .10138 | 9.5805 | 10.4529 | 9.85 | 10.20 |
| GEMS55 | 10 | 8.7933 | .14154 | .08172 | 8.4417 | 9.1449 | 8.63 | 8.88 |
| GEMS31 | 10 | 9.8933 | .34487 | .19911 | 9.0366 | 10.7500 | 9.52 | 10.20 |
| Ry684 | 10 | 11.0333 | .15275 | .08819 | 10.6539 | 11.4128 | 10.90 | 11.20 |
| Zi330 | 10 | 9.7033 | .54243 | .31317 | 8.3559 | 11.0508 | 9.24 | 10.30 |
| 526018 | 10 | 9.4933 | .19858 | .11465 | 9.0000 | 9.9866 | 9.32 | 9.71 |
| W138 | 10 | 12.5000 | .26458 | .15275 | 11.8428 | 13.1572 | 12.30 | 12.80 |
| GEMS27 | 10 | 11.3667 | .15275 | .08819 | 10.9872 | 11.7461 | 11.20 | 11.50 |
| JH59 | 10 | 10.1767 | .30436 | .17572 | 9.4206 | 10.9327 | 9.83 | 10.40 |
| TY5 | 10 | 10.0167 | .16073 | .09280 | 9.6174 | 10.4159 | 9.90 | 10.20 |
| GEMS39 | 10 | 9.0567 | .20744 | .11977 | 8.5413 | 9.5720 | 8.87 | 9.28 |
| GEMS15 | 10 | 8.9500 | .07810 | .04509 | 8.7560 | 9.1440 | 8.86 | 9.00 |
| Ji846 | 10 | 7.8267 | .40266 | .23247 | 6.8264 | 8.8269 | 7.40 | 8.20 |
| 5213 | 10 | 11.3000 | .51962 | .30000 | 10.0092 | 12.5908 | 10.70 | 11.60 |
| Zong31 | 10 | 11.4000 | .26458 | .15275 | 10.7428 | 12.0572 | 11.10 | 11.60 |
| 150 | 10 | 9.3300 | .18385 | .13000 | 7.6782 | 10.9818 | 9.20 | 9.46 |
| GEMS4 | 10 | 10.5000 | .36056 | .20817 | 9.6043 | 11.3957 | 10.10 | 10.80 |
| M153 | 10 | 13.4000 | .20000 | .11547 | 12.9032 | 13.8968 | 13.20 | 13.60 |
| TY8 | 10 | 9.5567 | .32868 | .18977 | 8.7402 | 10.3732 | 9.28 | 9.92 |
| GEMS54 | 10 | 11.7667 | .50332 | .29059 | 10.5163 | 13.0170 | 11.30 | 12.30 |
| GEMS23 | 10 | 9.9400 | .27713 | .16000 | 9.2516 | 10.6284 | 9.62 | 10.10 |
| GEMS11 | 10 | 9.9133 | .43016 | .24835 | 8.8448 | 10.9819 | 9.45 | 10.30 |
| IRF314 | 10 | 7.3500 | .06557 | .03786 | 7.1871 | 7.5129 | 7.28 | 7.41 |

| | | | | | | | | |
|---|---|---|---|---|---|---|---|---|
| BGY | 10 | 11.8333 | .05774 | .03333 | 11.6899 | 11.9768 | 11.80 | 11.90 |
| TY2 | 10 | 10.4000 | .17321 | .10000 | 9.9697 | 10.8303 | 10.20 | 10.50 |
| Shen135 | 10 | 7.9567 | .08327 | .04807 | 7.7498 | 8.1635 | 7.89 | 8.05 |
| JY01 | 10 | 9.8267 | .15011 | .08667 | 9.4538 | 10.1996 | 9.74 | 10.00 |
| Zheng35 | 10 | 9.2733 | .15044 | .08686 | 8.8996 | 9.6471 | 9.13 | 9.43 |
| TY4 | 10 | 9.6467 | .13429 | .07753 | 9.3131 | 9.9803 | 9.55 | 9.80 |
| GEMS48 | 10 | 10.4667 | .25166 | .14530 | 9.8415 | 11.0918 | 10.20 | 10.70 |
| LK11 | 10 | 10.0667 | .05774 | .03333 | 9.9232 | 10.2101 | 10.00 | 10.10 |
| Tie7922 | 10 | 10.7333 | .15275 | .08819 | 10.3539 | 11.1128 | 10.60 | 10.90 |
| LY042 | 10 | 10.5000 | .20000 | .11547 | 10.0032 | 10.9968 | 10.30 | 10.70 |
| Sy1077 | 10 | 9.5533 | .12055 | .06960 | 9.2539 | 9.8528 | 9.44 | 9.68 |
| 384-2 | 10 | 11.3667 | .35119 | .20276 | 10.4943 | 12.2391 | 11.00 | 11.70 |
| Xi502 | 10 | 11.2333 | .15275 | .08819 | 10.8539 | 11.6128 | 11.10 | 11.40 |
| GEMS66 | 10 | 9.1533 | .11590 | .06692 | 8.8654 | 9.4413 | 9.02 | 9.23 |
| GEMS58 | 10 | 8.4433 | .21733 | .12548 | 7.9034 | 8.9832 | 8.21 | 8.64 |
| BZN | 10 | 11.2333 | .15275 | .08819 | 10.8539 | 11.6128 | 11.10 | 11.40 |
| GEMS1 | 10 | 8.8533 | .22811 | .13170 | 8.2867 | 9.4200 | 8.59 | 8.99 |
| Sy999 | 10 | 8.0333 | .33858 | .19548 | 7.1923 | 8.8744 | 7.67 | 8.34 |
| By855 | 10 | 9.5267 | .10408 | .06009 | 9.2681 | 9.7852 | 9.41 | 9.61 |
| GEMS6 | 10 | 11.9000 | .10000 | .05774 | 11.6516 | 12.1484 | 11.80 | 12.00 |
| Si446 | 10 | 11.3333 | .25166 | .14530 | 10.7082 | 11.9585 | 11.10 | 11.60 |
| B113 | 10 | 10.9333 | .32146 | .18559 | 10.1348 | 11.7319 | 10.70 | 11.30 |
| 812 | 10 | 9.3933 | .31342 | .18095 | 8.6147 | 10.1719 | 9.11 | 9.73 |
| 4F1 | 10 | 9.5600 | .03606 | .02082 | 9.4704 | 9.6496 | 9.52 | 9.59 |
| B151 | 10 | 11.6000 | .26458 | .15275 | 10.9428 | 12.2572 | 11.40 | 11.90 |
| B110 | 10 | 11.6333 | .11547 | .06667 | 11.3465 | 11.9202 | 11.50 | 11.70 |
| ZaC546 | 10 | 8.6933 | .16166 | .09333 | 8.2918 | 9.0949 | 8.60 | 8.88 |
| GEMS20 | 10 | 11.9333 | .30551 | .17638 | 11.1744 | 12.6922 | 11.60 | 12.20 |
| IRF291 | 10 | 10.4333 | .45092 | .26034 | 9.3132 | 11.5535 | 10.00 | 10.90 |
| MN | 10 | 10.2000 | .00000 | .00000 | 10.2000 | 10.2000 | 10.20 | 10.20 |
| TY11 | 10 | 11.7000 | .10000 | .05774 | 11.4516 | 11.9484 | 11.60 | 11.80 |
| HB | 10 | 12.1333 | .11547 | .06667 | 11.8465 | 12.4202 | 12.00 | 12.20 |
| 81162 | 10 | 9.3633 | .01155 | .00667 | 9.3346 | 9.3920 | 9.35 | 9.37 |
| Lv28 | 10 | 12.1333 | .11547 | .06667 | 11.8465 | 12.4202 | 12.00 | 12.20 |
| Zheng29 | 10 | 10.3000 | .10000 | .05774 | 10.0516 | 10.5484 | 10.20 | 10.40 |
| Tian77 | 10 | 10.1150 | .40305 | .28500 | 6.4937 | 13.7363 | 9.83 | 10.40 |
| 501 | 10 | 9.0333 | .15044 | .08686 | 8.6596 | 9.4071 | 8.89 | 9.19 |
| Si273 | 10 | 11.8667 | .05774 | .03333 | 11.7232 | 12.0101 | 11.80 | 11.90 |
| B73 | 10 | 11.5000 | .26458 | .15275 | 10.8428 | 12.1572 | 11.30 | 11.80 |
| 3H-2 | 10 | 7.4567 | .20429 | .11795 | 6.9492 | 7.9641 | 7.31 | 7.69 |
| Gy237 | 10 | 9.6900 | .15716 | .09074 | 9.2996 | 10.0804 | 9.52 | 9.83 |
| Ry732 | 10 | 10.7667 | .15275 | .08819 | 10.3872 | 11.1461 | 10.60 | 10.90 |
| CML228 | 10 | 11.4333 | .11547 | .06667 | 11.1465 | 11.7202 | 11.30 | 11.50 |

| | | | | | | | | |
|---|---|---|---|---|---|---|---|---|
| GEMS3 | 10 | 11.8667 | .45092 | .26034 | 10.7465 | 12.9868 | 11.40 | 12.30 |
| GEMS32 | 10 | 11.7667 | .11547 | .06667 | 11.4798 | 12.0535 | 11.70 | 11.90 |
| GEMS21 | 10 | 8.4067 | .15144 | .08743 | 8.0305 | 8.7829 | 8.30 | 8.58 |
| CIMBL38 | 10 | 9.5500 | .33867 | .19553 | 8.7087 | 10.3913 | 9.28 | 9.93 |
| CML189 | 10 | 10.2333 | .11547 | .06667 | 9.9465 | 10.5202 | 10.10 | 10.30 |
| CIMBL86 | 10 | 12.7000 | .10000 | .05774 | 12.4516 | 12.9484 | 12.60 | 12.80 |
| CML432 | 10 | 10.3000 | .17321 | .10000 | 9.8697 | 10.7303 | 10.20 | 10.50 |
| CIMBL85 | 10 | 11.4000 | .40000 | .23094 | 10.4063 | 12.3937 | 11.00 | 11.80 |
| K12 | 10 | 11.8667 | .23094 | .13333 | 11.2930 | 12.4404 | 11.60 | 12.00 |
| GEMS61 | 10 | 8.8833 | .16803 | .09701 | 8.4659 | 9.3007 | 8.70 | 9.03 |
| GEMS44 | 10 | 8.1900 | .06928 | .04000 | 8.0179 | 8.3621 | 8.11 | 8.23 |
| Sy1032 | 10 | 8.5633 | .33858 | .19548 | 7.7223 | 9.4044 | 8.20 | 8.87 |
| By813 | 10 | 10.7667 | .98150 | .56667 | 8.3285 | 13.2048 | 10.20 | 11.90 |
| GEMS28 | 10 | 10.6667 | .11547 | .06667 | 10.3798 | 10.9535 | 10.60 | 10.80 |
| D047 | 10 | 11.5667 | .20817 | .12019 | 11.0496 | 12.0838 | 11.40 | 11.80 |
| Total | 2660 | 9.5567 | .40079 | .23140 | 8.5610 | 10.5523 | 9.22 | 10.00 |

## Table S2 The different rapid viscosity analyser profiles of maize starch

| | Peak | Trough | Breakdown | Final Visc | Setback | Peak Time | past Temp |
|---|---|---|---|---|---|---|---|
| CIMBL12 | 2154 | 1549 | 605 | **2732** | 1183 | 4.98 | 67.7 |
| zheng58 | 2053 | 1425 | 628 | **2734** | 1309 | 4.85 | 79.15 |
| CIMBL153 | 2033 | 1456 | 577 | **3172** | 1716 | 5.05 | 79.85 |
| CIMBL140 | 2577 | 1582 | 995 | **3220** | 1638 | 4.58 | 66.1 |

## Table S3 SNPs significantly associated ($P < 0.0001$) with starch granules size

| | SNP | Chromosome | Position | P.value |
|---|---|---|---|---|
| SGL (starch granule length) | SNP30343 | 7 | 75853279 | 0.00000363 |
| | SNP30436 | 7 | 81035068 | 0.00000851 |
| | SNP30423 | 7 | 80493311 | 0.00000973 |
| | SNP30425 | 7 | 80573170 | 0.0000123 |
| | SNP30418 | 7 | 80421800 | 0.0000228 |
| | SNP28873 | 6 | 155043436 | 0.0000303 |
| | SNP17473 | 3 | 225699972 | 0.0000335 |
| | SNP30419 | 7 | 80421839 | 0.0000396 |
| | SNP43662 | 7 | 80745735 | 0.0000435 |
| | SNP30432 | 7 | 80782295 | 0.0000499 |
| | SNP30438 | 7 | 81035283 | 0.0000514 |
| | SNP30437 | 7 | 81035113 | 0.0000665 |
| | SNP1148 | 3 | 202681272 | 0.000088 |
| | SNP30430 | 7 | 80748880 | 0.0000969 |
| | SNP30343 | 7 | 75853279 | 0.0000333 |
| | SNP30423 | 7 | 80493311 | 0.0000336 |
| | SNP30425 | 7 | 80573170 | 0.0000389 |
| | SNP30436 | 7 | 81035068 | 0.0000546 |
| | SNP30418 | 7 | 80421800 | 0.0000604 |
| | SNP17473 | 3 | 225699972 | 0.0000881 |
| | SNP28873 | 6 | 155043436 | 0.0000895 |
| | SNP22889 | 5 | 40079301 | 0.0000908 |
| | SNP22884 | 5 | 39934864 | 0.0000972 |

Table S4 Gene ontology predictions for selected genes.

| Gene ID | Gene identifier in Phytozome database | Seq. Description | Seq. Length (bp) | #Hits | Min. eValue | #GOs | GOs |
|---|---|---|---|---|---|---|---|
| GRMZM2G167673 | Zea mays 6a | cytochrome p450 714d1-like | 1381 | 11 | 9.40E-47 | 14 | P:gibberellin catabolic process; P:electron transport chain; C:mitochondrion; C:Integral to membrane; F:oxidoreductase activity; P:secretion by cell; F:electron carrier activity; F:monooxygenase activity; C: cytoplasmic membrane-bounded vesicle; F:heme binding; P:unidimensional cell growth; P:multicellular organismal development; C:multicellular organismal development; F:iron ion binding |
| GRMZM2G419655 | Zea mays 6a | phytosulfokine receptor precursor | 775 | 7 | 4.50E-54 | 3 | F:protein kinase activity; F:nucleotide binding; C:membrane |
| GRMZM2G419660 | Zea mays 6a | phytosulfokine receptor precursor | 1071 | 8 | 6.30E-54 | 7 | P:protein phosphorylation; F: protein serine/threonine kinase activity; C:integral to membrane; P:response to wounding; C:cytoplasmic membrane-bounded vesicle; F:ATP binding; F;peptide receptor activity |
| GRMZM2G511067 | Zea mays 6a | 52 kda repressor of the inhibitor of the protein kinase-like | 426 | 10 | 7.90E-105 | 2 | F:nucleic acid binding; F:protein dimerization activity |
| GRMZM6G663759 | Zea mays 6a | zinc finger ccch domain-containing protein 37 isoform x1 | 1349 | 2 | 0.00E+00 | 22 | F:metal ion binding |

| | | | | | |
|---|---|---|---|---|---|
| GRMZM2G180104 | Zea mays 6a | none | 2726 | 11 | 1.40E-85 |

# S5 The sequence of GRMZM2G419655

# Bigger starch granules size

Zhong69

CGGCTTTTGTACGTCTCTCGATCATGCAACTTGACTTGTCCTATAACAAGTTCACAGGCTCCATCCC
TGATGTCTTTGGAGAGATGAGGAGGCTAGAGTCCGTAAACCTGGCCACCAATAGGCTGGATGGTG
AGTTGCCCGCTTCCCTGTAAAGTTCTCCACTGCTGAGGGTAATCACCTTGAGGAACAACTCGCTCT
CTAGTGAGATTGCTATCGACTCCAGCTTGCTGCCGAAGCTTAATACTTTTGATGTTGGAACCAACT
ATCTGAGTGGCGCTATAGCTACTGGCATCAGTGTGTGCACCAAGTTGAGGACACTGAATCTTACAA
GGAACAAGCTCGTGGGGGAGATACCAGAGAGCTTCAAGGAGTTGAGATCCGGGCCAACCCTCCCC
TAACCAGGAAAAATTTCCCAACCGGGCAAAAAAATTGGAGGGTTTGGAAACCCTGCCCAACCTGA
AAAGCTTGGGGGTAACCAGGAATTTCCCGAGGGGGAAAAACATGGAGGGGGCCATCTTGGCAAA
CTGGTTACTCCTCGGCGTAATTCCCCCCGGGCTGCCAAACTTGGGAAGCCCCCAGTGCGTGGAAAT
TTCAGGGAACCAGTTTATAGGGAATTACCGCCCTGGGAGGGGGAAACCTAGACACTGCCTCAACA
TTGACTTCTCAAACATTCTTCCACCGTAACCTACTGTGTCGCTTCCACACAATGAGGAGCTGGAATT
CAAATATGTGGAGCCAACCAATTA

CML121

GGGGCATGGGTACTCTCTCGATCATGCAACTTGACTTGTCCTATAACAAGTTCACAGGCTCCATCC
CTGATGTCTTTGGAGAGATGAGGAGGCTAGAGTCCGTAAACCTGGCCACCAATAGGCTGGATGGT
GAGTTGCCCGCTTCCCTGTAAAGTTCTCCACTGCTGAGGGTAATCAGCTTGAGGAACAACTCGCTC
TCTGGTGAGATTGCTATCGACTCCAGCTTGCTGCCGAAGCTTAATACTTTTGATGTTGGAACCAACT
ATCTGAGTGGCGCTATAGCTCCTGGCATCAGTGTGTGCACCAAGTTGAGGACACTGAATCTTACAA
GGAACAAGCTCGTGGGGGAAACCCGAAAGCTTTCAGGGGTTTAAATCCCCGGCCTAACCCTCCCTT
AACCCGAAAAATTTCCCAACCCGGGATAAAAATTTGAAGGCTTGGGAAACCCGGCCCAACCGAAA
AACTTGGGGCTGAACCCGAAATTTCCCGGGGGGGAAACAATGGGAGTGCTCCTCCCGACAAAATA
TCTACTCCTCCGCGTAAATTCCCCCTGGGTGCCCAAATTGGGAAACCCCCATGTGCTGGAAAATTT
CTGTAACAAATTTAATGGGAATATCCCGCCCTGGGTGGGGAAACTAGACAACTGCTTCTACATTTA
TCTCTCAAACAATTCTTTCTACGCGAAACTACCTGTTAGCTTCACACACATGAGGAGCTTTGAATCC
AATAAAGGGTCGGGGCAAACGAAAAA

CIMBL87

ACGGCCTTGGGGTAATCTCTCGATCATGCAACTTGACTTGTCCTATAACAAGTTCACAGGCTCCAT
CCCTGATGTCTTTGGAGAGATGAGGAGGCTAGAGTCCGTAAACCTGGCCACCAATAGGCTGGATG
GTGAGTTGCCCGCTTCCCTGTAAAGTTCTCCACTGCTGAGGGTAATCAGCTTGAGGAACAACTCGC
TCTCTGGTGAGATTGCTATCGACTCCAGCTTGCTGCCGAAGCTTAATACTTTTGATGTTGGAACCAA
CTATCTGAGTGGCGCTATAGCTCCTGGCATCAGTGTGTGCACCAAGTTGAGGACACTGAATCTTAC
AAGGAACAAGCTCGTGGGGGAGAATCCAGAAGATCTCAGAGGTTAAAAACCCGGTGTCACCCCTC
CCGAACACGAAAAATTTTTCCACCCCGGGTAAAAAATTTTGGGGGTTTGGCACCCCCCCCCCCCCC
AGAAAATTGTGGTGATCACGAAATTTTCCCGCGGGGGAAAAACAGTGGGGGTCCTCCTCATAAAA
AATTGTCTCCTCCTCGGAATTTTCCCCCGGGGGCTAAAGATGTGGAAACCCCTCGTGGTGGTGAAT
TTTTGTTGAAACATTTAAGAGGAAAATCTCCCGCGGGGGGTGGAGAATCAAAACAGCTTCTTCAAT
TTTCTCTTCTAAAATTTTTTTTTCGCGGAAATACCTCTTGTCTTCTCCACAAAGATGAGGATGTAGT
TTTAAAATGGGCCTCGAAAAACAAAAA

CIMBL10

TCGGCAGTGACCTTGGTAACCTCTCTCAGATCATGCAACTTGACTTGTCCTATAACAAGTTCACAG
GCTCCATCCCTGATTCCTTTGGAGAGATGAAGAGGCTAGAGTCCGTAAACCTGGCCACCAATAGGC
TGGATGGTGAGTTGCCCGCTTCCCTGTAAAGTTGTCCACTGCTGAGGGTAATCAGCTTGAGGAACG
ACTCGCTCTCTGGTGAGATTGCTATCGACTCCAGCTTGCTGCCGAAGCTTAATACTTTTGATGTTGG
AACCAACAATCTGAGTGGCGCTATAGCTCCTGGCATCAGTGTGTGCACCAAGTTGAGGACACTGA
ATCTTACAAGGAACAAGCTCGTGGGGGATACCGGAGAGCTTCAAGGAGTTGAGATCCCTGTCGTA
CCTCTCGCTGACCAGGAATAGTTTCACAACCTGGCATAGACATTGCAGGTCTTGCAACACCTGCCC
AACCTGACAAGCTTGGTGCTGACCAGGAATTTCCGCGGTGGTGAGACAATGCAGGTGCTCATCCTG
ACAAACTATCTACTCATCGGCGTAATTCCCCCGTGGCTGCAGAGCTTGGGGAGCCTCAATGTGCTG
GAAATTTCATGTAACAAGTTAGATGGGAATATCCTGCCATGGCTGGGGAAGCTAGACAACTGCTTC
AACATTGATCTCTCAAACAATTCTTTCAGCGGTAAGCTACCTGTTAGCTTCACACAGATGAGGAGC
TTGAATTCAA

CIMBL84

TCGGCAGTGACCTTGGTAACCTCTCTCAGATCATGCAACTTGACTTGTCCTATACCAAGTTCACAG
GCTCCATCCCTGATGTCTTTGGAGAGATGAGGAGGCTAGAGTCTGTAAGCCTGGCCACCAATAGGC
TGGATGGTGAGTTGTCCGCTTCCCTGTAAAGTTCTCCACTGCTGAGGGTAATCAGCTTGAGGAACA
ACTCGCTCTCTGGTGAGATTGCTATCGACTCCAGCTTGTTGCCGAAGCTTAATACTTTTGATGTTGG

AACCAACTATCCGAGTGGCGCTATAGCTACTGGCATCAGTGTGTGCACCAAGTTGAGGACACTGA
ATTTTACAAGGAACAAGCTCGTGGGGGGATACCGGAGAGCTTCAAGGAGTTGAGATCCCTGTCGT
ACCTCTCGCTGACCAGGAATAGCTTCACAACCTAGCATAGACATTGCAGGTCTTGCAACACCTGCT
CAACCTGACAAGCTTGGTGCTGACCAGGAATTTCCACGGCGGTGAGACAATGCAGGTGCTCATCCT
GACAAATTGTCTACTCATCGGCGTAATTCCCCCGTGGCTGCAGAGCTTGGGAAGCCTCAATGTGCT
GGAAATTTCATGTAACAAGTTAGATGGGAATATCCCGCCATGGCTGGGGAAGCTAGACAACTGCT
TCAACATTGACCTCTCAAACAATTCTTTCAGCGGTAAGCTATCTGTCAGCTTCACACAGATGAGGA
GCTTGAATTCAA

Nan21-3

TCGGCAGTGACCTTGGTAACCTCTCTCAGATCATGCAACTTGACTTGTCCTATAATAAGTTCACAG
GCTCCATACTTGATGTCTTTGGAGAGATGAGGAGGCTAGAGTCCGTAAACCTGGCCACCAATAGG
CTGGATGGTGAGTTGCCCGATTCCCTGTAAAGTTGTCCACTTCTGAGGGTAATCAGCTTGAGGAAC
AACTCGCTCTCTGGTGAGATTGCTATCGACTCCAGCTTGCTGCCGAAGCTTAATACTTTTGATGTTG
GAACCAACTATCTGAGTGGTGCTATAGCTCCTGGCATCAGTGTGTGCACCAAGTTGAGGACACTAA
ATCTTACAAGGAACAAGCTCGTGGGGGATACCGGAGAGCTTCAAGGAGTTGAGATCCCTGTCGTA
CCTCTCGCTGACCAGGAATAGTTTCACAACCTGGCATAGACATTGCAGGTCTTGCAACACCTGCCC
AACCTGACAAGCTTGGTGCTGACCAGGAATTTCCGCGGTGGTGAGACAATGCAGGTGCTCATCCTG
ACAAACTATCTACTCATCGGCGTAATTCCCCCGTGGCTGCAGAGCTTGGGAAGCCTCAATGTGCTG
GAAATTTCATGTAACAAGTTAGATGGGAATATCCTGCCATGGCTGGGGAAGCTAGACAACTGCTTC
AACATTGATCTCTCAAACAATTCTTTCAGCGGTAAGCTACCTGTTAGCTTCACACAGATGAGGAGC
TTGAATTCAA

Xun971

TCGGCAGTGACCTTGGTAACCTCTCTCAGATCATGCAACTTGACTTGTCCTATAACAAGTTCACAG
GCTCCATCCCTGATGTCTTTGGAGAGATGAGGAGGCTAGAGTCCGTAAACCTGGCCACCAATAGG
CTGGACGGTGAGTTGCCCGCTTCCCTGTAAAGTTGTCCACTGCTGAGGGTAATCAGCTTGAGGAAC
TACTCGCTCTCTGGTGAGATTGCTATCGACTCCAGCTTGCTGCCGAAGCTTAATACTTTTGATGTTG
GAACCAACTATCTGAGTGGCGCTATAGCTCCTGGCATCAGTGTGTGCACCAAGTTGAGGACACTGA
ATCTTACAAGGAACAAGCTCGTGGGGGGGGATACCGGAGAGCTTCAAGGAGTTGAGATCCCTGTC
GTACCTCTCGCTGACCAGGAATAGTTTCACAACCTGGCATAGACATTGCAGGTCTTGCAACACCTG
CCCAACCTGACAAGCTTGGTGCTGACCAAGAATTTCCGCGGCGGTGAGACAATGCAGGTGCTCAT
CCTGACAAACTGTCTACTCATCGGCGTAATTCCCCCGTGGCTGCAGAGCTTGGGAAGCCTCAATGT
GCTGGAAATTTCATGTAACAAGTTAGATGGGAATATCCCGCCATGGCTGGGGAAGCTAGACAACT
GCTTCAACATTGACCTCTCAAACAATTCTTTCAGCGGTAAGCTACCTGTCAGCTTCACACAGATGA
GGAGCTTGAATTCAA

M153

TCGGCAGTGACCTTGGTAACCTCTCTCAGATCATGCAACTTGACTTGTCCTATACCAAGTTCACAG
GCTCCATCCCTGATGTCTTTGGAGAGATGAGGAGGCTAGAGTCTGTAAACCTGGCCACCAATAGGC
TGGATGGTGAGTTGCCCGCTTCCCTGTAAAGTTCTCCACTGCTGAGGGTAATCAGCTTGAGGAACC
ACTCGCTCTCTGGTGAGATTGCTATCGACTCCAGCTTGCTGCCGAAGCTTAATACTTTTGATGTTGG
AACCAACTATCCGAGTGGCGCTATAGCTACTGGCATCAGTGTGTGCACCAAGTTGAGGACACTGA
ATTTTACAAGGAACAAGCTCGTGGGGGGATACCGGAGAGCTTCAAGGAGTTGAGATCCCTGTCGT
ACCTCTCGCTGACCAGGAATAGTTTCACAACCTGGCATAGACATTGCAGGTCTTGCAACACCTGCC
CAACCTGACAAGCTTGGTGCTGACCAGGAATTTCCGCGGCGGTGAGACAATGTAGGTGCTCATCCT
GACAAACTGTCTACTCATCGGCGTAATTCCCCCGTGGCTGCAGAGCTTGGGAAGCCTCAATGTGCT
GGAAATTTCATGTAACAAGTTAGATGGGAATATCCCGCCATGGCTGGGGAAGCTAGACAACTGCT
TCAACATTGACCTCTCAAACAATTCTTTCAGCCGTAAGCTACTTGTCAGCTTCACACAGATGAGGA
GCTTGAATTCAA

CIMBL91

TGAGGCATGGTACTCTCTAGATCATGCAACTTGACTTGTCCTATACCAAGTTCACAGGCTCCATCC
CTGATGTCTTTGGAGAGATGAGGAGGCTAGAGTCCGTAAACCTGGCCACCAATAGGCTGGATGGT
GAGTTGCCCGCTTCCCTGTAAAGTTCTCCACTTCTGAGGGTAATCAGCTTGAGGAACAACTCGCTC
TCTGGTGAGATTGCTATCGACTCCAGCTTGCTGCCGAAGCTTAATACTTTTGATGTTGGAACCAACT
ATCTGAGTGGCGCTATAGCTACTGGCATCAGTGTGTGCACCAAGTTGAGGACACTGAATCTTACAA
GGAACAAGCTCCTGGGGGAAAACCGAAAAATTTCCAGGGATTAAAAACCCGTGCCAACCCTCCCT
TAACCCGGAAAAAATTTCCAAACCGGGATAAAAATTTTGAGGGTTTGGACACCCGGCCCAACCGGG
AAAATTTGGGGGTAACCGGAAATTTCCCGGGGGGGAGAGACAGGGGGGGGCGCTCTCCGAGAAAA
TGTGCAAACTTCTGGGTAAATTCCCCCCGGCCGCGGAGATTGGGGAACCTCTATGTGCTTGAAACT
TTTATGTAACATTAAAAGGAAAAACCGCCCTGGGCTGGAAAGCTAGAACTGCTTCACACTTTGCTC
TCTCACACATCTCTCCACGGGTAAGCTCTGTGAAGCTTCACACATTAAGGATTTGATTCATAAAGG
T

Ye8001
GGGCATTGTCTCTCTAGATCATGCAACTTGACTTGTCCTATAACAAGTTCACAGGCTCCATCCCTGA
TGTCTTTGGAGAGATGAGGAGGCTAGAGTCCGTAAACCTGGCCACCAATAGGCTGGATGGTGAGT
TGCCCGCTTCCCTGTAAAGTTCTCCACTGCTGAGGGTAATCAGCTTGAGGAACAACTCGCTCTCTG
GTGAGATTGCTATCGACTCCAGCTTGCTGCCGAAGCTTAATACTTTTGATGTTGGAACCAACTATCT
GAGTGGCGCTATAGCTCCTGGCATCAGTGTGTGCACCAAGTTGAGGACACTGAATCTTACAAGGA
ACAAGCTCGTGGGGGGGGATACCAGAGATCTTCAGGGATTAGATCCCCGTGTCACCCCTCTCGGA
GACCAAAAATATTTTCACCCCGGGGTATACATTTTGGGGGTTTTGCAACCCCTGCCAAACGTGAAA
ATTGTGGTGCAGACCAAAATTTCCCGCGGGGGAGACACAGTGCGGGTGCTCCTCGTGAAAAAGTG
TCTTCTCATCGGTATATTTCCCCCGTGGCTGCAGATCGTGGGACGCCTCAGTGTGCTAGATTTTTCG
TGTAACGTGTTATATGAGAATATCGCGCTGTGTGTGGAAATCTACACATCTGCTACATTTGATCTCT
CACACTTCTCTCTCAGGAAATCTACGTGTCATCTTCACACATAAGAAGATTTGTTTCATATATGTCG
CML470
GGGCAATTGATCTCTCTAGATCATGCAACTTGACTTGTCCTATAACAAGTTCACAGGCTCCATCCCT
GATGTCTTTGGAGAGATGAGGAGGCTAGAGTCCGTAAACCTGGCCACCAATAGGCTGGATGGTGA
GTTGCCCGCTTCCCTGTAAAGTTCTCCACTGCTGAGGGTAATCAGCTTGAGGAACAACTCGCTCTC
TGGTGAGATTGCTATCGACTCCAGCTTGCTGCCGAAGCTTAATACTTTTGATGTTGGAACCAACTA
TCTGAGTGGCGCTATAGCTCCTGGCATCAGTGTGTGCACCAAGTTGAGGACACTGAATCTTACAAG
GAACAAGCTCGTGGGGGAGAATCCAGAAGATCTTCGAGGTGAAAACCCCGGTGTCACCCCCCCCG
AACACGAAAAATTTTTCCACCCGGGGGAAAAAATTTTGGGGGTTTTGCAACCCCCCCCCCCCGTGA
AATTTGTGGTGAACACGAAATTTTCCCGCGGGGGAAAAAAGGTGGGGGTGCTCCTCGTAAAAAAT
TGTCTGCTCAGCGGAATTTTCCCCCGGGGGCTAAAGATGGTGAGACCCCACGTTGTGGGGAATTTT
TGGTGAAACATTAAAGGGGATAATCTCCCCCCGTGGGGGGAAATTCTACACTGCTGCTACAATGTT
CACCTCTAAAACTCTTTTTTCGCCAAAAACCACCTGTCATCTCCCCAAAATGAGGATCTAGTTTTCT
AATGGGCTCCGAAACCCAAAA
Zheng29
AATCAGTTCACTGGCAACCTCGGCAGTGACCTTGGTAACCTCTCTCAGATCATGCAACTTGACTTG
TCCTATACCAAGTTCACAGGCTCCATCCCTGATGTCTTTGGAGAGATGAGGAGGCTAGAGTCTGTA
AACCTGGCCACCAATAGGCTGGATGGTGAGTTGCCCGCTTCCCTGTAAAGTTCTCCACTGCTGAGG
GTAATCAGCTTGAGGAACAACTCGCTCTCTGGTGAGATTGCTATCGACTCCAGCTTGTTGCCGAAG
CTTAATACTTTTGATGTTGGAACCAACTATCCGAGTGGCGCTATAGCTACTGGCATCAGTGTGTGC
ACCAAGTTGAGGACACTGAATTTTACAAGGAACAAGCTCGTGGGGGGATACCGGAGAGCTTCAAG
GAGTTGAGATCCCTGTCGTACCTCTCGCTGACCAGGAATAGTTTCACAACCTGGCATAGACATTGC
AGGTCTTGCAACACCTGCCCAACCTGACAAGCTTGGTGCTGACCAGGAATTTCCGCGGCGGTGAG
ACAATGTAGGTGCTCATCCTGACAAACTGTCTACTCATCGGCGTAATTCCCCCGTGGCTGCAGAGG
TTGGGAAGCCTCAATGTGCTGGAAATTTCATGTAACAAGTTAGATGGGAATATCCTGCCATGGCTG
GGGAAGCTAGACAACTGCTTCAACATTGATCTCTCAAACAATTCTTTCAGCGGTAAGCTACCTGTT
AGCTTCACACAGATGAGGAGCTTGAATTCAAATAATGGCTCGAGCAAGCGA
CML325
AATCAGTTCACTGGCAACCTCGGCAGTGACCTTGGTAACCTCTCTCAGATCATGCAACTTGACTTG
TCCTATACCAAGTTCACAGGCTCCATCCCTGATGTCTTTGGAGAGATGAGGAGGCTAGAGTCTGTA
AACCTGGCCACCAATAGGCTGGATGGTGAGTTGCCCGCTTCCCTGTAAAGTTCTCCACTGCTGAGG
GTAATCAGCTTGAGGAACAACTCGCTCTCTGGTGAGATTGCTATCGACTCCAGCTTGTTGCCGAAG
CTTAATACTTTTGATGTTGGAACCAACTATCCGAGTGGCGCTATAGCTACTGGCATCAGTGTGTGC
ACCAAGTTGAGGACACTGAATTTTACGAGGAACAAGCTCGTGGGGGGATACCGGAGAGCTTCAAG
GAGTTGAGATCCCTGTCGTACCTCTCGCTGACCAGGAATAGTTTCACAACCTGGCATAGACATTGC
AGGTCTTGCAACACCTGCCCAACCTGACAAGCTTGGTGCTGACCAGGAATTTCCGCGGCGGTGAG
ACAATGTAGGTGCTCATCCTGACAAACTGTCTACTCATCAGCGTAATTCCCCCGTGGCTGCAGAGC
TTGGGAAGCCTCAATGTGCTGGAAATTTCATGTAACAAGTTAGATGGGAATATCCCGCCATGGCTG
GGGAAGCTAGACAACTGCTTCAACATTGACCTCTCAAACAATTCTTTCAGCCGTAAGCTACTTGTC
AGCTTCACACAGATGAGGAGCTTGAATTCAAATAATGGCTCGAGCAAGCGA

FCD0602
TCGGCAGTGACCTTGGTAACCTCTCTCAGATCATGCAACTTGACTTGTCCTATACCAAGTTCACAG
GCTCCATCCCTGATGTCTTTGGAGAGATGAGGAGGCTAGAGTCTGTAAACCTGGCCACCAATAGGC
TGGATGGTGAGTTGCCCGCTTCCCTGTAAAGTTCTCCACTCCTGAGGGTAATCAGCTTGAGGAACA
ACTCGCTCTCTGGTGAGATTGCTATCGACTCCAGCTTGTTGCCGAAGCTTAATACTTTTGATGTTGG
AACCAACTATCCGAGTGGCGCTATAGCTACTGGCATCAGTGTGTGCACCAAGTTGAGGACACTGA
ATTTTACAAGGAACAAGCTCGTGGGGGGATACCAGAGAGCTTCAAGGAGTTGAGATCCCTGTCGT
ACCTCTCGCTGACCAGGAATAGTTTCACAACCTGGCATAGACATTGCAGGTCTTGCAACACCTGCC

CAACCTGACAAGCTTGGTGCTGACCAGGAATTTCCGCGGCGGTGAGACAATGTAGGTGCTCATCCT
GACAAACTGTCTACTCATCGGCGTAATTCCCCCGTGGCTGCAGAGCTTGGGAAGCCTCAATGTGCT
GGAAATTTCATGTAACAAGTTAGATGGGAATATCCCGCCATGGCTGGGGAAGCTAGACAACTGCT
TCAACATTGACCTCTCAAACAATTCTTTCAGCCGTAAGCTACTTGTCAGCTTCACACAGATGAGGA
GCTTGAATTCAA

04K5702

TCGGCAGTGACCTTGGTAACCTCTCTCAGATCATGCAACTTGACTTGTCCTATAACAAGTTCACAG
GCTCCATCCCTGATGTCTTTGGAGAGATGAGGAGGCTAGAGTCCGTAAACCTGGCCACCAATAGG
CTGGATGGTGAGTTGCCCGCTTCCCTGTAAAGTTCTCCACTGCTGAGGGTAATCAGCTTGAGGAAC
AACTCGCTCTCTAGTGAGATAGCTATCGACTCCAGCTAGCTGCCGAAGCTTAATACTTTTGATGTT
GGAACCAACTATCTGAGTGGTGCTATAGCTCCTGGCATCAGTGTGTGCACCAAGTTGAGGACACTA
AATCTTACAAGGAACAAGCTCGTGGGGGATACCGGAGAGCTTCAAGGAGTTGAGATCCCTGTCGT
ACCTCTCGCTGACCAGGAATAGTTTCACAACCTGGCATAGACATTGCAGGGTCTTGCAACACGCTG
CCCAACCTGACAAGCTTGGTGCTGACCAGGAATTTCCGCGGTGGTGAGACAATGCAGGTGCTCATC
CTGACAAACTATCTACTCATCGGCGTAATTCCCCCGTGGCTGCAGAGCTTGGGAAGCCTCAATGTG
CTGGAAATTTCATGTAACAAGTTAGATGGGAATATCCTGCCATGGCTGGGGAAGCTAGACAACTG
CTTCAACATTGATCTCTCAAACAATTCTTTCAGCGGTAAGCTACCTGTTAGCTTCACACAGATGAG
GAGCTTGAATTCAA

GEMS61

TCGGCAGTGACCTTGGTAACCTCTCTCAGATCATGCAACTTGACTTGTCCTATAACAAGTTCACAG
GCTCCATCCCTGATGTCTTTGGAGAGATGAGGAGGCTAGAGTCCGTAAACCTGGCCACCAATAGG
CTGGATGGTGAGTTGCCCGCTTCCCTGTAAAGTTCTCCACTGCTGAGGGTAATCAGCTTGAGGAAC
AACTCGCTCTCTAGTGAGATTGCTATCGACTCCAGCTTGCTGCCGAAGCTTAATACTTTTGATGTTG
GAACCAACTATCTGAGTGGTGCTATAGCTCCTGGCATCAGTGTGTGCACCAAGTTGAGGACACTAA
ATCTTACAAGGAACAAGCTCGTGGGGGATACCGGAGAGCTTCAAGGAGTTGAGATCCCTGTCGTA
CCTCTCGCTGACCAGGAATAGTTTCACAACCTGGCATAGACATTGCAGGTCTTGCAACACCTGCCC
AACCTGACAAGCTTGGTGCTGACCAGGAATTTCCGCGGTGGTGAGACAATGCAGGTGCTCATCCTG
ACAAACTATCTACTCATCGGCGTAATTCCCCCGTGGCTGCAGAGCTTGGGAAGCCTCAATGTGCTG
GAAATTTCATGTAACAAGTTAGATGGGAATATCCTGCCATGGCTGGGGAAGCTAGACAACTGCTTC
AACATTGATCTCTCAAACAATTCTTTCAGCGGTAAGCTACCTGTTAGCTTCACACAGATGAGGAGC
TTGAATTCAA

835b

TCGGCAGTGACCTTGGTAACCTCTCTCAGATCATGCAACTTGACTTGTCCTATAACAAGTTCACAG
GCTCCATCCCTGATGTCTTTGGAGAGATGAGGAGGCTAGAGTCCGTAAACCTGGCCACCAATAGG
CTGGACGGTGAGTTGCCCGCTTCCCTGTAAAGTTGTCCACTGCTGAGGGTAATCAGCTTGAGGAAC
TACTCGCTCTCTGGTGAGATTGCTATCGACTCCAGCTTGCTGCCGAAGCTTAATACTTTTGATGTTG
GAACCAACTATCTGAGTGGCGCTATAGCTCCTGGCATCAGTGTGTGCACCAAGTTGAGGACACTGA
ATCTTACAAGGAACAAGCTCGTGGGGGGGGATACCGGAGAGCTTCAAGGAGTTGAGATCCCTGTC
GTACCTCTCGCTGACCAGGAATAGTTTCACAACCTGGCATAGACATTGCAGGTCTTGCAACACCTG
CCCAACCTGACAAGCTTGGTGCTGACCAAGAATTTCCGCGGCGGTGAGACAATGCAGGTGCTCAT
CCTGACAAACTGTCTACTCATCGGCGTAATTCCCCCGTGGCTGCAGAGCTTGGGAAGCCTCAATGT
GCTGGAAATTTCATGTAACAAGTTAGATGGGAATATCCCGCCATGGCTGGGGAAGCTAGACAACT
GCTTCAACATTGACCTCTCAAACAATTCTTTCAGCGGTAAGCTACCTGTCAGCTTCACACAGATGA
GGAGCTTGAATTCAA

CIMBL54

AATCAGTTCACTGGCAACCTCGGCAGTGACCTTGGTAACCTCTCTCAGATCATGCAACTTGACTTG
TCCTATAATAAGTTCACAGGCTCCATACTTGATGTCTTTGGAGAGATGAGGAGGCTAGAGTCCGTA
AACCTGGCCACCAATAGGCTGGATGGTGAGTTGCCCGATTCCCTGTAAAGTTGTCCACTTCTGAGG
GTAATCAGCTTGAGGAACAACTCGCTCTCTGGTGAGATTGCTATCGACTCCAGCTTGCTGCCGAAG
CTTAATACTTTTGATGTTGGAACCAACTATCTGAGTGGCGCTATAGCTTCTAGCATCAGTGTGTGCA
CCAAGTTGAGGACACTGAATCTTACAAGGAACAAGCTCATGGGGGAGATACCAGAGAGCTTCAAG
GAGTTGAGATCCGTGTCGTACCTCTCGCTGACCAGGAATAGCTTCACAACCTGGCATAGACATTGC
AGGTCTTGCAACACCTGCCCAACCTGACAAGCTTGGTGCTGACCAGGAATTTCCGCGGTGGTGAGA
CAATGCAGGTGCTCATCCTGACAAACTGTCTACTCATCGGCGTAATTCCCCCGTGGCTGCAGAGGT
TGGGAAGCCTCAATGTGCTGGAAATTTCATGTAACAAGTTAGATGGGAATATCCCGCCATGGCTGG
GGAAGCTAGACAACCGCTTCAACATTGACCTCTCAAACAATTCTTTCAGCGGTAAGCTACCTGTCA
GCTTCACACAGATGAGGAGCTTGAATTCAAATAATGGCTCGAGCAAGCGA

ZaC546

AATCAGTTCACTGGCAACCTCGGCAGTGACCTTGGTAACCTCTCTCAGATCATGCAACTTGACTTG
TCCTATAATAAGTTCACAGGCTCCATACTTGATGTCTATGGAGAGATGAGGAGGCTAGAGTCCGTA

AACCTGGCCACCAATAGGCTGGATGGTGAGTTGCCCGATTCCCTGTAAAGTTGTCCACTTCTGAGG
GTAATCAGCTTGAGGAACAACTCGCTCTCTGGTGAGATTGCTATCGACTCCAGCTTGCTGCCGAAG
CTTAATACTTTTGATGTTGGAACCAACTATCTGAGTGGCGCTATAGCTTCTAGCATCAGTGTGTGCA
CCAAGTTGAGGACACTGAATCTTACAAGGAACAAGCTCATGGGGGAGATACCAGAGAGCTTCAAG
GAGTTGAGATCCGTGTCGTACCTCTCGCTGACCAGGAATAGCTTCACAACCTGGCATAGACATTGC
AGGTCTTGCAACACCTGCCCAACCTGACAAGCTTGGTGCTGACCAGGAATTTCCGCGGTGGTGAGA
CAATGCAGGTGCTCATCCTGACAAACTGTCTACTCATCGGCGTAATTCCCCCGTGGCTGCAGAGGT
TGGGAAGCCTCAATGTGCTGGAAATTTCATGTAACAAGTTAGATGGGAATATCCCGCCATGGCTGG
GGAAGCTAGACAACCGCTTCAACATTGACCTCTCAAACAATTCTTTCAGCGGTAAGCTACCTGTCA
GCTTCACACAGATGAGGAGCTTGAATTCAAATAATGGCTCGAGCAAGCGA

3H-2

AATCAGTTCACTGGCAACCTCGGCAGTGACCTTGGTAACCTCTCTCAGATCATGCAACTTGACTTG
TCCTATAACAAGTTCACAGGCTCCATCCCTGATGTCTTTGGAGAGATGAGGAGGCTAGAGTCCGTA
AACCTGGCCACCAATATGCTGGATGGTGAGTTGCCCGCTTCCCTGTAAAGTTGTCCACTGCTGAGG
GTAATCAGCTTGAGGAACAACTCGCTCTCTGGTGAGATTGCTATCGACTCCAGCTTGCTGCCGAAG
CTTAATACTTTTGATGTTGGAACCAACAATCTGAGTGGCGCTATAGCTCCTGGCATCAGTGTGTGC
ACTAAGTTGAGGACACTGAATCTTACAAGGAACAAGCTCGTGGGGGAGATACTAGAGAGCTTCAA
GGAGTTGAGATCTCTGTCGTACCTCTCGCTGACCAGGAATAGCTTCACAACCTAGCATAGACATTG
CAGGTCTTGCAACACCTGCTCAACCTGACAAGCTTGGTGCTGACCAGGAATTTCCACGGCGGTGAG
ACAATGCAGGTGCTCATCCTGACAAACTGTCTACTCATCGGCGTAATTCCCCCGTGGCTGTAGAGC
TTGGGAAGCCTCAATGTGCTGGAAATTTCATGTAACAAGTTAGATGGGAATATCCCGCCATGGCTG
GGGAAGCTAGACAAACGCTTCAACATTGACCTCTCAAACAATTCTTTCAGCGGTAAGCTACCTGTC
AGCTTCACACAGCTGAGGAGCTTGAATTCAAATAATGGCTCGAGCAAGCGA

CIMBL88

TCGGCAGTGACCTTGGTAACCTCTCTCAGATCATGCAACTTGACTTGTCCTATAACAAGTTCACAG
GCTCCATCCCTGATGTCTTTGGAGAGATGAGGAGGCTAGAGTCCGTAAACCTGGCCACCAATAGG
CTGGATGGTGAGTTGCCCGCTTCCCTGTAAAGTTCTCCACTGCTGAGGGTAATCACCTTGAGGAAC
AACTCGCTCTCTAGTGAGATTGCTATCGACTCCAGCTTGCTGCCGAAGCTTAATACTTTTGATGTTG
GAACCAACTATCCGAGTGGCGCTATAGCTACTGGCATCAGTGTGTGCACCAAGTTGAGGACACTG
AATCTTACAAGGAACAAGCTCGTTGGGGGGATACCGGAGAGCTTCAAGGAGTTGAGATCCTGTCG
TACCTTTCGCTGACCAGGAATAGTTTCACAACCTGGCATAGACATTGCAGGTCTTGCAACACCTGC
CCAACCTGACAAGCTTGGTGCTGACCAGGAATTTCTGCAGCGGTGAGACAATGCAGGTGCTCATCT
TGACAAACTGTCTACTCATCGGCGTAATTCCCCCGTGGCTGCAGAGCTTGGGAAGCCTCAATGTGC
TGGAAATTTCATGTAACAAGTTAGATGGGAATATCCCGCCATGGATGGGGAGGCTAGACAACTGC
TTCAACATTGACCTCTCAAACAATTCTTTCAGCGGTAAGCTACTTGTCAGCTTCACACAGATGAGG
AGCTTGAATTCAA

CIMBL142

TCGGCAGTGACCTTGGTAACCTCTCTCAGATCATGCAACTTGACTTGTCCTATAATAAGTTCACAG
GCTCCATACTTGATGTCTTTGGAGAGATGAGGAGGCTAGAGTCCGTAAACCTGGCCACCAGTAGG
CTGGATGGTGAGTTGCCCGATTCCCTGTAAAGTTGTCCACTTCTGAGGGTAATCAGCTTGAGGAAC
AACTCGCTCTCTGGTGAGATTGCTAACGACTCCAGCTTGCTGCCGAAGCTTAATACTTTTGATGTTG
GAACCAACTATCTGAGTGGCGCTATAGCTTCTAGCATCAGTGTGTGCACCAAGTTGAGGACACTGA
ATCTTACAAGGAACAAGCTCATGGGGGAGATACCAGAGAGCTTCAAGGAGTTGAGATCCGTGTCG
TACCTCTCGCTGACCAGGAATAGCTTCACAACCTGGCATAGACATTGCAGGTCTTGCAACACCTGC
CCAACCTGGCAAGCTTGGTGCTGACCAGGAATTTCCGCGGTGGTGAGACAATGCAGGTGCTCATCC
TGACAAACTGTCTACTCATCGGCGTAATTCCCCCGTGGCTGCAGAGGTTGGGAAGCCTCAATGTGC
TGGAAATTTCATGTAACAAGTTAGATGGGAATATCCCGCCATGGCTGGGGAAGCTAGACAACCGC
TTCAACATTGACCTCTCAAACAATTCTTTCAGCGGTAAGCTACCTGTCAGCTTCACACAGATGAGG
AGCTTGAATTCAA

K22

TCGGCGGTGACCTTGGTAACCTCTCTCAGATCATGCAACTTGACTTGTCCTATAACAAGTTCACAG
GCTCCATCCCTGATGTCTTTGGAGAGATGAGGAGGCTAGAGTTCGTAAACCTGGCCACCAATAGGC
TGGATGGTGAGTTGCCCGCTTCCCTGTAAAGTTCTCCACTGCTGAGGGTAATCAGCTTGAGGAACA
ACTCGCTCTCTGGTGAGATTGCTATCGACTCCAGCTTGCTGCCGAAGCTTAATACTTTTGATGTTGG
AACCAACTATCTGAGTGGCGCTATAGCTCCTGGCATCAGTGTGTGCACCAAGTTGAGGACACTGAA
TCTTACAAGGAACAAGCTCGTGGGGGGGGATACCGGAGAGCTTCAAGGAGTTGAGATCCCTGTCG
TACCTCTCGCTGACCAGGAATAGTTTCACAACCTGGCATAGACATTGCAGGTCTTGCAACACCTGC
CCAACCTGACAAGCTTGGTGCTGACCAGGAATTTCCGCGGCGGTGAGACAATGCAGGTGCTCATC
CTAGCAAACTGTCTGCTCATCGGCGTAATTCCCCCGTGGCTACAGAGCTTGGGAAGCCTCAATGTG
CTGGAAATTTCATGTAACAAGTTAGATGGGAATATCCCGCCATGGCTAGGGAAGCTAGACAACTG

CTTCAACATTGACCTCTCAAACAATTCTTTCAGCGGTAAGCTACCTGTCAGCTTCACACAGATGAG
GAGCTTGAATTCAA
Dan360

AATCAGTTCACTGGCAACCTCGGCAGTGACCTTGGTAACCTCTCTCAGATCATGCAACTTGACTTG
TCCTATAACAAGTTCACAGGCTCCATCCCTGATGTCTTTGGAGAGATGAGGAGGCTAGAGTCCGTA
AACCTGGCCACCAATAGGCTGGATGGTGAGTTGCCCGCTTCCCTGTAAAGTTCTCCACTGCTGAGG
GTAATCAGCTTGAGGAACAACTCGCTCTCTAGTGAGATTGCTATCGACTCCAGCTTGCTGCCGAAG
CTTAATACTTTTGATGTTGGAACCAACTATCTGAGTGGTGCTATAGCTCCTGGCATCAGTGTGTGCA
CCAAGTTGAGGACACTAAATCTTACAAGGAACAAGCTCGTGGGGGATACCGGAGAGCTTCAAGGA
GTTGAGATCCCTGTCGTACCTCTCGCTGACCAGGAATAGTTTCACAACCTGGCATAGACATTGCAG
GTCTTGCAACACCTGCCCAACCTGACAAGCTTGGTGCTGACCAGGAATTTCCGCGGCGGTGAGACA
ATGCAGGTGCTCATCCTGACAAACTATCTACTCATCGGCGTAATTCCCCCGTGGCTGCAGAGCTTG
GGAAGCCTCAATGTGCTGGAAATTTCATGTAACAAGTTAGATGGGAATATCCTGCCATGGCTGGG
GAAGCTAGACAACTGCTTCAACATTGGTCTCTCAAACAATTCTTTCAGCGGTAAGCTACCTGTTAG
CTTCACACAGATGAGGAGCTTGAATTCAAATAATGGCTCGAGCAAGCGA

# Smaller starch granules size

B111
GGGGAATGGGAACTCTTCTCAAATCTGCAACTTGACTTGTCCTATAACAAGTTCACAGGCTCCATCC
CTGATGTCTTTGGAGAGATGAGGAGGCTAGAGTCCGTAAACCTGGCCACCAATAGGCTGGATGGTG
AGTTGCCCGCTTCCCTGTAAAGTTCTCCACTGCTGAGGGTAATCAGCTTGAGGAACAACTCGCTCTC
TAGTGAGATTGCTATCGACTCCAGCTTGCTGCCGAAGCTTAATACTTTTGATGTTGGAACCAACTAT
CTGAGTGGCGCTATAGCTCCTGGCATCAGTGTGTGCACCAAGTTGAGGACACTGAATCTTACAAGG
AACAAGCTCGTGGGGGAGATACCAGAGAGCTTCAAGGAGTTGAAATCCGGGGTAAACCTCCCCGA
AACCGAAAAATTTTTCAAAACCGGGAAAAAACTTTGGGGGTTTTGGCAACCCCGCCCAACCGGAAA
ATTTTGGGGGAAACCGAAAATTTCCCGCGGGGTGAAAACATGGAGGGTCTTCCTCAGAAAAAATGT
TCTCCTCTGCGGAAATTTCCCCCGGGGGTGAAAATTTTGGAAACCCCCAGTGGTGTGGAAATTTCGT
TGAAAAATTTAAAGGGAAATTCCCCCCCCGGGGGGGGAAAGCTAAAACATCTGTTACAATTTGCTCT
CCTAAAAATTTTTTTCCGGGGAAACCCCTCTGGCCTTCTCCACAAGAGGAGATTGTGATCTCAAAATA
GGGCCTGGAAAAGAAAA
Sy999
GGGCCAGGGGGTACTCTCTCAGATCATGCACTTGACTTGTCCTATAACAAGTTCACAGGCTCCATCCC
TGATGTCTTTGGAGAGATGAGGAGGCTAGAGTCCGTAAACCTGGCCACCAATAGGCTGGATGGTGAG
TTGCCCGCTTCCCTGTAAAGTTCTCCACTTCTGAGGGTAATCACCTTGAGGAACAACTCGCTCTCTAGT
GAGATTGCTATCGACTCCAGCTTGCTGCCGAAGCTTAATACTTTTGATGTTGGAACCAACTATCCGAG
TGGCGCTATAGCTACTAGCATCAGTGTGTGCACCAAGTTGAGGACACTGAATCTTACAAGGAACAAGC
TCGTGGGGGGGATACCGGAGAGCTTCAAGGAGTTGAGATCCTGGCCTAACTTTCCCTGAACCAGAATA
ATTTCCCAACCTGGCATAAACCTTGGAGGGCTTGGAACACCTGGCCAACCTGACAAGCTTGGTGCTGA
ACAAGAATTTCCCCCGCGGTGAAACCATGGAGGTGCTCCTCCTGACAAACTGGCTACTCATCCGCGTA
ATTTCCCCGTGGCTGCAGGGCGTGGGAAGGCTCAATGTGCTGGAAATTTCATGTACAAGTTAGATGGG
AATTTCCCGCCATGGATGGGGAAGCTAGACAATGCTTCAACATTGACCTCTCAACAATTCTTTCAGCGG
TAAGCTACTTGTCAGCTTCACACAGATGAGGAGCTTGATTCAAATAATGGCTCGAAGCCAGCGA
Gy220
GGGGGAATGGGGGAACTTCACTAGATCATGCACTTGACTTGTCCTATAACAAGTTCACAGGCTCCAT
CCCTGATGTCTTTGGAGAGATGAGGAGGCTAGAGTCCGTAAACCTGGCCACCAATAGGCTGGATGGT
GAGTTGCCCGCTTCCCTGTAAAGTTCTCCACTGCTGAGGGTAATCAGCTTGAGGAACAACTCGCTCTC
TGGTGAGATTGCTATCGACTCCAGCTTGCTGCCGAAGCTTAATACTTTTGATGTTGGAACCAACTATC
TGAGTGGCGCTATAGCTCCTGGCATCAGTGTGTGCACCAAGTTGAGGACACTGAATCTTACAAGGAA
CAAGCTCGTGGGGGAGATACCAGAGAGCTTCAAGGAGTTGAGATCCGTGGCAACCCTCCCCGAGAA
CGGAAAATTTTTCCACCCCGGGTAAAAACTTTGGGGGTTTTGAAACCCCCCCCCAACCTGAAAATTT
TGGGTGAAAACGAAAATTTCCCGCGGGGGGAAAAAAGGGGGGGTCCTCCTCGGAAAAAATGTTCTC
CTCTGCGGAAAATTCCCCCGGGGGCGGAAAATGTGGAAACCCTAAGGTGTGTAGAAATTTTGTTGA
AAATGTATAAGGAAATACTCCCCCGGGGGGGGGAAACCAAAACAGCTGCTCACTTTCTCTCTCAACA
ATTTTTTTCGGGAAGATCTCTGGTCTCTCACACAAGAGAGACTTGATTCAAAATAGGCCGGAAAGCA
AA
LXN
ACACATGTAGGAAATATCTAAGATCTGCAACTTGACTTGTCCTATAACAAGTTCACAGGCTCCATC

CCTGATGTCTTTGGAGAGATGAGGAGGCTAGAGTCCGTAAACCTGGCCACCAATAGGCTGGATG
GTGAGTTGCCCGCTTCCCTGTAAAGTTCTCCACTGCTGAGGGTAATCAGCTTGAGGAACTACTCG
CTCTCTGGTGAGATTGCTATCGACTCCAGCTTGCTGCCGAAGCTTAATACTTTTGATGTTGGAACC
AACTATCTGAGTGGCGCTATAGCTCCTGGCATCAGTGTGTGCACCAAGTTGAGGACACTGAATCT
TACAAGGAACAAGCTCGTGGGGGAGGATACAGAGCTTCTTCAAGGAGAGAAAATCGTTGTACCT
CTCCCTGATAACCAGGAATTTTTTCACATCCTGTATAAATTGTTGGAGTTGTTGCACCTCCTGCCC
CTCCTGACTTGGTGGTGATCACCAATTTTCTCCGCGGCGAAAAAACAATGGTGGTGCTCCTCATG
AAAAATTGTCTCCTCATCGGAATTCCCCCCCGGCGGCTGAAGATGTGGAGAATCCATGTTGTGGA
AAATTTTTGTTGTAACATGTAAGGGGATAATCCCCCGCCGTGGGTGGAGAATCTAGACTGCTGCT
ACAATGTTCACTTCTCAAACTATTTTTTCGGCGATATTCCTGTTGTCTTCTTCACACAGATGAGGAT
CTAGTCTTATAATGGTTCCTCGAGCAAGAGAA

GEMS15

GGGGGCCTTGGTACTCTCTAGATCATGCACTTGACTTGTCCTATAACAAGTTCACAGGCTCCATCC
CTGATGTCTTTGGAGAGATGAGGAGGCTAGAGTCCGTAAACCTGGCCACCAATAGGCTGGATGGT
GAGTTGCCCGCTTCCCTGTAAAGTTCTCCACTGCTGAGGGTAATCAGCTTGAGGAACAACTCGCTC
TCTGGTGAGATTGCTATCGACTCCAGCTTGCTGCCGAAGCTTAATACTTTTGATGTTGGAACCAACT
ATCTGAGTGGCGCTATAGCTCCTGGCATCAGTGTGTGCACCAAGTTGAGGACACTGAATCTTACAA
GGAACAAGCTCGTGGGGGAAAACCGAAAAACTTCAAGGGATTAAAAACCCTGGCCAACCCCCCCC
TGACCAGGAAAAATTTCCCAACCTGGCAAAAAAATTGGAGGGCTTGGAACCCCTGCCCAACCTGA
AAAGCTTGGGGCTGACCAGGAATTTCCCCGGGGGGGAAACAATGGAGGGGCTTCTCCTGACAAAC
TGTCTACCCATCGGGGTAATTCCCCCCGGGCTGCAAAACTTGGGAAGCCTCAATGTGCTGGAAATT
TCCGTGAACAAGTTAAATGGGAATATCCCGCCCTGGCTGGGGAAACTAAACAACTGCTTCCACCTT
GACCCTCCCAACAATTCTTCCCCCGTAAGCTACCGTGCCACTTCCACCAAATGGAGGACTTGAATT
TCAATATGGCTCGAGCAAGCAAAA
GEMS20
GGGGCCCTGGGTACCTCTCTCGATCATGCACTTGACTTGTCCTATAACAAGTTCACAGGCTCCATC
CCTGATGTCTTTGGAGAGATGAGGAGGCTAGAGTCCGTAAACCTGGCCACCAATAGGCTGGATGG
TGAGTTGCCCGCTTCCCTGTAAAGTTCTCCACTGCTGAGGGTAATCAGCTTGAGGAACAACTCGCT
CTCTGGTGAGATTGCTATCGACTCCAGCTTGCTGCCGAAGCTTAATACTTTTGATGTTGGAACCAA
CTATCTGAGTGGCGCTATAGCTCCTGGCATCAGTGTGTGCACCAAGTTGAGGACACTGAATCTTAC
AAGGAACAAGCTCGTGGGGGAAAACCGAAAAACTTCCAGGGATTAAAAACCCGGGCCAACCCTC
CCCTAAACCGGAAAAATTTCCCAACCCGGCAAAAAAATTGGAGGGTTTGGAAACCCCGCCCAACC
CGAAAAACTTGGGGGTAAACCGGAATTTCCCCGGGGGGGAAAAAATGGAGGGGGCCTTCCTGGA
AAAATGTCTACCCCTCGGGGTAATTCCCCCCGGGGTGGAAAATTTGGGAAACCTCCAGGGGCTGG
AAATTTTCGTGAACAAGTTAAAAGGGAATATCCCCCCCGTGCGTGGGAAACCTAAACACTGCTCTC
ACATTTGACCTCTCAACCATTTCTTTCCGCGGAAACTACCGTGTCCCTCTCCCACAAATGAGGGATC
TTGATTTCAATAAGGGCTCGGGCAAAGAAAA
GEMS41
AGGGCATGGGGAACTTCTCTCAGATCATGCAACTTGACTTGTCCTATAACAAGTTCACAGGCTCCA
TCCCTGATGTCTTTGGAGAGATGAGGAGGCTAGAGTCCGTAAACCTGGCCACCAATAGGCTGGAT
GGTGAGTTGCCCGCTTCCCTGTAAAGTTCTCCACTGCTGAGGGTAATCAGCTTGAGGAACAACTCG
CTCTCTGGTGAGATTGCTATCGACTCCAGCTTGCTGCCGAAGCTTAATACTTTTGATGTTGGAACCA
ACTATCTGAGTGGCGCTATAGCTCCTGGCATCAGTGTGTGCACCAAGTTGAGGACACTGAATCTTA
CAAGGAACAAGCTCGTGGGGGAAAACCGAAAAATTTTAAGGGATTAAAAACCCGTGCTAACCCTC
CCCTAACCCGAAAAATTTTCCCAACCGGGGTAAAAATTTTGAGGGTTTGGAAACCCGGCCCAACC
GAGAAAATTTGGGGTGAACCAGAAATTTCCCGGGGGGGAAACAAAGGGAGGGGTCCTCCCCAGA
AAATTTGTCATCCTCCGGGTAAATTCCCCCCGGGTTGGCAGATTGTGAAAACCTCAAGTGGTGGAG
AATTCTCGTGAAAAATTATAAGGGAATATCTCGCCCCGGGTGTGGGAAACTTAAAACTGCTTCTAC
ATTTAGCCCTCTAACAAATCTTTTTCCGCGAAACTATCTTGTACCTTCTCACAAAAGAAGAGATTGT
GATCTCAATAAGGGTCCGGGCACGAAAA
IRF314
GGGCCCTGGGGGTACTCTCTAGATCATGCAACTTGACTTGTCCTATAACAAGTTCACAGGCTCCAT
CCCTGATGTCTTTGGAGAGATGAGGAGGCTAGAGTCCGTAAACCTGGCCACCAATAGGCTGGATG
GTGAGTTGCCCGCTTCCCTGTAAAGTTCTCCACTGCTGAGGGTAATCAGCTTGAGGAACAACTCGC
TCTCTAGTGAGATTGCTATCGACTCCAGCTTGCTGCCGAAGCTTAATACTTTTGATGTTGGAACCAA
CTATCTGAGTGGCGCTATAGCTCCTGGCATCAGTGTGTGCACCAAGTTGAGGACACTGAATCTTAC
AAGGAACAAGCTCGTGGGGGAGATACCAGAGAGCTTCAAAGAGGTGAAATCCGGTGTAACCCTTC
CCGAACCCGAAAAATTTTTCCACCCCGGGTAAAAAATTTTGGGGTTTTGGCACCCCCCCCCCCCCG

AAAAATTTGGGGTGAACACGAAATTTTCCCGCGGGGGAAAAAAGGGGAGGGTCCTCCTCGGAAAA
AATTGTCTCCTCCGCGGAATTTCCCCCCGGGGGCTAAAAATGGGGAAACCCCACGGGGTGGGAAA
TTTTTGGTGAAAAATTTAAGGGGAAATTCTCCCCCGGGGGGGGGGAATTCAAAACAGCTTCTACA
ATTTTCTCCTCAAAAAATCTTTTTTCGGGGAAACACCTCTTGTCTTCTCACCACAGATGAGGATGTA
ATTTTAAAATAGGGCCTGGAAAAACAAAA

CIMBL 157

GGGCCAATGGGTACTCTCTAGATCATGCAACTTGACTTGTCCTATAACAAGTTCACAGGCTCCATC
CCTGATGTCTTTGGAGAGATGAGGAGGCTAGAGTCCGTAAACCTGGCCACCAATAGGCTGGATGG
TGAGTTGCCCGCTTCCCTGTAAAGTTCTCCACTGCTGAGGGTAATCAGCTTGAGGAACAACTCGCT
CTCTGGTGAGATTGCTATCGACTCCAGCTTGCTGCCGAAGCTTAATACTTTTGATGTTGGAACCAA
CTATCTGAGTGGCGCTATAGCTCCTGGCATCAGTGTGTGCACCAAGTTGAGGACACTGAATCTTAC
AAGGAACAAGCTCGTGGGGGAAATCCGAAAAATTTTCAGGGATTTGAAACCCGTGTCAAACCTCT
CTCAAACCGGAAATATTTTCAAACCCGGGCAAAAACTTTGCGGGTTTTGAAACCCCGGCCCACCCT
GAAAATTTGGGGGCTAACCGGAATTTCCCGGGGGGGAGACACAGGGCGGGGGCTCACCCTGACAA
AATGTCTACTCCTCGGTAAATCTCCCCCGGGTGTGCAGATTGTGGAAACCCACAGTGTTGTGAATA
TTCCGTGTAAATTATATATGGAAATACCGCGCCGTGTCGGGGAAATCTAAACACGCGCTTCCACAT
TGCTCTCTCAAACATTTCTTTCAGGGTAAGCTATGTGTCATTTCACACATATGAAGATTTGATTTCA
AAAGGGCAGGGCAACCAAA

GEMS39

TCGGCAGTGACCTTGGTAACCTCTCTCAGATCATGCAACTTGACTTGTCCTATAACAAGTTCACAG
GCTCCATCCCTGATGTCTTTGGAGAGATGAGGAGGCTAGAGTCCGTAAACCTGGCCACCAATAGG
CTGGATGGTGAGTTGCCCGCTTCCCTGTAAAGTTCTCCACTGCTGAGGGTAATCAGCTTGAGGAAC
AACTCGCTCTCTAGTGAGATTGCCATCGACTCCAGCTTGCTGCCGAAGCTTAATACTTTTGATGTTG
GAACCAACTATCTGAGTGGTGCTATAGCTCCTGGCATCAGTGTGTGCACCAAGTTGAGGACACTAA
ATCTTACAAGGAACAAGCTCGTGGGGGATACCGGAGAGCTTCAAGGAGTTGAGATCCCTGTCGTA
CCTCTCGCTGACCAGGAATAGTTTCACAACCTGGCATAGACATTGCAGGTCTTGCAACACCTGCCC
AACCTGACAAGCTTGGTGCTGACCAGGAATTTCCGCGGCGGTGAGACAATGCAGGTGCTCATCCT
GACAAACTATCTACTCATCGGCGTAATTCCCCCGTGGCTGCAGAGCTTGGGAAGCCTCAATGTGCT
GGAAATTTCATGTAACAAGTTAGATGGGAATATCCTGCCATGGCTGGGGAAGCTAGACAACTGCT
TCAACATTGATCTCTCAAACAATTCTTTCAGCCGTAAGCTACTTGTCAGCTTCACACAGATGAGGA
GCTTGAATTCAA

CML423

TCGGCAGTGACCTTGGTAACCTCTCTCAGATCATGCAACTTGACTTGTCCTATAACAAGTTCACAG
GCTCCATCCCTGATGTCTTTGGAGAGATGAGGAGGCTAGAGTCCGTAAACCTGGCCACCAATAGG
CTGGATGGTGAGTTGCCCGCTTCCCTGTAAAGTTCTCCACTGCTGAGGGTAATCAGCTTGAGGAAC
AACTCGCTCTCTAGTGAGATTGCCATCGACTCCAGCTTGCTGCCGAAGCTTAATACTTTTGATGTTG
GAACCAACTATCTGAGTGGTGCTATAGCTCCTGGCATCAGTGTGTGCACCAAGTTGAGGACACTAA
ATCTTACAAGGAACAAGCTCGTGGGGGATACCGGAGAGCTTCAAGGAGTTGAGATCCCTGTCGTA
CCTCTCGCTGACCAGGAATAGTTTCACAACCTGGCATAGACATTGCAGGTCTTGCAACACCTGCCC
AACCTGACAAGCTTGGTGCTGACCAGGAATTTCCGCGGCGGTGAGACAATGCAGGTGCTCATCCT
GACAAACTATCTACTCATCGGCGTAATTCCCCCGTGGCTGCAGAGCTTGGGAAGCCTCAATGTGCT
GGAAATTTCATGTAACAAGTTAGATGGGAATATCCTGCCATGGCTGGGGAAGCTAGACAACTGCT
TCAACATTGATCTCTCAAACAATTCTTTCAGCCGTAAGCTACTTGTCAGCTTCACACAGATGAGGA
GCTTGAATTCAA

GEMS39

TCGGCAGTGACCTTGGTAACCTCTCTCAGATCATGCAACTTGACTTGTCCTATAACAAGTTCACAG
GCTCCATCCCTGATGTCTTTGGAGAGATGAGGAGGCTAGAGTCCGTAAACCTGGCCACCAATAGG
CTGGACGGTGAGTTGCCCGCTTCCCTGTAAAGTTGTCCACTGCTGAGGGTAATCAGCTTGAGGAAC
TACTCGCTCTCTGGTGAGATTGCTATCGACTCCAGCTTGCTGCCGAAGCTTAATACTTTTGATGTTG
GAACCAACTATCTGAGTGGCGCTATAGCTCCTGGCATCAGTGTGTGCACCAAGTTGAGGACACTGA
ATCTTACAAGGAACAAGCTCGTGGGGGGGGATACCGGAGAGCTTCAAGGAGTTGAGATCCCTGTC
GTACCTCTCGCTGACCAGGAATAGTTTCACAACCTGGCATAGACATTGCAGGTCTTGCAACACCTG
CCCAACCTGACAAGCTTGGTGCTGACCAAGAATTTCCGCGGCGGTGAGACAATGCAGGTGCTCAT
CCTGACAAACTGTCTACTCATCGGCGTAATTCCCCCGTGGCTGCAGAGCTTGGGAAGCCTCAATGT
GCTGGAAATTTCATGTAACAAGTTAGATGGGAATATCCCGCCATGGCTGGGGAAGCTAGACAACT
GCTTCAACATTGACCTCTCAAACAATTCTTTCAGCGGTAAGCTACCTGTCAGCTTCACACAGATGA
GGAGCTTGAATTCAA

CML170

AATCAGTTCACTGGCAACCTCGGCAGTGACCTTGGTAACCTCTCTCAGATCATGCAACTTGACTTG
TCCTATAACAAGTTCACAGGCTCCATCCTTGATGTCTTTGGAGAGATGAGGAGGCTAGAGTCCGTA

AACCTGGCCACCAATAGGCTGGATGGTGAGTTGCCCGCTTCCCTGTAAAGTTCTCCACTGCTGAGG
GTAATCAGCTTGAGGAACAACTCGCTCTCTAGTGAGATTGCTATCGACTCCAGCTTGCTGCCGAAG
CTTAATACTTTTGATGTTGGAACCAACTATCTGAGTGGTGCTATAGCTCCTGGCATCAGTGTGTGCA
CCAAGTTGAGGACACTAAATCTTACAAGGAACAAGCTCGTGGGGGATACCGGAGAGCTTCAAGGA
GTTGAGATCCCTGTCGTACCTCTCGCTGACCAGGAATAGTTTCACAACCTGGCATAGACATTGCAG
GTCTTGCAACACCTGCCCAACCTGACAAGCTTGGTGCTGACCAGGAATTTCCGCGGTGGTGAGACA
ATGCAGGTGCTCATCCTGACAAACTATCTACTCATCGGCGTAATTCCCCCGTGGCTGCAGAGCTTG
GGAAGCCTCAATGTGCTGGAAATTTCATGTAACAAGTTAGATGGGAATATCCCGCCATGGCTGGG
GAAGCTAGACAACCGCTTCAACATTGACCTCTCAAACAATTCTTTCAGCGGTAAGCTACCTGTCAG
CTTCACACAGATGAGGAGCTTGAATTCAAATAATGGCTCGAGCAAGCGA

Gy237

TCGGCAGTGACCTTGGTAACCTCTCTCAGATCATGCAACTTGACTTGTCCTATAACAAGTTCACAG
GCTCCATCCCTGATGTCTTTGGAGAGATGAGGAGGCTAGAGTCCGTAAACCTGGCCACCAATAGG
CTGGACGGTGAGTTGCCCGCTTCCCTGTAAAGTTGTCCACTGCTGAGGGTAATCAGCTTGAGGAAC
TACTCGCTCTCTGGTGAGATTGCTATCGACTCCAGCTTGCTGCCGAAGCTTAATACTTTTGATGTTG
GAACCAACTATCTGAGTGGCGCTATAGCTCCTGGCATCAGTGTGTGCACCAAGTTGAGGACACTGA
ATCTTACAAGGAACAAGCTCGTGGGGGGGGATACCGGAGAGCTTCAAGGAGTTGAGATCCCTGTC
GTACCTCTCGCTGACCAGGAATAGTTTCACAACCTGGCATAGACATTGCAGGTCTTGCAACACCTG
CCCAACCTGACAAGCTTGGTGCTGACCAAGAATTTCCGCGGCGGTGAGACAATGCAGGTGCTCAT
CCTGACAAACTGTCTACTCATCGGCGTAATTCCCCCGTGGCTGCAGAGCTTGGGAAGCCTCAATGT
GCTGGAAATTTCATGTAACAAGTTAGATGGGAATATCCCGCCATGGCTGGGGAAGCTAGACAACT
GCTTCAACATTGACCTCTCAAACAATTCTTTCAGCGGTAAGCTACCTGTCAGCTTCACACAGATGA
GGAGCTTGAATTCAA

CML122

TCGGCAGTGACCTTGGTAACCTCTCTCAGATCATGCAACTTGACTTGTCCTATACCAAGTTCACAG
GCTCCATCCCTGATGTCTTTGGAGAGATGAGGAGGCTAGAGTCTGTAAACCTGGCCACCAATAGGC
TGGATGGTGAGTTGCCCGCTTCCCTGTAAAGTTCTCCACTGCTGAGGGTAATCAGCTTGAGGAACA
ACTCGCTCTCTGGTGAGATTGCTATCGACTCCAGCTTGTTGCCGAAGCTTAATACTTTTGATGTTGG
AACCAACTATCCGAGTGGCGCTATAGCTACTGGCATCAGTGTGTGCACCAAGTTGAGGACACTGA
ATCTTACAAGGAACATGCTCGTGGGGGGATACCGGAGAGCTTCAAGGAGTTGAGATCCCTGTCGT
ACCTCTCGCTGACCAGGAATAGTTTCACAACCTGGCATAGACATTGCAGGTCTTGCAACACCTGCG
CAACCTGACAAGCTTGGTGCTGACCAGGAATTTCCGCGGCGGTGAGACAATGCAGGTGCTCATCCT
GACAAACTGTCTACTCATCGGCGTAAATCCCACGTGGCTGCAGAGCTTGGGAAGCCTCAATGTGCT
GGAAATTTCATGTAACAAGTTAGATGGGAATATCCCGCCATGGCTGGGGAAGCTAGACAACTGCT
TCAACATTGACCTCTCAAACAATTCTTTCAGCGGTAAGCTATCTGTCAGCTTCACACAGATGAGGA
GCTTGAATTCAA

CIMBL71

AATCAGTTCACTGGCAACCTCGGCAGTGACCTTGGTAACCTCTCTCAGATCATGCAACTTGACTTG
TCCTATAACAAGTTCACAGGCTCCATCCCTGATTCCTTTGGAGAGATGAAGAGGCTAGAGTCCGTA
AACCTGGCCACCAATAGGCTGGATGGTGAGTTGCCCGCTTCCCTGTAAAGTTGTCCACTGCTGAGG
GTAATCAGCTTGAGGAACAACTCGCTCTCTGGTGAGATTGCTATCGACTCCAGCTTGCTGCCGAAG
CTTAATACTTTTGATGTTGGAACCAACAATCTGAGTGGCGCTATAGCTCCCGGCATCAGTGTGTGC
ACCAAGTTGAGGACACTGAATCTTACAAGGAACAAGCTCGTGGGGGAGATACTAGAGAGCTTCAA
GGAGTTGAGATCCCTGTCGTACCTCTCGCTGACCAGGAATAGCTTCACAACCTAGCATAGACATTG
CAGGTCTTGCAACACCTGCTCAACCTGACAAGCTTGGTGCTGACCAGGAATTTCCACGGCGGTGAG
ACAATGCAGGTGCTCATCCTGACAAATTGTCTACTCATCGGCGTAATTCCCCCGTGGCTGTAGAGC
TTGGGAAGCCTCAATGTGCTGGAAATTTCATGTAACAAGTTAGATGGGAATATCCCGCCATGGCTG
GGGAAGCTAGACAACCGCTTCAACATTGACCTCTCAAACAATTCTTTCAGCGGTAAGCTACCTGTC
AGCTTCACACAGATGAGGAGCTTGAATTCAAATAATGGCTCGAGCAAAGA

CML423

TCGGCAGTGACCTTGGTAACCTCTCTCAGATCATGCAACTTGACTTGTCCTATAACAAGTTCACAG
GCTCCATCCCTGATGTCTTTGGAGAGATGAGGAGGCTAGAGTCCGTAAACCTGGCCACCAATAGG
CTGGACGGTGAGTTGCCCGCTTCCCTGTAAAGTTGTCCACTGCTGAGGGTAATCAGCTTGAGGAAC
TACTCGCTCTCTGGTGAGATTGCTATCGACTCCAGCTTGCTGCCGAAGCTTAATACTTTTGATGTTG
GAACCAACTATCTGAGTGGCGCTATAGCTCCTGGCATCAGTGTGTGCACCAAGTTGAGGACACTGA
ATCTTACAAGGAACAAGCTCGTGGGGGGGGATACCGGAGAGCTTCAAGGAGTTGAGATCCCTGTC
GTACCTCTCGCTGACCAGGAATAGTTTCACAACCTGGCATAGACATTGCAGGTCTTGCAACACCTG
CCCAACCTGACAAGCTTGGTGCTGACCAAGAATTTCCGCGGCGGTGAGACAATGCAGGTGCTCAT
CCTGACAAACTGTCTACTCATCGGCGTAATTCCCCCGTGGCTGCAGAGCTTGGGAAGCCTCAATGT

GCTGGAAATTTCATGTAACAAGTTAGATGGGAATATCCCGCCATGGCTGGGGAAGCTAGACAACT
GCTTCAACATTGACCTCTCAAACAATTCTTTCAGCGGTAAGCTACCTGTCAGCTTCACACAGATGA
GGAGCTTGAATTCAA

GEMS55

AATCAGTTCACTGGCAACCTCGGCAGTGACCTTGGTAACCTCTCTCAGATCATGCAACTTGACTTG
TCCTATAATAAGTTCACAGGCTCCATACTTGATGTCTTTGGAGAGATGAGGAGGCTAGAGTCCGTA
AACCTGGCCACCAATAGGCTGGATGGTGAGTTGCCCGATTCCCTGTAAAGTTGTCCACTTCTGAGG
GTAATCAGCTTGAGGAACAACTCGCTCTCTGGTGAGATTGCTATCGACTCCAGCTTGCTGCCGAAG
CTTAATACTTTTGATGTTGGAACCAACTATCTGAGTGGCGCTATAGCTTCTAGCATCAGTGTGTGCA
CCAAGTTGAGGACACTGAATCTTACAAGGAACAAGCTCATGGGGGAGATACCAGAGAGCTTCAAG
GAGTTGAGATCCGTGTCGTACCTCTCGCTGACCAGGAATAGCTTCACAACCTGGCATAGACATTGC
AGGTCTTGCAACACCTGCCCAACCTGACAAGCTTGGTGCTGACCAGGAATTTCCGCGGTGGTGAGA
CAATGCAGGTGCTCATCCTGACAAACTGTCTACTCATCGGCGTAATTCCCCCGTGGCTGCAGAGGT
TGGGAAGCCTCAATGTGCTGGAAATTTCATGTAACAAGTTAGATGGGAATATCCCGCCATGGCTGG
GGAAGCTAGACAACCGCTTCAACATTGACCTCTCAAACAATTCTTTCAGCGGTAAGCTACCTGTCA
GCTTCACACAGATGAGGAGCTTGAATTCAAATAATGGCTCGAGCAAGCGA

CIMBL127

AATCAGTTCACTGGCAACCTCGGCAGTGACCTTGGTAACCTCTCTCAGATCATGCAACTTGACTTG
TCCTATAATAAGTTCACAGGCTCCATACTTGATGTCTTTGGAGAGATGAGGAGGCTAGAGTCCGTA
AACCTGGCCACCAATAGGCTGGATGGTGAGTTGCCCGATTCCCTGTAAAGTTGTCCACTTCTGAGG
GTAATCAGCTTGAGGAACAACTCGCTCTCTGGTGAGATTGCTATCGACTCCAGCTTGCTGCCGAAG
CTTAATACTTTTGATGTTGGAACCAACTATCTGAGTGGCGCTATAGCTTCTAGCATCAGTGTGTGCA
CCAAGTTGAGGACACTGAATCTTACAAGGAACAAGCTCATGGGGGAGATACCAGAGAGCTTCAAG
GAGTTGAGATCCGTGTCGTACCTCTCGCTGACCAGGAATAGCTTCACAACCTGGCATAGACATTGC
AGGTCTTGCAACACCTGCCCAACCTGACAAGCTTGGTGCTGACCAGGAATTTCCGCGGTGGTGAGA
CAATGCAGGTGCTCATCCTGACAAACTGTCTACTCATCGGCGTAATTCCCCCGTGGCTGCAGAGGT
TGGGAAGCCTCAATGTGCTGGAAATTTCATGTAACAAGTTAGATGGGAATATCCCGCCATGGCTGG
GGAAGCTAGACAACCGCTTCAACATTGACCTCTCAAACAATTCTTTCAGCGGTAAGCTACCTGTCA
GCTTCACACAGATGAGGAGCTTGAATTCAAATAATGGCTCGAGCAAGCGA

CIMBL77

AATCAGTTCACTGGCAACCTCGGCAGTGACCTTGGTAACCTCTCTCAGATCATGCAACTTGACTTG
TCCTATAACAAGTTCACAGGCTCCATCCCTGATGTCTTTGGAGAGATGAGGAGGCTAGAGTTCGTA
AACCTGGCCACCAATAGGCTGGATGGTGAGTTGCCCGCTTCCCTGTAAAGTTCTCCACTGCTGAGG
GTAATCAGCTTGAGGAACAACTCGCTCTCTGGTGAGATTGCTATCGACTCCAGCTTGCTGCCGAAG
CTTAATACTTTTGATGTTGGAACCAACTATCTGAGTGGCGCTATAGCTCCTGGCATCAGTGTGTGC
ACCAAGTTGAGGACACTGAATCTTACAAGGAACAAGCTCGTGGGGGGGGGATACCGGAGAGCTTC
AAGGAGTTGAGATCCCTGTCGTACCTCTCGCTGACCAGGAATAGTTTCACAACCTGGCATAGACAT
TGCAGGTCTTGCAACACCTGCCCAACCTGACAAGCTTGGTGCTGACCAGGAATTTCCGCGGCGGTG
AGACAATGCAGGTGCTCATCCTAACAAACTGTCTACTCATCGGCGTAATTCCCCCGTGGCTGCAGA
GGTTGGGAAGCCTCAATGTGCTGGAAATTTCATGTAACAAGTTAGATGGGAATATCCCGCCATGGC
TGGGGAAGCTAGACAACCGCTTCAACATTGACCTCTCAAACAATTCTTTCAGCGGTAAGCTACCTG
TCAGCTTCACACAGATGAGGAGCTTGAATTCAAATAATGGCTCGAGCAAGCGA

# The sequence of GRMZM2G511067

# Bigger starch granules size

Zhong69

AAGGTTAATATTTTTTAGTATTGACGAGTAATGTTTAGAGAGCAACCTGAGACATATATTTCCAAC
GCGAGAAGGCATGTTGCTTTTAGTGCTTGTGAAAATATTGCATCTCTATCTACGAAGATGGTTGAA
ATCGAAAAGTTATTGTGTTTCCCTTAGTATTCAAGCTAATTGTGTTTGCCTTATAATTACCCGTGTC
AATGACAGGTGTTGAGAGAATATTTTCGACAATACACATTACCAAGAAATGATTGCGTAATAAAA
GTGAAGACAATTGGATGGATGATTTGATGGTTTGCTATACAGAGAAATGGGCCTTCAAAATTCCTT
GATGATAAGATCATTAATAGAAGATTTTAGTAGCATAAGGGAGACTCAAATTTTTATGATCTTTTA
TATGTGA

CIMBL10

GAGTTAAATTTTTTTAGTATTGACGAGTAATGTTTAGAGAGCAACCTGAGACATATTTCCAACGCG
AGAAGGCATGTTGCTTTTAGTGCTTGTGAAAATATTGCATCTCTATCTACGAAGATGGTTGAAACC

GAAAAGTTCTTGTGTTTCCCTTAGTATTCAAGCTAATTGTGTTTGCCTTATAATTACCCGTGTCAAT
GACAAGTGTTGAGAGAATATTTTCGACAATACACATTACCAAGAAATGGTTGCGTAATAAAAGTG
AAGACAATTGGATGGATGATTTGATGGTTTGCTATACAGAGAAATGGACCTTCAAAATTCCTTGAT
GATAATACCATTACTAGAAGATTTTAGTAGCATAAGGGAGACTCAAATTTTTATGATCCTTTATAT
GTGA

CIMBL87

CATTCAACATTTTTTTAGTATTGAACGAGTAATGTTTAGAGAGCAACCTGAGACATATTTCCAACG
CGAGAAGGCATGTTGCTTTTAGTGCTTGTGAAAATATTGCATCTCTATCTACGAAGATGGTTGAAA
CCGAAAAGTTCTTGTGTTTCCCTTAGTATTCAAGCTAATTGTGTTTGCCTTATAATTACCCGTGTCA
ATGACAAGTGTTGAGAGAATATTTTCGACAATACACATTACCAAGAAATGGTTGCGTAATAAAAG
TGAAGACAATTGGATGGATGATTTGATGGTTTGCTATACAGAGAAATGGACCTTCAAAATTCCTTG
ATGATAATACCATTACTAGAAGATTTTAGTAGCATAAGGGAGACTCAAATTTTTATGATCCTTTAT
ATGTGA

CIMBL84

GGGTTACTATTTTTTTAAGTATTGACGAGTAATGTTTAGAGAGCAACCTGAGACATATTTCCAACG
CGAGAAGGCATGTTGCTTTTAGTGCTTGTGAAAATATTGCATCTCTATCTACGAAGATGGTTGAAA
CCGAAAAGTTCTTGTGTTTCCCTTAGTATTCAAGCTAATTGTGTTTGCCTTATAATTACCCGTGTCA
ATGACAAGTGTTGAGAGAATATTTTCGACAATACACATTACCAAGAAATGGTTGCGTAATAAAAG
TGAAGACAATTGGATGGATGATTTGATGGTTTGCTATACAGAGAAATGGACCTTCAAAATTCCTTG
ATGATAATACCATTACTAGAAGATTTTAGTAGCATAAGGGAGACTCAAATTTTTATGATCCTTTAT
ATGTGA

CIMBL91

AGTTAAAATTTTTTTAGTATTGACGAGTAATGTTTAGAGAGCAACCTGAGACATATTTCCAACGCG
AGAAGGCATGTTGCTTTTAGTGCTTGTGAAAATATTGCATCTCTATCTACGAAGATGGTTGAAACC
GAAAAGTTCTTGTGTTTCCCTTAGTATTCAAGCTAATTGTGTTTGCCTTATAATTACCCGTGTCAAT
GACAAGTGTTGAGAGAATATTTTCGACAATACACATTACCAAGAAATGGTTGCGTAATAAAAGTG
AAGACAATTGGATGGATGATTTGATGGTTTGCTATACAGAGAAATGGACCTTCAAAATTCCTTGAT
GATAATACCATTACTAGAAGATTTTAGTAGCATAAGGGAGACTCAAATTTTTATGATCCTTTATAT
GTGA

Nan21-3

GATTAAAAATTTTTTTAGTATTGAACGAGTATGTTTAGAGAGCAACCTGAGACATATTTCCAACGC
GAGAAGGCATGTTGCTTTTAGTGCTTGTGAAAATATTGCATCTCTATCTACGAAGATGGTTGAAAC
CGAAAAGTTCTTGTGTTTCCCTTAGTATTCAAGCTAATTGTGTTTGCCTTATAATTACCCGTGTCAA
TGACAAGTGTTGAGAGAATATTTTCGACAATACACATTACCAAGAAATGGTTGCGTAATAAAAGT
GAAGACAATTGGATGGATGATTTGATGGTTTGCTATACAGAGAAATGGACCTTCAAAATTCCTTGA
TGATAATACCATTACTAGAAGATTTTAGTAGCATAAGGGAGACTCAAATTTTTATGATCCTTTATA
TGTGA

CIMBL142

TATTAAAATTTTTTTAATAATTGAACGAGTAATGTTTAGAGAGCAACCTGAGACATATTTCCAAC
GCGAGAAGGCATGTTGCTTTTAGTGCTTGTGAAAATATTGCATCTCTATCTACGAAGATGGTTGAA
ACCGAAAAGTTCTTGTGTTTCCCTTAGTATTCAAGCTAATTGTGTTTGCCTTATAATTACCCGTGTC
AATGACAAGTGTTGAGAGAATATTTTCGACAATACACATTACCAAGAAATGGTTGCGTAATAAAA
GTGAAGACAATTGGATGGATGATTTGATGGTTTGCTATACAGAGAAATGGACCTTCAAAATTCCTT
GATGATAATACCATTACTAGAAGATTTTAGTAGCATAAGGGAGACTCAAATTTTTATGATCCTTTA
TATGTGA

CML325

CGTTATAATTTTTTTAGAAATTGACGAGTAAAGTTTAAAGAGCAACCTGAGACATATTTCCAACGC
GAGAAGGCATGTTGCTTTTAGTGCTTGTGAAAATATTGCATCTCTATCTACCAAGATGGTTGAAAC
CGAAAAGTTCTTGTGTTTCCCTTAGTATTCAAGCTAATTGTGTTTGCCTTATAATTACCCGTGTCAA
TGACAAGTGTTGAGAGAATATTTTCGACAATACACATTACCAAGAAATGGTTGCGTAATAAAAGT
GAAGACAATTGGATGGATGATTTGATGGTTTGCTATACAGAGAAATGGACCTTCAAAATTCCTTGA
TGATAATACCATTACTAGAAGATTTTAGTATCATAAGGGAGACTCAAATTTTTATGATCCTTTATAT
GTGA

04K5702

TGGTTTAAAATTTTTTTAGTATTTGACGAGTAATGTTTAGAGAGCAACCTGAGGCATCCATGCATG
TTGCTTTTAGTGCTTGTGAAAATATTGCATCTCTATCTACGAAGATGGTTGAAACAGAAAAGTTAT
TGTGTTTCCCTTAGTATTCAAGCTAATTGTGTTTGCCTTATAATTACCCGTGTCAATGACAAGTGTT
GAGAGAATATTTTCGACAAGACACATTACCAAGAAATGATTGCGTAATAAAAGTGAAGACAATTG
GATGGATGATTTGATGGTTTGCTATACAGAGAAATGGACCTTCAAAGTTCCTTGATGATAAAACCA
TTACTAGAAGATTTTAGTAGCATAAGGGAGACTCAAATTTTTATGATCCTTTATATGTGA

M153
AGTTAAATTTTTTTTAGTATTGACGAGTAATGTTTAGAGAGCAACCTGAGACATATATTTCCAACG
CGAGAAGGCATGTTGCTTTTAGTGCTTGTGAAAATATTGCATCTCTATCTACGAAGATGGTTGAAA
TCGAAAAGTTATTGTGTTTCCCTTAGTATTCAAGCTAATTGTGTTTGCCTTATAATTACCCGTGTCA
ATGACAGGTGTTGAGAGAATATTTTCGACAATACACATTACCAAGAAATGATTGCGTAATAAAAG
TGAAGACAATTGGATGGATGATTTGATGGTTTGCTATACAGAGAAATGGGCCTTCAAAATTCCTTG
ATGATAAGATCATTAATAGAAGATTTTAGTAGCATAAGGGAGACTCAAATTTTTATGATCCTTTAT
ATGTGA
CML121
ATAAAATTTTTTTAGTATTGACGAGTAATGTTTAGAGAGCAACCTGAGGCATCCATGCATGTTGCT
TTTAGTGCTTGTGAAAATATTGCATCTCTATCTACGAAGATGGTTGAAACAGAAAAGTTATTGTGT
TTCCCTTAGTATTCAAGCTAATTGTGTTTGCCTTATAATTACCCGTGTCAATGACAAGTGTTGAGAG
AATATTTTCGACAAGACACATTACCAAGAAATGATTGCGTAATAAAAGTGAAGACAATTGGATGG
ATGATTTGATGGTTTGCTATACAGAGAAATGGACCTTCAAAGTTCCTTGATGATAAAACCATTACT
AGAAGATTTTAGTAGCATAAGGGAGACTCAAATTTTTATGATCCTTTATATGTGA
Ye8001
GCGTATGCTGATTAGTATTGACGAGTAATGTTTATGAGAGCAACCTGAGACATATTTCCAACGCGA
GAAGGCATGTTGCTTTTAGTGCTTGTGAAAATATTGCATCTCTATCTACGAAGATGGTTGAAACCG
AAAAGTTCTTGTGTTTCCCTTAGTATTCAAGCTAATTGTGTTTGCCTTATAATTACCCGTGTCAATG
ACAAGTGTTGAGAGAATATTTTCGACAATACACATTACCAAGAAATGGTTGCGTAATAAAAGTGA
AGACAATTGGATGGATGATTTGATGGTTTGCTATACAGAGAAATGGACCTTCAAAATTCCTTGATG
ATAATACCATTACTAGAAGATTTTAGTAGCATAAGGGAGACTCAAATTTTTATGATCCTTTTATAT
GTGA
CIMBL91
ACGCATGCTGCATTGAGTATTGACGAGTAATGTTTATGAGAGCAACCTGAGACATATTTCCAACGC
GAGAAGGCATGTTGCTTTTAGTGCTTGTGAAAATATTGCATCTCTATCTACGAAGATGGTTGAAAC
CGAAAAGTTCTTGTGTTTCCCTTAGTATTCAAGCTAATTGTGTTTGCCTTATAATTACCCGTGTCAA
TGACAAGTGTTGAGAGAATATTTTCGACAATACACATTACCAAGAAATGGTTGCGTAATAAAAGT
GAAGACAATTGGATGGATGATTTGATGGTTTGCTATACAGAGAAATGGACCTTCAAAATTCCTTGA
TGATAATACCATTACTAGAAGATTTTAGTAGCATAAGGGAGACTCAAATTTTTATGATCTTTT
GEMS61
GAATTTATATTTTTTTAGTAATTGACGAGTAATGTTTAGAGAGCAACCTGAGACATATTTCCAACG
CGAGAAGGCATGTTGCTTTTAGTGCTTGTGAAAATATTGCATCTCTATCTACGAAGATGGTTGAAA
CCGAAAAGTTCTTGTGTTTCCCTTAGTATTCAAGCTAATTGTGTTTGCCTTATAATTACCCGTGTCA
ATGACAAGTGTTGAGAGAATATTTTCGACAATACACATTACCAAGAAATGGTTGCGTAATAAAAG
TGAAGACAATTGGATGGATGATTTGATGGTTTGCTATACAGAGAAATGGACCTTCAAAATTCCTTG
ATGATAATACCATTACTAGAAGATTTTAGTAGCATAAGGGAGACTCAAATTTTTATGATCCTTTAT
ATGTGA
3H-2
ACCTATGCTGATTTAGTATTGACGAGTAATGTTTATGAGAGCAACCTGAGACATATTTCCAACGCG
AGAAGGCATGTTGCTTTTAGTGCTTGTGAAAATATTGCATCTCTATCTACGAAGATGGTTGAAACC
GAAAAGTTCTTGTGTTTCCCTTAGTATTCAAGCTAATTGTGTTTGCCTTATAATTACCCGTGTCAAT
GACAAGTGTTGAGAGAATATTTTCGACAATACACATTACCAAGAAATGGTTGCGTAATAAAAGTG
AAGACAATTGGATGGATGATTTGATGGTTTGCTATACAGAGAAATGGACCTTCAAAATTCCTTGAT
GATAATACCATTACTAGAAGATTTTAGTAGCATAAGGGAGACTCAAATTTTTATGATCTTTTATATT
GTGA
835b
CACATATAAAGGATCATAAAAATTTGAGTCTCCCTTATGCTACTAAAATCTTCTAGTAATGGTATT
ATCATCAAGGAATTTTGAAGGTCCATTTCTCTGTATAGCAAACCATCAAATCATCCATCCAATTGT
CTTCACTTTTATTACGCAACCATTTCTTGGTAATGTGTATTGTCGAAAATATTCTCTCAACACTTGT
CATTGACACGGGTAATTATAAGGCAAACACAATTAGCTTGAATACTAAGGGAAACACAAGAACTT
TTCGGTTTCAACCATCTTCGTAGATAGAGATGCAATATTTTCACAAGCACCAAAAGCAACATGCCT
TCTCGCGTTGGAAATATGTCTCAGGTTGCTCTCTAAACATTACTCGTTCAATTACTAAAAAATATTG
ATCATATAGACTACCAAATCGAACAAGTTT
CIMBL88
CTTCGATGTTGTATCGATCTCGCGTATTTATTTCGCGAAATGTCAAGTATGGGGTCCGTTTAAAAAT
TTATAAATTACGAGAACATGAATTTGGAATCCCGACCCACTCTTGTGATTGTTGTCCTAAGTAAAT
ATTATTAGAACTTGATGACAACAATATTGACGCAAAATTAAATCGGATCGAGATAACTAGAACTG
AACTGAGTATTCATAATTAATCTGTTTCACTATCTACAACTGTGGAAATTTGTCTGGTCCGGAAAAT
ACCTACCATTGTAAGCGCAGTGCAAAAACATCAAAATTTATATTATTTTGATTTATCTGATTGCGTC

TCGTCCAGATCTAAGTATTCGGATATATATATAACCATACACAAATTATCGAACTGCGAGAATATT
C
K22
CACATATAAAGGATCATAACTTCGATGTTGTATCGATCTCGCGTATTTATTTCGCGAAATGTCAAG
TATGGGGTCCGTTTAAAAATTTATAAATTACGAGAACATGAATTTGGAATCCCGACCCACTCTTGT
GATTGTTGTCCTATGTAAATATTATTAGAACTTGATGACAACAATATTGACGCAAAATTAAATCGG
ATCGAGATAACTAGAACTGAACTGAGTATTCATAATTAATCTGTTTCACTATCTACAACTGTGGAA
ATTTGTCTGGTCCGGAAAATACCTACCATTGTAAGCGCAGTGCAAAAACATCAAAATTTATATTAT
TTTGATTTATCTGATTGCGTCTCGTCCAGATCTAAGTATTCGGATATATATATAACCATACACAAAT
TATCGAACTGCGAGAATATTCACCAAATCGAACAAGTTT
Zheng29
AAATTTGAGTCTCCCTTATGCTACTAAAATCTTCTAGTAATGGTATTATCATCAAGGAATTTTGAAG
GTCCATTTCTCTGTATAGCAAACCATCAAATCATCCATCCAATTGTCTTCACTTTTATTACGCAACC
ATTTCTTGGTAATGTGTACTGTCGAAAATATTCTCTCAACACTTGTCATTGACACGGGTAATTATAA
GGCAAACACAATTAGCTTGAATACTAAGGGAAACACAAGAACTTTTCGGTTTCAACCATCTTCGTA
GATAGAGATGCAATATTTTCACAAGCACTAAAAGCAACATGCCTTCTCGCGTTGGAAATATGTCTC
AGGTTGCTCTCTAAACATTACTCGTTCAATTACTAAAAAATATTGATCATATAGACT
CIMBL54
CACATATAAAGGATCATAAAAATTTGAGTCTCCCTTATGCTACTAAAATCTTCTATTAATGATCTTA
TCATCAAGGAATTTTGAAGGCCCATTTCTCTGTATAGCAAACCATCAAATCATCCATCCAATTGTCT
TCACTTTTATTACGCAATCATTTCTTGGTAATGTGTATTGTCGAAAATATTCTCTCAACACCTATCA
TTGACACGGGTAATTATAAGGCAAACACAATTAGCTTGAATACTAAGGGAAACACAATAACTTTT
CGATTTCAACCATCTTCGTAGATAGAGATGCAATATTTTCACAAGCACTAAAAGCAACATGCCTTC
TCGCGTTGGAAATATATGTCTCAGGTTGCTCTCTAAACATTACTCGTTCAATTACTAAAAAATATTG
ATCATATAGACTACCAAATCGAACAAGTTT
CIMBL142
CTTCGATGTTGTATCGATCTCGCGTATTTATTTCGCGAAATGTCAAGTATGGGGTCCGTTTAAAAAT
TTATAAATTACGAGAACATGTATTTGGAATCCCGACCCACTCTTGTGATTGTTGTCCTAAGTAAAT
ATTATTAGAACTTGATGACAACAATATTGACGCAAAATTAAATCGGATCGAGATAACTAGAACTG
AACTGAGTATCCATAATTAATCTGTTTCACTATCTACAACTGTGGAAATTTGTCTGGTCCGGAAAA
TACCTACCATTGTAAGCGCAGTGCAAAAACATCAAAATTTATATTATTTTGATTTATCTGATTGCGT
CTCGTCCAGATCTAAGTATTCGGATATATATATAACCATACACAAATTATCGAACTGCGAGAATAT
TC
ZaC546
CTTCGATGTTGTATCGATCTCGCGTATTTATTTCGCGAAATGTCAAGTATGGGGTCCGTTTAAAAAT
TTATAAATTACGAGAACATGAATTTGGAATCCCGACCCACTCTTGTGATTGTTGTCCTAAGTAAAT
ATTATTAGAACTTGATGACAACAATATTGACGCAAAATTAAATCGGATCGAGATAACTAGAACTG
AACTGAGTATTCATAATTAATCTGTTTCACTATCTACAACTGTGGAAATTTGTCTGGTCCGGAAAAT
ACCTACCATTGTAAGCGCAGTGCAAAAACATCAAAATTTATATTATTTTGATTTATCTGATTGCGTC
TCGTCCAGATCTAAGTATTCGGATATATATATAACCATACACAAATTATCGAACTGCGAGAATATT
C
Dan360
AAATTTGAGTCTCCCTTATGCTACTAAAATCTTCTAGTAATGGTATTATCATCAAGGAATTTTGAAG
GTCCATTTCTCTGTATAGCAAACCATCAAATCATCCATCCAATTGTCTTCACTTTTATTACGCAACC
ATTTCTTGGTAATGTGTATTGTCGAAAATATTCTCTCAACACTTGTCATTGACACGGGTAATTATAA
GGCAAACACAATTAGCTTGAATACTAAGGGAAACACAAGAACTTTTCGGTTTCAACCATCTTCGTA
GATAGAGATGCAATATTTTCACAAGCACTAAAAGCAACATGCCTTCTCGCGTTGGAAATATGTCTC
AGGTTGCTCTCTAAACATTACTCGTTCAATTACTAAAAAATATTGATCATATAGACT
CML470
CACATATAAAGGATCATAAAAATTTGAGTCTCCCTTATGCTACTAAAATCTTCTATTAATGATCTTA
TCATCAAGGAATTTTGAAGGCCCATTTCTCTGTATAGCAAACCATCAAATCATCCATCCAATTGTCT
TCACTTTTATTACGCAGTCATTTCTTGGTAATGTGTATTGTCGAAAATATTCTCTCAACACCTGTCA
TTGACACGGGTAATTATAAGGCAAACACAATTAGCTTGAATACTAAGGGAAACACAATAACTTTT
CGATTTCAACCATCTTCGTAGATAGAGATGCAATATTTTCACAAGCACTAAAAGCAACATGCCTTC
TCGCGTTGGAAATATATGTCTCAGGTTGCTCTCTAAACATTACTCGTTCAATTACTAAAAAATATTG
ATCATATAGACTACCAAATCGAACAAGTTT
Xun971
AAATTTGAGTCTCCCTTATGCTACTAAAATCTTCTAGTAATGGTATTATCATCAAGGAATTTTGAAG
GTCCATTTCTCTGTATAGCAAACCATCAAATCATCCATCCAATTGTCTTCACTTTTATTACGCAACC
ATTTCTTGGTAATGTGTATTGTCGAAAATATTCTCTCAACACTTGTCATTGACACGGGTAATTATAA

GGCAAACACAATTAGCTTGAATACTAAGGGAAACACAAGAACTTTTCGGTTTCAACCATCTTCGTA
GATAGAGATGCAATATTTTCACAAGCACTAAAAGCAACATGCCTTCTCGCGTTGGAAATATGTCTC
AGGTTGCTCTCTAAACATTACTCGTTCAATTACTAAAAAATATTGATCATATAGACT

FCD0602

CACATATAAAGGATCATAAAAATTTGAGTCTCCCTTATGCTACTAAAATCTTCTATTAATGATCTTA
TCATCAAGGAATTTTGAAGGCCCATTTCTCTGTATAGCAAACCATCAAATCATCCATCCAATTGTCT
TCACTTTTATTACGCAGTCATTTCTTGGTAATGTGTATTGGTCGAAAATATTCTCTCAACACCTGTC
ATTGACACGTTGGTAATTATAAGGCAAAACACAATTAGCTTGAATACTAAGGGAAACACAATAAC
TTTTCGATTTCAACCATCTTCGTAGATAGAGATGCAATATTTTCACAAGCACTAAAAGCAACATGC
CTTCTCGCGTTGGAAATATATGTCTCAGGTTGCTCTCTAAACATTACTCGTTCAATTACTAAAAAAT
ATTGATCATATAGACTACCAAATCGAACAAGTTT

# Smaller starch granules size

GEMS41
GGTAATTGTTTAGTATTGACGAGTAATGTTTAGAGAGCAACCTGAGGCATCCATGCATGTTGCTTT
TAGTGCTTGTGAAAATATTGCATCTCTATCTACGAAGATGGTTGAAACAGAAAAGTTATTGTGTTT
CCCTTAGTATTCAAGCTAATTGTGTTTGCCTTATAATTACCCGTGTCAATGACAAGTGTTGAGAGA
ATATTTTCGACAAGACACATTACCAAGAAATGATTGCGTAATACAAGTGAAGACAATTGGATGGA
TGATTTGATGGTTTGCTATACAGAGAAATGGACCTTCAAAGTTCCTTGATGATAAAACCATTACTA
GAAGATTTTATTAGCATAAGGGAGACTCAAATTTTTATGATCCTTTATATGTGA

GEMS15
AATAATATTTTTTAGTATTGACGAGTAATGTTTAGAGAGCAACCTGAGGCATCCATGCATGTTGCT
TTTAGTGCTTGTGAAAATATTGCATCTCTATCTACGAAGATGGTTGAAACAGAAAAGTTATTGTGT
TTCCCTTAGTATTCAAGCTAATTGTGTTTGCCTTATAATTACCCGTGTCAATGACAAGTGTTGAGAG
AATATTTTCGACAAGACACATTACCAAGAAATGATTGCGTAATAAAAGTGAAGACAATTGGATGG
ATGATTTGATGGTTTGCTATACAGAGAAATGGACCTTCAAAGTTCCTTGATGATAAAACCATTACT
AGAAGATTTTAGTAGCATAAGGGAGACTCAAATTTTTATGATCCTTTATATGTGA

GEMS20
AGGAAATTTTTTAGTATTGACGAGTATGTTTAGAGAGCAACCTGAGACATATTTCCAACGCGAGAA
GGCATGTTGCTTTTAGTGCTTGTGAAAATATTGCATCTCTATCTACGAAGATGGTTGAAACCGAAA
AGTTCTTGTGTTTCCCTTAGTATTCAAGCTAATTGTGTTTGCCTTATAATTACCCGTGTCAATGACA
AGTGTTGAGAGAATATTTTCGACAATACACATTACCAAGAAATGGTTGCGTAATAAAAGTGAAGA
CAATTGGATGGATGATTTGATGGTTTGCTATACAGAGAAATGGACCTTCAAAATTCCTTGATGATA
ATACCATTACTAGAAGATTTTAGTAGCATAAGGGAGACTCAAATTTTTATGATCCTTCTATATGTG
ACCAA

CIMBL77
CATATAATTTTTTTAGTATTGACGAGTAATGTTTAGAGAGCAACCTGAGACATATTTCCAACGCGA
GAAGGCATGTTGCTTTTAGTGCTTGTGAAAATATTGCATCTCTATCTACGAAGATGGTTGAAACCG
AAAAGTTCTTGTGTTTCCCTTAGTATTCAAGCTAATTGTGTTTGCCTTATAATTACCCGTGTCAATG
ACAAGTGTTGAGAGAATATTTTCGACAATACACATTACCAAGAAATGGTTGCGTAATAAAAGTGA
AGACAATTGGATGGATGATTTGATGGTTTGCTATACAGAGAAATGGACCTTCAAAATTCCTTGATG
ATAATACCATTACTAGAAGATTTTAGTAGCATAAGGGAGACTCAAATTTTTATGATCTTTTTATATG
TGA

B111
CCACCGTCATTATAGCTTAGTATTGACGAGTATGTTTAGAGAGCAACCTGAGACATATTTCCAACG
CGAGAAGGCATGTTGCTTTTAGTGCTTGTGAAAATATTGCATCTCTATCTACGAAGATGGTTGAAA
CCGAAAAGTTCTTGTGTTTCCCTTAGTATTCAAGCTAATTGTGTTTGCCTTATAATTACCCGTGTCA
ATGACAAGTGTTGAGAGAATATTTTCGACAATACACATTACCAAGAAATGGTTGCGTAATAAAAG
TGAAGACAATTGGATGGATGATTTGATGGTTTGCTATACAGAGAAATGGACCTTCAAAATTCCTTG
ATGATAATACCATTACTAGAAGATTTTAGTAGCATAAGGGAGACTCAAATTTTTATGATCCTTTTA
TATGTGACCAA

Sy999
ATACGTCCGATTTCATTTTAGTATTGACGAGTATGTTTAGAGAGCACCTGAGACATATATTTCCAA
CGCGAGAAGGCATGTTGCTTTTAGTGCTTGTGAAAATATTGCATCTCTATCTACGAAGATGGTTGA
AATCGAAAAGTTATTGTGTTTCCCTTAGTATTCAAGCTAATTGTGTTTGCCTTATAATTACCCGTGT
CAATGACAGGTGTTGAGAGAATATTTTCGACAATACACATTACCAAGAAATGATTGCGTAATAAA
AGTGAAGACAATTGGATGGATGATTTGATGGTTTGCTATACAGAGAAATGGGCCTTCAAAATTCCT
TGATGATAAGATCATTAATAGAAGATTTTAGTAGCATAAGGGAGACTCAAATTTTTATGATCCTTT
ATATGTGA

Gy220
TAAAAATTTTTTTAGTATTGACGAGTAATGTTTAGAGAGCAACCTGAGACATATTTCCAACGCGA
GAAGGCATGTTGCTTTTAGTGCTTGTGAAAATATTGCATCTCTATCTACGAAGATGGTTGAAACCG
AAAAGTTCTTGTGTTTCCCTTAGTATTCAAGCTAATTGTGTTTGCCTTATAATTACCCGTGTCAATG
ACAAGTGTTGAGAGAATATTTTCGACAATACACATTACCAAGAAATGGTTGCGTAATAAAAGTGA
AGACAATTGGATGGATGATTTGATGGTTTGCTATACAGAGAAATGGACCTTCAAAATTCCTTGATG
ATAATACCATTACTAGAAGATTTTAGTAGCATAAGGGAGACTCAAATTTTTATGATCCTTGATATG
TGACC
LXN
AGTTAATTTTTTTAGTATTGACGAGTATGTTTAGAGAGCAACCTGAGACATATTTCCAACGCGAGA
AGGCATGTTGCTTTTAGTGCTTGTGAAAATATTGCATCTCTATCTACGAAGATGGTTGAAACCGAA
AAGTTCTTGTGTTTCCCTTAGTATTCAAGCTAATTGTGTTTGCCTTATAATTACCCGTGTCAATGAC
AAGTGTTGAGAGAATATTTTCGACAATACACATTACCAAGAAATGGTTGCGTAATAAAAGTGAAG
ACAATTGGATGGATGATTTGATGGTTTGCTATACAGAGAAATGGACCTTCAAAATTCCTTGATGAT
AATACCATTACTAGAAGATTTTAGTAGCATAAGGGAGACTCAAATTTTTATGATCCTTTTATATGT
GACCA
GEMS39
AAATTCGAGTCTCCCTTATGCTACTAAAATCTTCTAGTAATGGTATTATCATCAAGGAATTTTGAAG
GTCCATTTCTCTGTATAGCAAACCATCAAATCATCCATCCAATTGTCTTCACTTTTATTACGCAACC
ATTTCTTGGTAATGTGTATTGTCGAAAATATTCTCTCAACACTTGTCATTGACACGGGTAATTATAA
GGCAAACACAATTAGCTTGAATACTAAGGGAAACACAAGAACTTTTCGGTTTCAACCATCTTCGTA
GATAGAGATGCAATATTTTCACAAGCACTAAAAGCAACATGCCTTCTCGCGTTGGAAATATGTCTC
AGGTTGCTCTCTAAACATTACTCGTTCAATTACTAAAAAAATATTGATCATATAGACT
CIMBL157
AAATTTGAGTCTCCCTTATGCTACTAAAATCTTCTATTAATGATCTTATCATCAAGGAATTTTGAAG
GCCCATTTCTCTGTATAGCAAACCATCAAATCATCCATCCAATTGTCTTCACTTTTATTACGCAATC
ATTTCTTGGTAATGTGTATTGTCGAAAATATTCTCTCAACACCTGTCATTGACACGGGTAATTATAA
GGCAAACACAATTAGCTTGAATACTAAGGGAAACACAATAACTTTTCGATTTCAACCATCTTCGTA
GATAGAGATGCAATATTTTCACAAGCACTAAAAGCAACATGCCTTCTCGCGTTGGAAATATATGTC
TCAGGTTGCTCTCTAAACATTACTCGTTCAATTACTAAAAAAATATTGATCATATAGACT
GEMS55
AAATTTGAGTCTCCCTTATGCTACTAAAATCTTCTAGTAATGGTATTATCATCAAGGAATTTTGAAG
GTCCATTTCTCTGTATAGCAAACCATCAAATCATCCATCCAATTGTCTTCACTTTTATTACGCAACC
ATTTCTTGGTAATGTGTATTGTCGAAAATATTCTCTCAACACTTGTCATTGACACGGGTAATTATAA
GGCAAACACAATTAGCTTGAATACTAAGGGAAACACAAGAACTTTTCGGTTTCAACCATCTTCGTA
GATAGAGATGCAATATTTTCACAAGCACTAAAAGCAACATGCCTTCTCGCGTTGGAAATATGTCTC
AGGTTGCTCTCTAAACATTACTCGTTCAATTACTAAAAAAATATTGATCATATAGACT
CIMBL157
GGCATGCTGATTTAGTATTGACGAGTAATGTTTATGAGAGCAACCTGAGACATATATTTCCAACGC
GAGAAGGCATGTTGCTTTTAGTGCTTGTGAAAATATTGCATCTCTATCTACGAAGATGGTTGAAAT
CGAAAAGTTATTGTGTTTCCCTTAGTATTCAAGCTAATTGTGTTTGCCTTATAATTACCCGTGTCAA
TGACAGGTGTTGAGAGAATATTTTCGACAATACACATTACCAAGAAATGATTGCGTAATAAAAGT
GAAGACAATTGGATGGATGATTTGATGGTTTGCTATACAGAGAAATGGGCCTTCAAAATTCCTTGA
TGATAAGATCATTAATAGAAGATTTTAGTAGCATAAGGGAGACTCAAATTTTTATGATCTTTTTAT
ATGTGATA
Gy237
GCCAATGCTGCATTAGTATTGACGAGTAATGTTTATGAGAGCAACCTGAGACATATTTCCAACGCG
AGAAGGCATGTTGCTTTTAGTGCTTGTGAAAATATTGCATCTCTATCTACGAAGATGGTTGAAACC
GAAAAGTTCTTGTGTTTCCCTTAGTATTCAAGCTAATTGTGTTTGCCTTATAATTACCCGTGTCAAT
GACAAGTGTTGAGAGAATATTTTCGACAATACACATTACCAAGAAATGGTTGCGTAATAAAAGTG
AAGACAATTGGATGGATGATTTGATGGTTTGCTATACAGAGAAATGGACCTTCAAAATTCCTTGAT
GATAATACCATTACTAGAAGATTTTAGTAGCATAAGGGAGACTCAAATTTTTATGATCTTTTTATAT
TGTGA
GEMS39
ACGCAGGCTGATTGAGTCTGAGAAGTAATGTTTATGAGAGCAACCTGAGACATATTTCCAACGCG
AGAAGGCATGTTGCTTTTAGTGCTTGTGAAAATATTGCATCTCTATCTACGAAGATGGTTGAAACC
GAAAAGTTCTTGTGTTTCCCTTAGTATTCAAGCTAATTGTGTTTGCCTTATAATTACCCGTGTCAAT
GACAAGTGTTGAGAGAATATTTTCGACAATACACATTACCAAGAAATGGTTGCGTAATAAAAGTG
AAGACAATTGGATGGATGATTTGATGGTTTGCTATACAGAGAAATGGACCTTCAAAATTCCTTGAT
GATAATACCATTACTAGAAGATTTTAGTAGCATAAGGGAGACTCAAATTTTTATGATCTTTTAATA

TGTGA
GEMS55
ACGATCCTGACTGAGTATTGACGAGTAATGTTTATAGAGCAACCTGAGACATATTTCCAACGCGAG
AAGGCATGTTGCTTTTAGTGCTTGTGAAAATATTGCATCTCTATCTACGAAGATGGTTGAAACCGA
AAAGTTCTTGTGTTTCCCTTAGTATTCAAGCTAATTGTGTTTGCCTTATAATTACCCGTGTCAATGA
CAAGTGTTGAGAGAATATTTTCGACAATACACATTACCAAGAAATGGTTGCGTAATAAAAGTGAA
GACAATTGGATGGATGATTTGATGGTTTGCTATACAGAGAAATGGACCTTCAAAATTCCTTGATGA
TAATACCATTACTAGAAGATTTTAGTAGCATAAGGGAGACTCAAATTTTTATGATCTT
IRF314
CACATATAAAGGATCATAAAAATTTGAGTCTCCCTTATGCTACTAAAATCTTCTAGTAATGGTATT
ATCATCAAGGAATTTTGAAGGTCCATTTCTCTGTATAGCAAACCATCAAATCATCCATCCAATTGT
CTTCACTTTTATTACGCAACCATTTCTTGGTAATGTGTATTGTCGAAAATATTCTCTCAACACTTGT
CATTGACACGGGTAATTATAAGGCAAACACAATTAGCTTGAATACTAAGGGAAACACAAGAACTT
TTCGGTTTCAACCATCTTCGTAGATAGAGATGCAATATTTTCACAAGCACTAAAAGCAACATGCCT
TCTCGCGTTGGAAATATGTCTCAGGTTGCTCTCTAAACATTACTCGTTCAATTACTAAAAAATATTG
ATCATATAGACTACCAAATCGAACAAGTTT
CML170
CACATATAAAGGATCATAAAAATTTGAGTCTCCCTTATGCTACTAAAATCTTCTATTAATGATCTTA
TCATCAAGGAATTTTGAAGGCCCATTTCTCTGTATAGCAAACCATCAAATCATCCATCCAATTGTCT
TCACTTTTATTACGCAATCATTTCTTGGTAATGTGTATTGTCGAAAATATTCTCTCAACACCTGTCA
TTGACACGGGTAATTATAAGGCAAACACAATTAGCTTGAATACTAAGGGAAACACAATAACTTTT
CGATTTCAACCATCTTCGTAGATAGAGATGCAATATTTTCACAAGCACTAAAAGCAACATGCCTTC
TCGCGTTGGAAATATATGTCTCAGGTTGCTCTCTAAACATTACTCGTTCAATTACTAAAAAATATTG
ATCATATAGACTACCAAATCGAACAAGTTT
CML423
CACATATAAAGGATCATAAAAATTTGAGTCTCCCTTATGCTACTAAAATCTTCTATTAATGATCTTA
TCATCAAGGAATTTTGAAGGCCCATTTCTCTGTATAGCAAACCATCAAATCATCCATCCAATTGTCT
TCACTTTTATTACGCAATCATTTCTTGGTAATGTGTATTGTCGAAAATATTCTCTCAACACCTATCA
TTGACACGGGTAATTATAAGGCAAACACAATTAGCTTGAATACTAAGGGAAACACAATAACTTTT
CGATTTCAACCATCTTCGTAGATAGAGATGCAATATTTTCACAAGCACTAAAAGCAACATGCCTTC
TCGCGTTGGAAATATATGTCTCAGGTTGCTCTCTAAACATTACTCGTTCAATTACTAAAAAATATTG
ATCATATAGACTACCAAATCGAACAAGTTT
CIMBL127
AAATTTGAGTCTCCCTTATGCTACTAAAATCTTCTATTAATGATCTTATCATCAAGGAATTTTGAAG
GCCCATTTCTCTGTATAGCAAACCATCAAATCATCCATCCAATTGTCTTCACTTTTATTACGCAATC
ATTTCTTGGTAATGTGTATTGTCGAAAATATTCTCTCAACACCTGTCATTGACACGGGTAATTATAA
GGCAAACACAATTAGCTTGAATACTAAGGGAAACACAATAACTTTTCGATTTCAACCATCTTCGTA
GATAGAGATGCAATATTTTCACAAGCACTAAAAGCAACATGCCTTCTCGCGTTGGAAATATATGTC
TCAGGTTGCTCTCTACATTACTCGTTCAATTACTAAAAAATATTGATCATATAGACT
CML122
CACATATAAAGGATCATAAAAATTTGAATCTCCCTTATGCTACTAAAATCTTCTAGTAATGGTATT
ATCATCAAGGAATTTTGAAGGTCCATTTCTCTGTATAGCAAACCATCAAATCATCCATCCAATTGT
CTTCACTTTTATTACGCAACCATTTCTTGGTAATGTGTATTGTCGAAAATATTCTCTCAACACTTGT
CATTGACACGGGTAATTATAAGGCAAACACAATTAGCTTGAATACTAAGGGAAACACAAGAACTT
TTCGGTTTCAACCATCTTCGTAGATAGAGATGCAATATTTTCACAAGCACTAAAAGCAACATGCCT
TCTCGCGTTGGAAATATGTCTCAGGTTGCTCTCTAAACATTACTCGTTCAATTACTAAAAAATATTG
ATCATATAGACTACCAAATCGAACAAGTTT
CIMBL71
AAATTTGAGTCTCCCTTATGCTACTAAAATCTTCTAGTAATGGTTTTATCATCAAGGAACTTTGAAG
GTCCATTTCTCTGTATAGCAAACCATCAAATCATCCATCCAATTGTCTTCACTTTTATTACGCAATC
ATTTCTTGGTAATGTGTCTTGTCGAAAATATTCTCTCAACACTTGTCATTGACACGGGTAATTATAA
GGCAAACACAATTAGCTTGAATACTAAGGGAAACACAATAACTTTTCTGTTTCAACCATCTTCGTA
GATAGAGATGCAATATTTTCACAAGCACTAAAAGCAACATGCATGGATGCCTCAGGTTGCTCTCTA
AACATTACTCGTTCAATTACTAAAAAATATTGATCATATAGACT

# The sequence of GRMZM2G419660

## Bigger starch granules size

ZaC546
GGTAGGAATCTGCATAATATAAAGAAGCATGAATTAAGACCTTAAAACAGAAGTCACTCAAAGTG
GGAGCCAAAAAACAGAAATATAAGGGAACTATCCAATCTAGCACAGCTCTGTCCTATACTTCAGT
TCAAGGCAATGGAGGGGATACCTATTCAGAAGCAATCCGGCAGCAATTGGAGCAACAACTATTTG
AACGATGCTGGACATCATCCCTTTGACATCTACAGGTAATTTCTGGCCAATAAGAAAGTAAGATAA
TGTTGGGGTGACAAAAACTGCAGTAGCTGTTGACAATGATGTCATAACAATGCTGAGAGGAGCCA
TATGTGGATCCGTCAAGAAAGTTGCATAGTTTGAAAGTTGTGCCCCGCTCACGCATGAAACCGACA
TTATGCCAGCGCCTACAGACAAGTTCACGTCAGAATTCACTACATATGCTGCAATGCTTATTCTAA
AGCATATAAAAATGCATGAACTCAATTTCTTGCCTAGAGCAGCTGGGAGGTTAAGAACTGCAACC
GCAAGAGTGCCAAAAAGGAATCCAAACAAAGGCTTGATGACAAACTGTCCGATATAGCCCGCAGC
AATAGCATCTGGCCTCTTTATTGCTTCAATAAAATCCTTGGCACTTGAGTTTACACCAACAGCAAA
CATCAAGAACCCCAAAGCTGGTGCATAATACCTGCAATAATAACACACACCCTGGAAATGTAAAT
AACAGGTCCAAATATAGAGCACTGTGCTACTGACAAAAACATGAAACTATCTAATTGTATAAAAG
CCAGCTGATAAGTGAGA
835b
TAGTCAAATATATATGAAAAAAAAACTTTAGTACAAATATATATATGAAAAAATGGAATGGAGAA
ACTGGTAATTCGAGTTACTGAACTCTTTGTTGTTTTGGAATAGCAGCATCAGGCAAGAGTTGGAGA
ACTAGGAGTCTTCGGCTGTCGCTACGGCCTTTGGATTGCTCCTGTATTCTTCAATGGACAATCCTTG
ACACGGTCACATAAACACAACAAGAGGAGAACCCCGACCACAGTTCCCAGTCCAAGGGCGACGAT
GGCGGATTTGCTTTTATTGCTGAACAATGGGAACCCTCTGTGGGTGACCGCTTGCTCGAGCCATTA
TTTGAATTCAAGCTCCTCATCTGTGTGAAGCTGACAAGTAGCTTACGGCTGAAAGAATTGTTTGAG
AGGTCAATGTTGAAGCAGTTGTCTAGCTTCCCCAGCCATGGCGGGATATTCCCATCTAACTTGTTA
CATGAAACTTCCAGCACATTGAGGCTTCCCAAGCTCTGCAGCCACGGGGGAATTACGCCGATGAG
TAGACAGTTTGTCAGGATGAGCACCTACATTGTCTCACCGCCGCGGAAATTCCTGGTCAGCACCAA
GCTTGTCAGGTTGGGCAGGTGTTGCAAGACCTGCAATGTCTATGCCAGGTTGTGAAACTATTCCTG
GTCAGCGAGAGGTACGACAGGGATCTCAACTCCTTGAAGCTCTCCGGTATCCCCCCACGAGCTTGT
TCCTTGTAAAATTCAGTGTCCTCAACTTGGTGCACACACTGATGCCAGTAGCTATAGCGCCACTCG
GATAGTTGGTTCCAACATCAAAAGTATTAAGCTTCGGCAACAAGCTGGAGTCGATAGCAATCTCAC
CAGAGAGCGAGTTGTTCCTCAAGCTGATTACCCTCAGCAGTGGAGAACTTTACAGGGAAGCGGGC
AACTCACCATCCAGCCTATTGGTGGCCAGGTTTACAGACTCTAGCCTCCTCATCTCTCCAAAGACA
TCAGGGATGGAGCCTGTGAACTTGGTATAGGACAAGTCAAGTTGCATGAT
CML325
TAGTCAAATATATATGAAAAAAAACTTTAGTACAAATATATATATGAAAAAATGGGATGGAGAAAC
TGGTAATTCGAGTTACTAAACTCTTTGTTGTTTTGGAATAGCAGCACCAGGCAAGAGTTGGAGAAC
TAGGAGTCTTCGGTTGTCGCTACGGCCTTCGGATTGCTCCTGTATTCTTCAATGGACAATCCTTGAC
ACGATCACATAAGCACAACAAGAGGAGAAACCCGACCACAGTTCCTAGTCCAAGGGCAACGATG
GCGGCTTTGCTTTTATTGCTGAACAATGGGAGCCCTCTGTGGGTGATCGCTTGCTCGAGCCATTATT
TGAATTCAAGCTCCTCATCTGTGTGAAGCTGACAGGTAGCTTACCGCTGAAAGAATTGTTTGAGAG
GTCAATGTTGAAGCGGTTGTCTAGCTTCCCCAGCCATGGCGGGATATTCCCATCTAACTTGTTACAT
GAAATTTCCAGCACATTGAGGCTTCCCAACCTCTGCAGCCACGGGGGAATTACGCCGATGAGTAG
ACAGTTTGTCAGGATGAGCACCTGCATTGTCTCACCACCGCGGAAATTCCTGGTCAGCACCAAGCT
TGTCAGGTTGGGCAGGTGTTGCAAGACCTGCAATGTCTATGCCAGGTTGTGAAGCTATTCCTGGTC
AGCGAGAGGTACGACACGGATCTCAACTCCTTGAAGCTCTCTGGTATCTCCCCCATGAGCTTGTTC
CTTGTAAGATTCAGTGTCCTCAACTTGGTGCACACACTGATGCTAGAAGCTATAGCGCCACTCAGA
TAGTTGGTTCCAACATCAAAAGTATTAAGCTTCGGCAGCAAGCTGGAGTCGATAGCAATCTCACCA
GAGAGCGAGTTGTTCCTCAAGCTGATTACCCTCAGAAGTGGACAACTTTACAGGGAATCGGGCAA
CTCACCATCCAGCCTATTGGTGGCCAGGTTTACGGACTCTAGCCTCCTCATCTCTCCAAAGACATC
AAGTATGGAGCCTGTGAACTTATTATAGGACAAGTCAAGTTGCATGAT
CIMBL91
TAGTCAAATATATATGAAAAAAAAACTTTAGTACAAATATATATATGAAGAAATGGGATGGAGAA
ACTGGTAATTCGAGTTACTGAACTCTTTGTTGTTTTGGAATAGCAGCATCAGGCAAGAGTTGGAGA
ACTAGGAGTCTTCGGCTGTCGCTACGGCCTTTGGATTGCTCCTGTATTCTTCAATGGACAATCCTTG
ACACGGTCACATAAACACAACAAGAGGAGAACCCCGACCACAGTTCCCAGTCCAAGGGCGACGAT
GGCGGATTTGCTTTTATTGCTGAACAATGGGAACCCTCTGTGGGTGACCGCTTGCTCGAGCCATTA
TTTGAATTCAAGCTCCTCATCTGTGTGAAGCTGACAAGTAGCTTACGGCTGAAAGAATTGTTTGAG
AGGTCAATGTTGAAGCAGTTGTCTAGCTTCCCCAGCCATGGCGGGATATTCCCATCTAACTTGTTA
CATGAAATTTCCAGCACATTGAGGCTTCCCAAGCTCTGCAGCCACGGGGGAATTACGCCGATGAGT
AGACAGTTTGTCAGGATGAGCACCTACATTGTCTCACCGCCGCGGAAATTCCTGGTCAGCACCAAG
CTTGTCAGGTTGGGCAGGTGTTGCAAGACCTGCAATGTCTATGCCAGGTTGTGAAACTATTCCTGG
TCAGCGAGAGGTACGACAGGGATCTCAACTCCTTGAAGCTCTCCGGTATCCCCCCACGAGCTTGTT

CCTTGTAAAATTCAGTGTCCTCAACTTGGTGCACACACTGATGCCAGTAGCTATAGCGCCACTCGG
ATAGTTGGTTCCAACATCAAAAGTATTAAGCTTCGGCAACAAGCTGGAGTCGATAGCAATCTCACC
AGAGAGCGAGTTGTTCCTCAAGCTGATTACCCTCAGCAGTGGAGAACTTTACAGGGAAGCGGGCA
ACTCACCATCCAGCCTATTGGTGGCCAGGTTTACAGACTCTAGCCTCCTCATCTCTCCAAAGACAT
CAAGTATGGAGCCTGTGAACTTATTATAGGACAAGTCAAGTTGCATGAT

04K5702

TAGTCAAATATATATGAAAAAAACTTTAGTACAAATATATATATGAAAAAATGGGATGGAGAAAC
TGGTAATTCGAGTTACTGAACTCTTTGTTGTTTTGGAATAGCAGCACCAGGCAAGAGTTGGAGAAC
TAGGAGTCTTCGGTTGTCGCTACGGCCTTCGGATTGCTCCTGTATTCTTCAATGGACAATCCTTGAC
ACGATCACATAAGCACAACAAGAGGAGAAACCCGACCACAGTTCCTAGTCCAAGGGCAACGATG
GCGGCTTTGCTTTTATTGCTGAACAATGGGAGCCCTCTGTGGGTGATCGCTTGCTCGAGCCATTATT
TGAATTCAAGCTCCTCATCTGTGTGAAGCTGACAGGTAGCTTACCGCTGAAAGAATTGTTTGGGAG
GTCAATGTTGAAGCGGTTGTCTAGCTTCCCCAGCCATGGCGGGATATTCCCATCTAACTTGTTACAT
GAAATTTCCAGCACATTGAGGCTTCCCAACCTCTGCAGCCACGGGGGAATTACGCCGATGAGTAG
ACAGTTTGTCAGGATGAGCACCTGCATTGTCTCACCACCGCGGAAATTCCTGGTCAGCACCAAGCT
TGTCAGGTTGGCAGGTGTTGCAAGACCTGCAACGTCTATGCCAGGTTGTGAAGCTATTCCTGGTCA
GCGAGAGGTACGACACGGATCTCAACTCCTTGAAGCTCTCTGGTATCTCCCCCATGAGCTTGTTCC
TTGTAAGATTCAGTGTCCTCAACTTGGTGCACACACTGATGCTAGAAGCTATAGCGCCACTCAGAT
AGTTGGTTCCAACATCAAAAGTATTAAGCTTCGGCAGCAAGCTGGAGTCGATAGCAATCTCACCA
GAGAGCGAGTTGTTCCTCAAGCTGATTACCCTCAGCAGTGGAGAACTTTACAGGGAAGCGGGCAA
CTCACCATCCAGCCTATTGGTGGCCAGGTTTACGAACTCTAGCCTCCTCATCTCTCCAAAGACATC
AGGGATGGAGCCTGTGAACTTGTTATAGGACAAGTCAAGTTGCATGAT

GEMS61

TAGTCAAATATATATGAAAAAAACTTTAGTACAAATATATATATGAAAAAATGGGATGGAGAAAC
TGGTAATTCGAGTTACTGAACTCTTTGTTGTTTTGGAATAGCAGCACCAGGCAAGAGTTGGAGAAC
TAGGAGTCTTCGGTTGTCGCTACGGCCTTCGGATTGCTCCTGTATTCTTCAATGGACAATCCTTGAC
ACGATCACATAAGCACACCAAGAGGAGAAACCCGACCACAGTTCCTAGTCCAAGGGCAACGATGG
CGGCTTTGCTTTTATTGCTGAACAATGGGAGCCCTCTGTGGGTGATCGCTTGCTCGAGCCATTATTT
GAATTCAAGCTCCTCATCTGTGTGAAGCTGACAGGTAGCTTACCGCTGAAAGAATTGTTTGAGAGG
TCAATGTTGAAGCGGTTGTCTAGCTTCCCCAGCCATGGCGGGATATTCCCATCTAACTTGTTACATG
AAATTTCCAGCACATTGAGGCTTCCCAACCTCTGCAGCCACGGGGGAATTACGCCGATGAGTAGA
CAGTTTGTCAGGATGAGCACCTGCATTGTCTCACCACCGCGGAAATTCCTGGTCAGCACCAAGCTT
GTCAGGTTGGGCAGGTGTTGCAAGACCTGCAATGTCTATGCCAGGTTGTGAAGCTATTCCTGGTCA
GCGAGAGGTACGACACGGATCTCAACTCCTTGAAGCTCTCTGGTATCTCCCCCATGAGCTTGTTCC
TTGTAAGATTCAGTGTCCTCAACTTGGTGCACACACTGATGCTAGAAGCTATAGCGCCACTCAGAT
AGTTGGTTCCAACATCAAAAGTATTAAGCTTCGGCAGCAAGCTGGAGTCGATAGCAATCTCACCA
GGGAGCGAGTTGTTCCTCAAGCTGAT

Zhong69

TAGTCAAATATATATGAAAAAAACTTTAGTACAAATATATATATGAAAAAATGGGATGGAGAAAC
TGGTAATTCGAGTTACTGAACTCTTTGTTGTTTTGGAATAGCAGCACCAGGCAAGAGTTGGAGAAC
TAGGAGTCTTCGGTTGTCGCTACGGCCTTCGGATTGCTCCTGTATTCTTCAATGGACAATCCTTGAC
ACGATCACATAAGCACAACAAGAGGAGAAACCCGACCACAGTTCCTAGTCCAAGGGCAACGATG
GCGGCTTTGCTTTTATTGCTGAACAATGGGAGCCCTCTGTGGGTGATCGCTTGCTCGAGCCATTATT
TGAATTCAAGCTCCTCATCTGTGTGAAGCTGACAGGTAGCTTACCGCTGAGAGAATTGTTTGAGAG
GTCAATGTTGAAGCGGTTGTCTAGCTTCCCCAGCCATGGCGGGATATTCCCATCTAACTTGTTACAT
GAAATTTCCAGCACATTGAGGCTTCCCAACCTCTGCAGCCACGGGGGAATTACGCCGATGAGTAG
ACAGTTTGTCAGGATGAGCACCTGCATTGTCTCACCACCGCGGAAATTCCTGGTCAGCACCAAGCT
TGTCAGGTTGGGCAGGTGTTGCAAGACCTGCAATGTCTATGCCAGGTTGTGAAGCTATTCCTGGTC
AGCGAGAGGTACGACACGGATCTCAACTCCTTGAAGCTCTCTGGTATCTCCCCCATGAGCTTGTTC
CTTGTAAGATTCAGTGTCCTCAACTTGGTGCACACACTGATGCTAGAAGCTATAGCGCCACTCAGA
TAGTTGGTTCCAACATCAAAAGTATTAAGCTTCGGCAGCAAGCTGGAGTCGATAGCAATCTCACCA
GAGAGCGAGTTGTTCCTCAAGCTGATTACCCTCAGAAGTGGACAACTTTACAGGGAATCGGGCAG
CTCACCATCCAGCCTATTGGTGGCCAGGTTTACGGACTCTAGCCTCCTCATCTCTCCAAAGACATC
AAGTATGGAGCCTGTGAACTTATTATAGGACAAGTCAAGTTGCATGAT

CIMBL10

AAGCTCCGCACTTCGCTCTGAAAGCGAACCCTCCAACTAGAGTTGTGCAGTAATGGCAGCATCACG
AGCGATCGTGAGCCTGTGCTTCTCGTCTTCTGCCACCTTTCTAGTGTCTTGAGCCACATAAAACATC
TTCTAGAGCTCTACAAAAGAGGGAATATGAGCAACCCAATACGTAGAACAACCAAGCACAATTCG
GTTCCTAGAAGGGTGTACCGCTAGACACCTCACGCTCCCAAAGGAGCTCCACGTCTAGTTGCTCTA
GGTGAGCATTAGTACTTGATTTTAATTCCCCTAAAGGTATCAAATGTTGGGGGCCTTCCTCTTCCGA

AGGTCCTCAAATAACATTACTAACCATTTGCTTTCAGCATACTACTAAACATTATAGGAGCTTTGTC
ACCAGAGAAGCTTCATAACAGAAGAGAAGTATGACAAAGAAAGCTGCATCAAGCAAGCTTCATCA
TGGTGAAACAGGGAGACAACGTAAGGAGAAGCTGTTGTTCAAGTCATTAAATAGAAATGGCGATG
CTGTACTTGTGACATTTGTAACCCCTATGTGTAACTGTTGAGGGCATGAATGTAATAACGCACGAA
GCTTTGCGACTGTCTATAAATCGATGAACAGTACCCCCGTATTGTTCACGCTGGATTGTAATTGCCC
TCACGTCATTACCTTCAAACAAGCCGAAGGTATCATTGTAATACAAACTTTGAGAATATATTTATA
ATCATTATGAAATGAATACATGGCATGAAATTACATCTTGCGAGTATGTTTTTATACTTGTGGATA
ATGTAAACTTGTTACTTATGACCTTCGTCTAAGATTCATTATACCTAAGGGGGTATAATGCTTCGAA
GGACGAAGGTCTTTAACATATAACAAATGTGTTGCCTTGTTCTTGATTCACATCAGTTGAGAACAA
GTGAACAACATTGGTGCCCACCTCTGGTGAACTCGAACGACCACCTTCGACAACAAGAACTCGAA
CAACTACCTTCGCCATGCCACCGAAGAAGGTTGTAGTGCCAGGGGCTGCCCTTGCACCGCTGGACA
TCAATTAAGAAGGGCTCCCT
zheng58
AAGCTCCGCACTTCGCCCTGAAAGCGAACCCTCCAACTAGAGTTGTGCAGTAATGGCAGCATCAC
GAGCGATCGTGAGCCTGTGCTTCTCGTCTTCTGCCACCTTTCTAGTGTCTTGAGCCACATAAAACAT
CTTCTAGAGCTCTACAAAAGAGGGAATATGAGCAACCCAATACGTAGAACAACCAAGCACAATTC
GGTTCCTAGAAGGGTGTACCGCTAGACACCTCACGCTCCCAAAGGAGCTCCACGTCTAGTTGCTCT
AGGTGAGCATTAGTACTTGATTTTAATTCCCCTAAAGGTATCAAATGTTGGGGGCCTTCCTCTTCCG
AAGGTCCTCAAATAACATTACTAACCATTTGCTTTCAGCATACTACTAAACATTATAGGAGCTTTG
TCACCAGAGAAGCTTCATAACAGAAGAGAAGTATGACAAAGAAAGCTGCATCAAGCAAGCTTCAT
CATGGTGAAACAGGGAGACAACGTAAGGAGAAGCTGTTGTTCAAGTCATTAAATAGAAATGGCGA
TGCTGTACTTGTGACATTTGTAACCCCTATGTGTAACTGTTGAGGGCATGAATGTAATAACGCACG
AAGCTTTGCGACTGTCTATAAATCGATGAACAGTACCCCCGTATTGTTCACGCTGGATTGTAATTG
CCCTCACGTCATTACCTTCAAACAAGCCGAAGGTATCATTGTAATACAAACTTTGAGAATATATTT
ATAATCATTATGAAATGAATACATGGCATGAAATTACATCTTGCGAGTATGTTTTTATACTTGTGG
ATAATGTAAACTTGTTACCTATGACCTTCGTCTAAGATTCATTATACCTAAGAGGGTATAATGCTTC
GAAGGACGAAGGTCTTTAACATATAACAAATGTGTTGCCTTGTTCTTGATTCACATCAGTTGAGAA
CAAGTGAACAACATTGGTGCCCACCTCTGGTGAACTCGAACGACCACCTTCGACAACAAGAACTC
GAACAACTACCTTCGCCATGCCACCGAAGAAGGTTGTAGTGCCAGGGGCTGCCCTTGCACCGCTG
GACATCAATTAAGAAGGGCTCCCT
Ye8001
TAGTCAAATATATATGAAAAAAACTTTAGTACAAATATATATATGAAAAAATGGGATGGAGAAAC
TGGTAATTCGAGTTACTGAACTCTTTGTTGTTTTGGAATAGCAGCACCAGGCAAGAGTTGGAGAAC
TAGGAGTCTTCGGCTGTCGCTACGGCCTTCGGATTGCTCCTGTATTCTTCAATGGACAATCCTTGAC
ACGGTCACATAAGCACAACAAGAGGAGAACCCCGACCACAGTTCCCAGTCCAAGGGCGACGATG
GCGGATTTGCTTTTATTGCTGAACAATGGGAACCCTCTGTGGGTGACCGCTTGCTCGAGCCATTATT
TGAATTCAAGCTCCTCATCTGTGTGAAGCTGACAGGTAGCTTACCGCTGAAAGAATTGTTTGAGAG
GTCAATGTTGAAGCAGTTGTCTAGCTTCCCTAGCCATGGCGGGATATTCCCATCTAACTTGTTACAT
GAAATTTCCAGCACATTGAGGCTTCCCAAGCTCTGTAGCCACGGGGGAATTACGCCGATGAGCAG
ACAGTTTGTTAGGATGAGCACCTGCATTGTCTCACCGCCGCGGAAATTCCTGGTCAGCACCCAGCT
TGTCAGGTTGGGCAGGTGTTGCAAGACCTGCAATGTCTATGCCAGGTTGTGAAACTATTCCTGGTC
AGCGAGAGGTACGACAGGGATCTCAACTCCTTGAAGCTCTCCGGTATCCCCCCCCACGAGCTTGTT
CCTTGTAAGATTCAGTGTCCTCAACTTGGTGCACACACTGATGCCAGGAGCTATAGCGCCACTCAG
ATAGTTGGTTCCAACATCAAAAGTATTAAGCTTCGGCAGCAAGCTGGAGTCGATAGCAATCTCACC
AGAGAGCGAGTTGTTCCTCAAGCTGATTACCCTCAGCAGTGGAGAACTTTACAGGGAAGCGGGCA
ACTCACCATCCAGCCTATTGGTGGCCAGGTTTACGAACTCTAGCCTCCTCATCTCTCCAAAGACAT
CAGGGATGGAGCCTGTGAACTTGTTATAGGACAAGTCAAGTTGCATGAAT
CIMBL84
TAGTCAAATATATATGAAAAAAACTTTAGTACAAATATATATATGAAAAAATGGGATGGAGAAAC
TGGTAATTCGAGTTACTGAACTCTTTGTTGCTTTGGAATAGCAGCACCAGGCAAGAGTTGGAGAAC
TAGGAGTCTTCGGTTGTCGCTACGGCCTTCGGATTGCTCCTGTATTCTTCAATGGACAATCCTTGAC
ACGATCACATAAGCACAACAAGAGGAGAAACCCGACCACAGTTCCTAGTCCAAGGGCAACGATG
GCGGCTTTGCTTTTATTGCTGAACAATGGGAGCCCTCTGTGGGTGATCGCTTGCTCGAGCCATTATT
TGAATTCAAGCTCCTCATCTGTGTGAAGCTGACAGGTAGCTTACCGCTGAAAGAATTGTTTGAGAA
GTCAATGTTGAAGCGGTTGTCTAGCTTCCCCAGCCATGGCGGGATATTCCCATCTAACTTGTTACAT
GAAATTTCCAGCACATTGAGGCTTCCCAACCTCTGCAGCCACGGGGGAATTACGCCGATGAGTAG
ACAGTTTGTCAGGATGAGCACCTGCATTGTCTCACCACCGCGGAAATTCCTGGTCAGCACCAAGCT
TGTCAGGTTGGGCAGGTGTTGCAAGACCTGCAATGTCTATGCCAGGTTGTGAAGCTATTCCTGGTC
AGCGAGAGGTACGACACGGATCTCAACTCCTTGAAGCTCTCTGGTATCTCCCCCATGAGCTTGTTC
CTTGTAAGATTCAGTGTCCTCAACTTGGTGCACACACTGATGCTAGAAGCTATAGCGCCACTCAGA

TAGTTGGTTCCAACATCAAAAGTATTAAGCTTCGGCAGCAAGCTGGAGTCGATAGCAATCTCACCA
GAGAGCGAGTTGTTCCTCAAGCTGATTACCCTCAGAAGTGGACAACTTTACAGGGAATCGGGCAA
CTCACCATCCAGCCTATTGGTGGCCAGGTTTACGGACTCTAGCCTCCTCATCTCTCCAAAGACATC
AAGTATGGAGCCTGTGAACTTATTATAGGACAAGTCAAGTTGCATGAT

Zheng29

CAATGGGGAGGAAGAGAGTAGCTGTTGGGAAACTTTCTGCTAGTAGGAAAGATGGTTGCAATAAT
CCTCCTCAACCATGGAAGATACTGCCATGGATGGTACTGATAGGGTTCAAGATGATTCCTTTATGG
TCAGTGCCTCCTCCCTACCAGATGTTGGTGGAGGTGAACCTGTAACTAATGGTGATGAGCTTGCTG
GTGATCATGTGCCAGTACCACCCTAGAATGCTAAATCATATCTTTCCATGGCAAAGTGCTGGAAGC
ATATGGATACGCGGGAAGGTGTCGACAATGGTATAAAAAGTTATATTGCTATATGTCACTATTCTA
AAACTGAGTTGAGTGCAGATTCTACTAGTGACACTAGGCATTTAATTCGACACTCCGAAACTTATT
TGAAAAAAAGTAGGGCAAATTGTTGATGGTGTGCAAACTATTAATGATTTTGAGATAAAGAATAG
ATTCTCTCTATTACTCTTGATAATGCTTTCTCATATACAAATGCGATTGAAGCTCTTACCCCTCATCT
GCAGTCATACATAGATGATTATGTTATCCACAAAAGGTATGTGTGCCACATCATAAATCTGGTTGT
TCAAGATGACATAATAGTTGTTAGTAAATAATTGGATAATATTTGTGTTGTTATTCGCTTCATAACA
AGTACCTCTCAAATGATTACAAATTTTGTTGAGTACTACAAGACAAATAATATGAAGCCAACCACC
ATGAGCTAGAGTATTGCAAGACCACATCAGCTGCACTTGTGGTCTAGCTGCCACCGTGTACTAGGG
AACGAGACCCGCAACATTTCAACCAGAAGCTCGATAGTGAAGACGGCGGGGAGCAT

CIMBL54

TAGTCAAATATATATGAAAAAAAAACTTTAGTACAAATATATATATGAAAAAATGGGATGGAGAA
ACTGGTAATTCGAGTTACTGAACTCTTTGTTGTTTTGGAATAGCAGCATCAGGCAAGAGTTGGAGA
ACTAGGAGTCTTCGGCTGTCGCTACGGCCTTTGGATTACTCCTGTATTCTTCAATGGACAATCCTTG
ACACGGTCACATAAACACAACAAGAGGAGAACCCCGACCACAGTTCCCAGTCCAAGGGCGACGAT
GGCGGATTTGCTTTTATTGCTGAACAATGGGAACCCTCTGTGGGTGACCGCTTGCTCGAGCCATTA
TTTGAATTCAAGCTCCTCATCTGTGTGAAGCTGACAAGTAGCTTACGGCTGAAAGAATTGTTTGAG
AGGTCAATGTTGAAGCAGTTGTCTAGCTTCCCCAGCCATGGCGGGATATTCCCATCTAACTTGTTA
CATGAAATCTCCAGCACATTGAGGCTTCCCAAGCTCTGCAGCCACGGGGGAATTACGCCGATGAG
TAGACAGTTCGTCAGGATGAGCACCTACATTGTCTCACCGCCGCGGAAATTCCTGGTCAGCACGAA
GCTTGTCAGGTTGGGCAGGTGTTGCAAGACCTGCAATGTCTATGCCAGGTTGTGAAACTATTCCTG
GTCAGCGAGAGGTACGACAGGGATCTCAACTCCTTGAAGCTCTCTGGTATCCCCCCACGAGCTTGT
TCCTTGTAAAATTCAGTGTCCTCAACTTGGTGCACACACTGATGCCAGTAGCTATAGCGCCACTCG
GATAGTTGGTTCCAACATCAAAAGTATTAAGCTTCGGCAACAAGCTGGAGTCGATAGCAATCTCAC
CAGAGAGCGAGTTGTTCCTCAAGCTGATTACCCTCAGGAGTGGAGAACTTTACAGGGAAGCGGGC
AACTCACCATCCAGCCTATTGGTGGCCAGGTTTACAGACTCTAGCCTCCTCATCTCTCCAAAGACA
TCAGGGATGGAGCCTGTGAACTTGGTATAGGACAAGTCAAGTTGCATGAT

CIMBL142

CAATGGGGAGGAAGAGAGTAGCTGTTAGGAAACTTTCTGCTAGTAGGAAAGATGGTTGCAATAAT
CCTCCTCAACCATGGAAGATACTGCCATGGATGGTACTGATAGGGTTCAAGATGATTCCTTTATGG
TCAGTGCCTCCTCCCTACCAGATGTTGGTGGAGGTGAACCTGTAACTAATGGTGATGAGCTTGCTG
TGATCATGTGCCAGTACCACCCTAGAATGCTAAATCATATCTTTCCATGGCAAAGTGCTGGAAGCA
TATGGATACGCGGGAAGGTGTCGACAATGGTATAAAAAGTTATATTGCTATATGTCACTATTCTAA
AACCGAGTTGAGTGCAGATTCTACTAGTGACACTAGGCATTTAATTCGACACTCCGAAACTTATTT
GAAAAAAAGTAGGGCAAATTGTTGATGGTGTGCAAACTATTAATGATTTTGAGATAAAGAATAGA
TTCTCTCTATTACTCTTGATAATGCTTTCTCATATACAAATGCGATTGAAGCTCTTACCCCTCATCTG
CAGTCATACATAGATGATTATGTTATCCACAAAAGGTATGTGTGCCACATCATAAATCTGGTTGTT
CAAGATGACATAATAGTTGTTAGTAAATAATTGGATAATATTTGTGTTGTTATTCGCTTCATAACA
AGTACCTCTCAAATGATTACAAATTTTGTTGAGTACTACAAGACAAATAATATGAAGCCAACCACC
ATGAGCTAGAGTATTGCAAGACCACATCAGCTGCACTTGTGGTCTAGCTGCCACCGTGTACTAGGG
AACGAGACCCGCAACATTTCAACCAGAAGCTCGATAGTGAAGACAGCGGGGAGCAT

Dan360

GGTAGGAATCTGCATAATATAAAGAAGCATGAATTAAGACCTTAAAACAGAAGTCACTCAAAGTG
GGAGCCAAAAAACAGAAATATAAGGGAACTATCCAATCTAGCACAGCTCTGTCCTATACTTCAGT
TCAAGGCAATGGAGGGGATACCTATTCAGAAGCAATCCGGCAGCAATTGGAGCAACAACTATTTG
AACGATGCTGGACATCATCCCTTTGACATCTACAGGTAATTTCTGGCCAATAAGAAAGTAAGATAA
TGTTGGGGTGACAAAAACTGCAGTAGCTGTTGACAATGATGTCATAACAATGCTGAGAGGAGCCA
TATGTGGATCCGTCAAGAAAGTTGCATAGTTTGAAAGTTGTGCTCCGCTCACGCATGAAACCAACA
TTATGCCAGCGCCTACAGACAAGTTCACGTCAGAATTCACTACATATGCTGCAATGCTTATTCTAA
AGCATATAAAAATGCATGAACTCAATTTCTTGCCTAGAGCAGCTGGGAGGTTAAGAACTGCAACC
GCAAGAGTGCCAAAAAGGAATCCAAACAAAGGCTTGATGACAAACTGTCCGATATAGCCCGCAGC
AATAGCATCTGGCCTCTTTATTGCTTCAATAAAATCCTTGGCACTTGAGTTTACACCAACAGCAAA

CATCAAGAACCCCAAAGCTGGTGCATAATACCTGCAATAATAACACACACCCTGGAAATGTAAAT
AACAGGTCCAAATATAGAGCACTGTGCTACTGACAAAAACATGAAACTATCTAATTGTATAAAAG
CCAGCTGATAAGTGAGA

CML470

TAGTCAAATATATATGAAAAAAACTTTAGTACAAATATATATATGAAAAAATGGGATGGAGAAAC
TGGTAATTCGAGTTACTGAACTCTTTGTTGTTTTGGAATAGCAGCACCAGGCAAGAGTTGGAGAAC
TAGGGGTCTTCGGCTGTCGCTACGGCCTTCGGATTGCTCCTGTATTCTTCAATGGACAATCCTTGAC
ACGGTCACATAAGCACAACAAGAGGAGAACCCCGACCACAGTTCCCAGTCCAAGGGCGACGATG
GCGGATTTGCTTTTATTGCTGAACAATGGGAACCCTCTGTGGGTGACCGCTTGCTCGAGCCATTATT
TGAATTCAAGCTCCTCATCTGTGTGAAGCTGACAGGTAGCTTACCGCTGAAAGAATTGTTTGAGAG
GTCAATGTTGAAGCAGTTGTCTAGCTTCCCTAGCCATGGCGGGATATTCCCATCTAACTTGTTACAT
GAAATTTCCAGCACATTGAGGCTTCCCAAGCTCTGTAGCCACGGGGGAATTACGCCGATGAGCAG
ACAGTTTGTTAGGATGAGCACCTGCATTGTCTCACCGCCGCGGAAATTCCTGGTCAGCACCAAGCT
TGTCAGGTTGGGCAGGTGTTGCAAGACCTGCAATGTCTATGCCAGGTTGTGAAACTATTCCTGGTC
AGCGAGAGGTACGACAGGGATCTCAACTCCTTGAAGCTCTCCGGTATCCCCCCCCACGAGCTTGTT
CCTTGTAAGATTCAGTGTCCCCAACTTGGTGCACACACTGATGCCAGGAGCTATAGCGCCACTCAG
ATAGTTGGTTCCAACATCAAAAGTATTAAGCTTCGGCAGCAAGCTGGAGTCGATAGCAATCTCACT
AGAGAGCGAGTTGTTCCTCAAGCTGATTACCCTCAGCAGTGGAGAACTTTACAGGGAAGCGGGCA
ACTCACCATCCAGCCTATTGGTGGCCAGGTTTACGAACTCTAGCCTCCTCATCTCTCCAAAGACAT
CAGGGATGGAGCCTGTGAACTTGTTATAGGACAAGTCAAGTTGCATGAT

CML121

TAGTCAAATATATATGAAAAAAAACTTTAGTACAAATATATATATGAAAAAATGGGATGGAGAAA
CTGGTAATTCGAGTTACTGAACTCTTTGTTGTTTTGGAATAGCAGCACCAGGCAAGAGTTGGAGAA
CTAGGAGTCTTCGGTTGTCGCTACGGCCTTCGGATTGCTCCTGTATTCTTCAATGGACAATCCTTGA
CACGGTCACATAAGCACAACAAGAGGAGAACCCCGACCACAGTTCCCAGTCCAAGGGCGACGATG
GCGGATTTGCTTTTATTGCTGAACAATGGGAACCCTCTGTGGGTGACCGCTTGCTCGAGCCATTATT
TGAATTCAAGCTCCTCATCTGTGTGAAGCTAACAAGTAGCTTACCGCTGAAAGAATTGTCTGAGAG
ATCAATGTTGAAGCAGTTGTCTAGCTTCCCCAGCCATGGCAGGATATTCCCATCTAACTTGTTACAT
GAAATTTCCAGCACATTGAGGCTTCCCAAGCTCTGCAGCCACGGGGGAATTACGCCGATGAGTAG
ATAGTTTGTCAGGATGAGCACCTGCATTGTCTCACCGCCGCGGAAATTCCTGGTCAGCACCAAGCT
TGTCAGGTTGGGCAGGTGTTGCAAGACCTGCAATGTCTATGCCAGGTTGTGAAACTATTCCTGGTC
AGCGAGAGGTACGACAGGGATCTCAACTCCTTGAAGCTCTCCGGTATCCCCCACGAGCTTGTTCCT
TGTAAGATTTAGTGTCCTCAACTTGGTGCACACACTGATGCCAGGAGCTATAGCACCACTCAGATA
GTTGGTTCCAACATCAAAAGTATTAAGCTTCGGCAGCAAGCTGGAGTCGATAGCAATCTCACTAGA
GAGCGAGTTGTTCCTCAAGCTGATTACCCTCAGCAGTGGAGAACTTTACAGGGAAGCGGGCAACT
CACCATCCAGCCTATTGGTGGCCAGGTTTACGGACTCTAGCCTCCTCATCTCTCCAAAGACATCAG
GGATGGAGCCTGTGAACTTGTTATAGGACAAGTCAAGTTGCATGAT

Xun971

TAGTCAAATATATATGAAAAAAACTTTAGTACAAATATATATATGAAAAAATGGGATGGAGAAAC
TGGTAATTCGAGTTACTGAACTCTTTGTTGTTTTGGAATAGCAGCACCAGGCAAGAGTTGGAGAAC
TAGGAGTCTTCGGCTGTCGCTACGGCCTTCGGATTGCTCCTGTATTCTTCAATGGACAATCCTTGAC
ACGGTCACATAAGCACAACAAGAGGAGAACCCCGACCACAGTTCCCAGTCCAAGGGCGACGATG
GCGGATTTGCTTTTATTGCTGAACAATGGGAACCCTCTGTGGGTGACCGCTTGCTCGAGCCATTATT
TGAATTCAAGCTCCTCATCTGTGTGAAGCTGACAGGTAGCTTACCGCTGAAAGAATTGTTTGAGAG
GTCAATGTTGAAGCAGTTGTCTAGCTTCCCTAGCCATGGCGGGATATTCCCATCTAACTTGTTACAT
GAAATTTCCAGCACATTGAGGCTTCCCAAGCTCTGTAGCCACGGGGGAATTACGCCGATGAGCAG
ACAGTTTGTTAGGATGAGCACCTGCATTGTCTCACCGCCGCGGAAATTCCTGGTCAGCACCAAGCT
TGTCAGGTTGGGCAGGTGTTGCAAGACCTGCAATGTCTATGCCAGGTTGTGAAACTATTCCTGGTC
AGCGAGAGGTACGACAGGGATCTCAACTCCTTGAAGCTCTCCGGTATCCCCCCCCACGAGCTTGTT
CCTTGTAAGATTCAGTGTCCTCAACTTGGTGCACACACTGATGCCAGGAGCTATAGCGCCACTCAG
ATAGTTGGTTCCAACATCAAAAGTATTAAGCTTCGGCAGCAAGCTGGAGTCGATAGCAATCTCACC
AGAGAGCGAGTTGTTCCTCAAGCTGATTACCCTCAGCAGTGGAGAACTTTACAGGGAAGCGGGCA
ACTCACCATCCAGCCTATTGGTGGCCAGGTTTACGAACTCTAGCCTCCTCATCTCTCCAAAGACAT
CAGGGATGGAGCCTGTGAACTTGTTATAGGACAAGTCAAGTTGCATGAT

CIMBL87

TAGTCAAATATATATGAAAAAAAACTTTAGTACAAATATATATATGAAAAAATGGGATGGAGAAA
CTGGTAATTCGAGTTACTGAACTCTTTGTTGTTTTGGAATAGCAGCACCAGGCAAGAGTTGGAGAA
CTAGGAGTCTTCGGTTGTCGCTACGGCCTTCGGATTGCTCCTGTATTCTTCAATGGACAATCCTTGA
CACGGTCACATAAGCACAACAAGAGGAGAACCCCGACCACAGTTCCCAGTCCAAGGGCGACGATG
GCGGATTTGCTTTTATTGCTGAACAATGGGAACCCTCTGTGGGTGACCGCTTGCTCGAGCCATTATT

TGAATTCAAGCTCCTCATCTGTGTGAAGCTAACAGGTAGCTTACCGCTGAAAGAATTGTTTGAGAG
ATCAATGTTGAAGCAGTTGTCTAGCTTCCCCAGCCATGGCAGGATATTCCCATCTAACTTGTTACAT
GAAATTTCCAGCACATTGAGGCTTCCCAAGCTCTGCAGCCACGGGGGAATTACGCCGATGAGTAG
ATAGTTTGTCAGGATGAGCACCTGCATTGTCTCACCGCCGCGGAAATTCCTGGTCAGCACCAAGCT
TGTCAGGTTGGGCAGGTGTTGCAAGACCTGCAATGTCTATGCCAGGTTGTGAAACTATTCCTGGTC
AGCGAGAGGTACGACAGGGATCTCAACTCCTTGAAGCTCTCCGGTATCCCCCACGAGCTTGTTCCT
TGTAAGATTTAGTGTCCTCAACTTGGTGCACACACTGATGCCAGGAGCTATAGCACCACTCAGATA
GTTGGTTCCAACATCAAAAGTATTAAGCTTCGGCAGCAAGCTGGAGTCGATAGCAATCTCACTAGA
GAGCGAGTTGTTCCTCAAGCTGATTACCCTCAGCAGTGGAGAACTTTACAGGGAAGCGGGCAACT
CACCATCCAGCCTATTGGTGGCCAGGTTTACGGACTCTAGCCTCCTCATCTCTCCAAAGACATCAG
GGATGGAGCCTGTGAACTTGTTATAGGACAAGTCAAGTTGCATGAT
FCD0602
TAGTCAAATATATATGAAAAAAACTTCAGTACAAATATATATATGAAAAAATGGGATGGAGAAAC
TGGTAATTCGAGTTACTGAACTCTTTGTTGTTTTGGAATAGCAGCACCAGGCAAGAGTTGGAGAAC
TAGGAGTCTTCGGTTGTCGCTACGGCCTTCGGATTGCTCCTGTATTCTTCAATGGACAATCCTTGAC
ACGATCACATAAGCACAACAAGAGGAGAAACCCGACCACAGTTCCTAGTCCAAGGGCAACGATG
GCGGCTTTGCTTTTATTGCTGAACAATGGGAGCCCTCTGTGGGTGATCGCTTGCTCGAGCCATTATT
TGAATTCAAGCTCCTCATCTGTGTGAAGCTGACAGGTAGCTTACCGCTGAAAGAATTGTTTGAGAG
GTCAATGTTGAAGCGGTTGTCTAGCTTCCCCAGCCATGGCGGGATATTCCCATCTAACTTGTTACAT
GAAATTTCCAGCACATTGAGGCTTCCCAACCTCTGCAGCCACGGGGGAATTACGCCGATGAGTAG
ACAGTTTGTCAGGATGAGCACCTGCATTGTCTCACCACCGCGGAAATTCCTGGTCAGCACCAAGCT
TGTCAGGTTGGGCAGGTGTTGCAAGACCTGCAATGTCTATGCCAGGTTGTGAAGCTATTCCTGGTC
AGCGAGAGGTACGACACGGATCTCAACTCCTTGAAGCTCTCTGGTATCTCCCCCATGAGCTTGTTC
CTTGTAAGATTCAGTGTCCTCAACTTGGTGCACACACTGATGCTAGAAGCTATAGCGCCACTCAGA
TAGTTGGTTCCAACATCAAAAGTATTAAGCTTCGGCAGCAAGCTGGAGTCGATAGCAATCTCACCA
GAGAGCGAGTTGTTCCTCAAGCTGATTACCCTCAGAAGTGGACAACTTTACAGGGAATCGGGCAA
CTCACCATCCAGCCTATTGGTGGCCAGGTTTACGGACTCTAGCCTCCTCATCTCTCCAAAGACATC
AAGTATGGAGCCTGTGAACTTATTATAGGACAAGTCAAGTTGCATGAT
K22
TAGTCAAATATATATGAAAAAAACTTTAGTACAAATATATATATGAAAAAATGGGATGGAGAAAC
TGGTAATTCGAGTTACTGAACTCTTTGTTGTTTTGGAATAGCAGCACCAGGCAAGAGTTGGAGAAC
TAGGAGTCTTCGGTTGTCGCTACGGCCTTCGGATTGCTCCTGTATTCTTCAATGGACGATCCTTGAC
ACGATCACATAAGCACAACAAGAGGAGAAACCCGACCACAGTTCCTAGTCCAAGGGCAACGATG
GCGGCTTTGCTTTTATTGCTGAACAATGGGAGCCCTCTGTGGGTGATCGCTTGCTCGAGCCATTATT
TGAATTCAAGCTCCTCATCTGTGTGAAGCTGACAGGTAGCTTACCGCTGAAAGAATTGTTTGAGAG
GTCAATGTTGAAGCGGTTGTCTAGCTTCCCCAGCCATGGCGGGATATTCCCATCTAACTTGTTACAT
GAAATTTCCAGCACATTGAGGCTTCCCAACCTCTGCAGCCACGGGGGAATTACGCCGATGAGTAG
ACAGTTTGTCAGGATGAGCACCTGCATTGTCTCACCACCGCGGAAATTCCTGGTCAGCACCAAGCT
TGTCAGGTTGGGCAGGTGTTGCAAGACCTGCAATGTCTATGCCAGGTTGTGAAACTATTCCTGGTC
AGCGAGAGGTACGACAGGGATCTCAACTCCTTGAAGCTCTCCGGTATCCCCCACGAGCTTGTTCCT
TGTAAGATTTAGTGTCCTCAACTTGGTGCACACACTGATGCCAGGAGCTATAGCACCACTCAGATA
GTTGGTTCCAACATCAAAAGTATTAAGCTTCGGCAGCAAGCTGGAGTCGATAGCAATCTCACTAGA
GAGCGAGTTGTTCCTCAAGCTGATTACCCTCAGCAGTGGAGAACTTTACAGGGAAGCGGGCAACT
CACCATCCAGCCTATTGGTGGCCAGGTTTACGGACTCTAGCCTCCTCATCTCTCCAAAGACATCAG
GGATGGAGCCTGTGAACTTGTTATAGGACAAGTCAAGTTGCATGAT
Nan21-3
TAGTCAAATATATATGAAAAAAACTTCAGTACAAATATATATATGAAAAAATGGGATGGAGAAAC
TGGTAATTCGAGTTACTGAACTCTTTGTTGTTTTGGAATAGCAGCACCAGGCAAGAGTTGGAGAAC
TAGGAGTCTTCGGTTGTCGCTACGGCCTTCGGATTGCTCCTGTATTCTTCAATGGACAATCCTTGAC
ACGATCACATAAGCACAACAAGAGGAGAAACCCGACCACAGTTCCTAGTCCAAGGGCAACGATG
GCGGCTTTGCTTTTATTGCTGAACAATGGGAGCCCTCTGTGGGTGATCGCTTGCTCGAGCCATTATT
TGAATTCAAGCTCCTCATCTGTGTGAAGCTGACAGGTAGCTTACCGCTGAAAGAATTGTTTGAGAG
GTCAATGTTGAAGCGGTTGTCTAGCTTCCCCAGCCATGGCGGGATATTCCCATCTAACTTGTTACAT
GAAATTTCCGGCACATTGAGGCTTCCCAACCTCTGCAGCCACGGGGGAATTACGCCGATGAGTAG
ACAGTTTGTCAGGATGAGCACCTGCATTGTCTCACCACCGCGGAAATTCCTGGTCAGCACCAAGCT
TGTCAGGTTGGCAGGTGTTGCAAGACCTGCAATGTCTATGCCAGGTTGTGAAGCTATTCCTGGTCA
GCGAGAGGTACGACACGGATCTCAACTCCTTGAAGCTCTCTGGTATCTCCCCCATGAGCTTGTTCC
TTGTAAGATTCAGTGTCCTCAACTTGGTGCACACACTGATGCTAGAAGCTATAGCGCCACTCAGAT
AGTTGGTTCCAACATCAAAAGTATTAAGCTTCGGCAGCAAGCTGGAGTCGATAGCAATCTCACCA
GAGAGCGAGTTGTTCCTCAAGCTGGTTACCCTCAGAAGTGGACAACTTTACAGGGAATCGGGCAA

CTCACCATCCAGCCTATTGGTGGCCAGGTTTACGGACTCTAGCCTCCTCATCTCTCCAAAGACATC
AAGTATGGAGCCTGTGAACTTATTATAGGACAAGTCAAGTTGCATGAT

CIMBL88

TAGTCAAATATATATGAAAAAAACTTTAGTACAAATATATATATGAAAAAATGGGATGGAGAAAC
TGGTAATTCGAGTTACTGAACTCTTTGTTGTTTTGGAATAGCAGCACCAGGCAAGAGTTGGAGAAC
TAGGAGTCTTCGGTTGTCGCTACGGCCTTCGGATTGCTCCTGTATTCTTCAATGGACAATCCTTGAC
ACGATCACATAAGCACAACAAGAGGAGAAACCCGACCACAGTTCCTAGTCCAAGGGCAACGATG
GCGGCTTTGCTTTTATTGCTGAACAATGGGAGCCCTCTGTGGGTGATCGCTTGCTCGAGCCATTATT
TGAATTCAAGCTCCTCATCTGTGTGAAGCTGACAGGTAGCTTACCGCTGAAAGAATTGTTTGAGAG
GTCAATGTTGAAGCGGTTGTCTAGCTTCCCCAGCCATGGCGGGATATTCCCATCTAACTTGTTACAT
GAAATTTCCAGCACATTGAGGCTTCCCAACCTCTGCAGCCACGGGGGAATTACGCCGATGAGTAG
ACAGTTTGTCAGGATGAGCACCTGCATTGTCTCACCACCGCGGAAATTCCTGGTCAGCACCAAGCT
TGTCAGGTTGGGCAGGTGTTGCAAGACCTGCAATGTCTATGCCAGGTTGTGAAGCTATTCCTGGTC
AGCGAGAGGTACGACACGGATCTCAACTCCTTGAAGCTCTCTGGTATCTCCCCCATGAGCTTGTTC
CTTGTAAGATTCAGTGTCCTCAACTTGGTGCACACACTGATGCTAGAAGCTATAGCGCCACTCAGA
TAGTTGGTTCCAACATCAAAAGTATTAAGCTTCGGCAGCAAGCTGGAGTCGATAGCAATCTCACCA
GAGAGCGAGTTGTTCCTCAAGCTGATTACCCTCAGAAGTGGACAACTTTACAGGGAATCGGGCAA
CTCACCATCCAGCCTATTGGTGGCCAGGTTTACGGACTCTAGCCTCCTCATCTCTCCAAAGACATC
AAGTATGGAGCCTGTGAACTTATTATAGGACAAGTCAAGTTGCATGAT

3H-2

TAGTCAAATATATATGAAAAAAACTTTAGTACAAATATATATATGAAAAAATGGGATGGAAAAAC
CGGTAATTCGAGTTACTGAACTCTTTGTTGTTTGGAATAGCAGCACCAGGCAAGAGTTGGAGAACT
AGGAGTCTTCGGCTGTCGCTACGGCCTTCGGATTGCTCCTGTATTCTTCAATGGACAATCCTTGACA
CGGTCACATAAGCACAACAAGAGGAGAACCCCGACCACAGTTCCCAGTCCAAGGGCGACGATGGC
GGATTTGCTTTTATTGCTGAACAATGGGAACCCTCTGTGGGTGACCGCTTGCTCGAGCCATTGTTTG
AATTCAAGCTCCTCATCTGTGTGAAGCTAACAGGTAGCTTACCGCTGAAAGAATTGTTTGAGAGAT
CAATGTTGAAGCAGTTGTCTAGCTTCCCCAGCCATGGCAGGATATTCCCATCTAACTTGTTACATG
AAATTTCCAGCACATTGAGGCTTCCCAAGCTCTGCAGCCACGGGGGAATTACGCCGATGAGTAGA
TAGTTTGTCAGGATGAGCACCTGCATTGTCTCACCACCGCGGAAATTCCTGGTCAGCACCAAGCTT
GTCAGGTTGGGCAGGTGTTGCAAGACCTGCAATGTCTATGCCAGGTTGTGAAACTATTCCTGGTCA
GCGAGAGGTACGACAGGGATCTCAACTCCTTGAAGCTCTCCGGTATCCCCCACGAGCTTGTTCCTT
GTAAGATTTAGTGTCCTCAACTTGGTGCACACACTGATGCCAGGAGCTATAGCACCACTCAGATAG
TTGGTTCCAACATCAAAAGTATTAAGCTTCGGCAGCAAGCTGGAGTCGATAGCAATCTCACTAGAG
AGCGAGTTGTTCCTCAAGCTGATTACCCTCAGCAGTGGAGAACTTTACAGGGAAGCGGGCAACTC
ACCATCCAGCCTATTGGTGGCCAGGTTTACGGACTCTAGCCTCCTCATCTCTCCAAAGACATCAGG
GATGGAGCCTGTGAACTTGTTATAGGACAAGTCAAGTTGCATGAT

M153

TAGTCAAATATATATGAAAAAAACTTTAGTACAAATATATATATGAAAAAATGGGATGGAGAAAC
TGGTAATTCGAGTTACTGAACTCTTTGTTGTTTTGGAATAGCAGCACCAGGCAAGAGTTGGAGAAC
TAGGAGTCTTCGGCTGTCGCTACGGCCTTCGGATTGCTCCTGTATTCTTCAATGGACAATCCTTGAC
ACGGTCACATAAGCACAACAAGAGGAGAACCCCGACCACAGTTCCCAGTCCAAGGGCGACGATG
GCGGATTTGCTTTTATTGCTGAACAATGGGAACCCTCTGTGGGTGACCGCTTGCTCGAGCCATTATT
TGAATTCAAGCTCCTCATCTGTGTGAAGCTGACAGGTAGCTTACCGCTGAAAGAATTGTTTGAGAG
GTCAATGTTGAAGCAGTTGTCTAGCTTCCCTAGCCATGGCGGGATATTCCCATCTAACTTGTTACAT
GAAATTTCCAGCACATTGAGGCTTCCCAAGCTCTGTAGCCACGGGGGAATTACGCCGATGAGCAG
ACAGTTTGTTAGGATGAGCACCTGCATTGTCTCACCGCCGCGGAAATTCCTGGTCAGCACCAAGCT
TGTCAGGTTGGGCAGGTGTTGCAAGACCTGCAATGTCTATGCCAGGTTGTGAAACTATTCCTGGTC
AGCGAGAGGTACGACAGGGATCTCAACTCCTTGAAGCTCTCCGGTATCCCCCCCCACGAGCTTGTT
CCTTGTAAGATTCAGTGTCCTCAACTTGGTGCACACACTGATGCCAGGAGCTATAGCGCCACTCAG
ATAGTTGGTTCCAACATCAAAGGTATTAAGCTTCGGCAGCAAGCTGGAGTCGATAGCAATCTCACT
AGAGAGCGAGTTGTTCCTCAAGCTGATTACCCTCAGCAGTGGAGAACTTTACAGGGAAGCGGGCA
ACTCACCATCCAGCCTATTGGTGGCCAGGTTTACGAACTCTAGCCTCCTCATCTCTCCAAAGACAT
CAGGGATGGAGCCTGTGAACTTGTTATAGGACAAGTCAAGTTGCATGAT

# Smaller starch granules size

IRF314

TAGTCAAATATATATGAAAAAAACTTTAGTACAAATATATATATGAAAAAATGGGATGGAGAAAC
TGGTAATTCGAGTTACTGAACTCTTTGTTGTTTTGGAATAGCAGCACCAGGCAAGAGTTGGAGAAC
TAGGAGTCTTCGGCTGTCGCTACGGCCTTCGGATTGCTCCTGTATTCTTCAATGGACAATCCTTGAC

ACGGTCACATAAGCACAACAAGAGGAGAACCCCGACCACAGTTCCCAGTCCAAGGGCGACGATG
GCGGATTTGCTTTTATTGCTGAACAATGGGAACCCTCTGTGGGTGACCGCTTGCTCGAGCCATTATT
TGAATTCAAGCTCCTCATCTGTGTGAAGCTGACAGGTAGCTTACCGCTGAAAGAATTGTTTGAGAG
GTCAATGTTGAAGCAGTTGTCTAGCTTCCCTAGCCATGGCGGGATATTCCCATCTAACTTGTTACAT
GAAATTTCCAGCACATTGAGGCTTCCCAAGCTCTGTAGCCATGGGGGAATTACGCCGATGAGCAG
ACAGTTTGTTAGGATGAGCACCTGCATTGTCTCACCGCCGCGGAAATTCCTGGTCAGCACCAAGCT
TGTCAGGTTGGGCAGGTGTTGCAAGACCTGCAATGTCTATGCCAGGTTGTGAAACTATTCCTGGTC
AGCGAGAGGTACGACAGGGATCTCAACTCCTTGAAGCTCTCCGGTATCCCCCCCCACGAGCTTGTT
CCTTGTAAGATTCAGTGTCCTCAACTTGGTGCACACACTGATGCCAGGAGCTATAGCGCCACTCAG
ATAGTTGGTTCCAACATCAAAAGTATTAAGCTTCGGCAGCAAGCTGGAGTCGATAGCAATCTCACT
AGAGAGCGAGTTGTTCCTCAAGCTGATTACCCTCAGCAGTGGAGAACTTTACAGGGAAGCGGGCA
ACTCACCATCCAGCCTATTGGTGGCCAGGTTTACGAACTCTAGCCTCCTCATCTCTCCAAAGACAT
CAGGGATGGAGCCTGTGAACTTGTTATAGGACAAGTCAAGTTGCATGAT
CML423
TAGTCAAATATATATGAAAAAAACTTTAGTACAAATATATATATGAAAAAATGGGATGGAGAAAC
TGGTAATTCGAGTTACTGAACTCTTTGTTGTTTTGGAATAGCAGCACCAGGCAAGAGTTGGAGAAC
TAGGAGTCTTCGGCTGTCGCTACGGCCTTCGGATTGCTCCTGTATTCTTCAATGGACAATCCTTGAC
ACGGTCACATAAGCACAACAAGAGGAGAACCCCGACCACAGTTCCCAGTCCAAGGGCGACGATG
GCGGATTTGCTTTTATTGCTGAACGATGGGAACCCTCTGTGGGTGACCGCTTGCTCGAGCCATTATT
TGAATTCAAGCTCCTCATCTGTGTGAAGCTGACAGGTAGCTTACCGCTGAAAGAATTGTTTGAGAG
GTCAATGTTGAAGCAGTTGTCTAGCTTCCCTAGCCATGGCGGGATATTCCCATCTAACTTGTTACAT
GAAATTTCCAGCACATTGAGGCTTCCCAAGCTCTGTAGCCACGGGGAAATTACGCCGATGAGCAG
ACAGTTTGTTAGGATGAGCACCTGCATTGTCTCACCGCCGCGGAAATTCCTGGTCAGCACCAAGCT
TGTCAGGTTGGGCAGGTGTTGCAAGACCTGCAATGTCTATGCCAGGTTGTGAAACTATTCCTGGTC
AGCGAGAGGTACGACAGGGATCTCAACTCCTTGAAGCTCTCCGGTATCCCCCCCCACGAGCTTGTT
CCTTGTAAGATTCAGTGTCCTCAACTTGGTGCACACACTGATGCCAGGAGCTATAGCGCCACTCAG
ATAGTTGGTTCCAACATCAAAAGTATTAAGCTTCGGCAGCAAGCTGGAGTCGATAGCAATCTCACC
AGAGAGCGAGTTGTTCCTCAAGCTGATTACCCTCAGCAGTGGAGAACTTTACAGGGAAGCGGGCA
ACTCACCATCCAGCCTATTGGTGGCCAGGTTTACGAACTCCAGCCTCCTCATCTCTCCGAAGACAT
CAGGGATGGAGCCTGTGAACTTGTTATAGGACAAGTCAAGTTGCATGAT
Gy220
TAGTCAAATATATATGAAAAAAAACTTTAGTACAAATATATATATGAAAAAATGGGATGGAGAAA
CTGGTAATTCGAGTTACTGAACTCTTTGTTGTTTTGGAATAGCAGCATCAGGCAAGAGTTGGAGAA
CTAGGAGTCTTCGGCTGTCGCTACGGCCTTCGGATTGCTCCTGTATTCTTCAATGGACAATCCTTGA
CACGGTCACATAAGCACAACAAGAGGAGAACCCCGACCACAGTTCCCAGTCCAAGGGCGATGATG
GCGGATTTGCTTTTATTGCTGAACAATGCGAACCCTCTGTGGGTGACCGCTTGCTCGAGCCATTATT
TGAATTCAAGCTCCTCATCTGTGTGAAGCTGACAAGTAGCATACCGCTGAAAGAATTGTTTGAGAG
GTCAATGTTGAAGCAGTTGTCTAGCTTCCCCATCCATGGCGGGATATTCCCATCTAACTTGTTACAT
GAAATTTCCAGCACATTGAGGCTTCCCAAGCTCTGCAGCCACGGGGGAATTACGCCGATGAGTAG
ACAGTTTGTCAAGATGAGCACCTGCATTGTCTCACCGCTGCGGAAATTCCTGGTCAGCACCAAGCT
TGTCAGGTTGGGCAGGTGTTGCAAGACCTGCAATGTCTATGCCAGGTTGTGAAACTATTCCTGGTC
AGCGAAAGGTACGACAGGATCTCAACTCCTTGAAGCTCTCCGGTATCCCCCCAACGAGCTTGTTCC
TTGTAAGATTCAGTGTCCTCAACTTGGTGCACACACTGATGCCAGTAGCTATAGCGCCACTCGGAT
AGTTGGTTCCAACATCAAAAGTATTAAGCTTCGGCAGCAAGCTGGAGTCGATAGCAATCTCACTAG
AGAGCGAGTTGTTCCTCAAGGTGATTACCCTCAGCAGTGGAGAACTTTACAGGGAAGCGGGCAAC
TCACCATCCAGCCTATTGGTGGCCAGGTTTACGGACTCTAGCCTCCTCATCTCTCCAAAGACATCA
GGGATGGAGCCTGTGAACTTGTTATAGGACAAGTCAAGTTGCATGAT
GEMS20
TAGTCAAATATATATGAAAAAAACTTTAGTACAAATATATATATGAAAAAATGGGATGGAGAAAC
CGGTAATTCGAGTTACTGAACTCTTTGTTGTTTTGGAATAGCAGCACCAGGCAAGAGTTGGAGAAC
TAGGAGTCTTCGGCTGTCGCTACGGCCTTCGGATTGCTCCTGTATTCTTCAATGGACAATCCTTGAC
ACGATCACATAAGCACAACAAGAGGAGAAACCCGACCACAGTTCCTAGTCCAAGGGCAACGATG
GCGGCTTTGCTTTTATTGCTGAACAATGGGAGCCCTCTGTGGGTGATCGCTTGCTCGAGCCATTATT
TGAATTCAAGCTCCTCATCTGTGTGAAGCTGACAGGTAGCTTACCGCTGAAAGAATTGTTTGAGAG
GTCAATGTTGAAGCGGTTGTCTAGCTTCCCCAGCCATGGCGGGATATTCCCATCTAACTTGTTACAT
GAAATTTCCAGCACATTGAGGCTTCCCAACCTCTGCAGCCACGGGGGAATTACGCCGATGAGTAG
ACAGTTTGTCAGGATGAGCACCTGCATTGTCTCACCACCGCGGAAATTCCTGGTCAGCACCAAGCT
TGTCAGGTTGGGCAGGTGTTGCAAGACCTGCAATGTCTATGCCAGGTTGTGAAGCTATTCCTGGTC
AGCGAGAGGTACGACACGGATCTCAACTCCTTGAAGCTCTCTGGTATCTCCCCCATGAGCTTGTTC
CTTGTAAGATTCAGTGTCCTCAACTTGGTGCACACACTGATGCTAGAAGCTATAGCGCCACTCAGA

TAGTTGGTTCCAACATCAAAAGTATTAAGCTTCGGCAGCAAGCTGGAGTCGATAGCAATCTCACCA
GAGAGCGAGTTGTTCCTCAAGCTGATTACCCTCAGAAGTGGACAACTTTACAGGGAATCGGGCAA
CTCACCATCCAGCCTATTGGTGGCCAGGTTTACGGACTCTAGCCTCCTCATCTCTCCAAAGACATTA
AGTATGGAGCCTGTGAACTTATTATAGGACAAGTCAAGTTGCATGAT

GEMS41

TAGTCAAATATATATGAAAAAAACTTTAGTACAAATATATATATGAAAAAATGGGATGGAGAAAC
TGGTAATTCGAGTTACTGAACTCTTTGTTGCTTTGGAATAGCAGCACCAGGCAAGAGTTGGAGAAC
TAGGAGTCTTCGGTTGTCGCTACGGCCTTCGGATTGCTCCTGTATTCTTCAATGGACAATCCTTGAC
ACGATCACATAAGCACAACAAGAGGAGAAACCCGACCACAGTTCCTAGTCCAAGGGCAACGATG
GCGGCTTTGCTTTTATTGCTGAACAATGGGAGCCCTCTGTGGGTGATCGCTTGCTCGAGCCATTATT
TGAATTCAAGCTCCTCATCTGTGTGAAGCTGACAGGTAGCTTACCGCTGAAAGAATTGTTTGAGAG
GTCAATGTTGAAGCGGTTGTCTAGCTTCCCCAGCCATGGCGGGATATTCCCATCTAACTTGTTACAT
GAAATTTCCAGCACATTGAGGCTTCCCAACCTCTGCAGCCACGGGGGAATTACGCCGATGAGTAG
ACAGTTTGTCAGGATGAGCACCTGCATTGTCTCACCACCGCGGAAATTCCTGGTCAGCACCAAGCT
TGTCAGGTTGGGCAGGTGTTGCAAGACCTGCAATGTCTATGCCAGGTTGTGAAGCTATTCCTGGTC
AGCGAGAGGTACGACACGGATCTCAACTCCTTGAAGCTCTCTGGTATCTCCCCCATGAGCTTGTTC
CTTGTAAGATTCAGTGTCCTCAACTTGGTGCACACACTGATGCTAGAAGCTATAGCGCCACTCAGA
TAGTTGGTTCCAACATCAAAAGTATTAAGCTTCGGCAGCAAGCTGGAGTCGATAGCAATCTCACCA
GAGAGCGAGTTGTTCCTCAAGCTGATTACCCTCAGAAGTGGACAACTTTACAGGGAATCGGGCAA
CTCACCATCCAGCCTATTGGTGGCCAGGTTTACGGACTCTAGCCTCCTCATCTCTCCAAAGACATC
AAGTATGGAGCCTGTGAACTTATTATAGGACAAGTCAAGTTGCATGAT

B111

TAGTCAAATATATATGAAAAAAACTTTAGTACAAATATATATATGAAAAAATGGGATGGAGAAAC
TGGTAATTCGAGTTACTGAACTCTTTGTTGTTCTGGAATAGCAGCACCAGGCAAGAGTTGGAGAAC
TAGGAGTCTTCGGCTGTCGCTACGGCCTTCGGATTGCTCCTGTATTCTTCAATGGACAATCCTTGAC
ACGGTCACATAAGCACAACAAGAGGAGAACCCCGACCACAGTTCCCAGTCCAAGGGCGACGATG
GCGGATTTGCTTTTATTGCTGAACAATGGGAACCCTCTGTGGGTGACCGCTTGCTCGAGCCATTATT
TGAATTCAAGCTCCTCATCTGTGTGAAGCTGACAGGTAGCTTACCGCTGAAAGAATTGTTTGAGAG
GTCAATGTTGAAGCAGTTGTCTAGCTTCCCTAGCCATGGCGGGATATTCCCATCTAACTTGTTACAT
GAAATTTCCAGCACATTGAGGCTTCCCAAGCTCTGTAGCCACGGGGGAATTACGCCGATGAGCAG
ACAGTTTGTTAGGATGAGCACCTGCATTGTCTCACCGCCGCGGAAATTCCTGGTCAGCACCAAGCT
TGTCAGGTTGGGCAGGTGTTGCAAGACCTGCAATGTCTATGCCAGGTTGTGAAACTATTCCTGGTC
AGCGAGAGGTACGACAGGGATCTCAACTCCTTGAAGCTCTCCGGTATCCCCCCCCACGAGCTTGTT
CCTTGTAAGATTCAGTGCCCTCAACTTGGTGCACACACTGATGCCAGGAGCTATAGCGCCACTCAG
ATAGTTGGTTCCAACATCAAAAGTATTAAGCTTCGGCAGCAAGCTGGAGTCGATAGCAATCTCACT
AGAGAGCGAGTTGTTCCTCAAGCTGATTACCCTCAGCAGTGGAGAACTTTACAGGGAAGCGGGCA
ACTCACCATCCAGCCTATTGGTGGCCAGGTTTACGAACTCTAGCCTCCTCATCTCTCCAAAGACAT
CAGGGATGGAGCCTGTGAACTTGTTATAGGACAAGTCAAGTTGCATGAT

Gy237

TAGTCAAATATATATGAAAAAAAATTAGTACAAATATATATATGAAAAAATGGGATGGAGAAACT
GGTAATTCGAGTTACTGAACTCTTTGTTGTTTTGGAAAAGCAGCACCAGACAAGAGTTGGAGAACT
AGGAGTCTTCGGCTGTCGCTACGGCCTTCGGATTGCTCCTGTATTCTTCAATGGACAATCCTTAACA
CGGTCACATAAGCACAACAAGAGGAGAACCCTGACCACAGTTCCTAGTCCAAGGGCGACGATGGC
GGATTTGCTTTTATTGCTGAACAATGGGAACCCTCTGTGGGTGACTGCTTGCTCGAGCCATTATTTG
AATTCAAGCTCCTCATCTGTGTGAAGCTGACAGGTAGCTTACCGCTGAAAGAATTGTTTGAGAGGT
CAATGTTGAAGCAGTTGTCTAGCTTCCCCAGCCATGGCGGGATATTCCCATCTAACTTGTTACATG
AAATTTCCAGCACATTGAGGCTTCCCAAGCTCTGCAGCCACGGGGGAATTACGCCGATGAGTAGA
CAGTTTGTCAGGATGAGCACCTGCATTGTCTCACCGCCGCGGAAATTCTTGGTCAGCACCAAGCTT
GTCAGGTTGGGCAGGTGTTGCAAGACCTGCAATGTCTATGCCAGGTTGTGAAACTATTCCTGGTCA
GCGAGAGGTACGACAGGGATCTCAACTCCTTGAAGCTCTCCGGTATCCCCCCCCACGAGCTTGTTC
CTTGTAAGATTCAGTGTCCTCAACTTGGTGCACACACTGATGCCAGGAGCTATAGCGCCACTCAGA
TAGTTGGTTCCAACATCAAAAGTATTAAGCTTCGGCAGCAAGCTGGAGTCGATAGCAATCTCACCA
GAGAGCGAGTAGTTCCTCAAGCTGATTACCCTCAGCAGTGGACAACTTTACAGGGAAGCGGGCAA
CTCACCGTCCAGCCTATTGGTGGCCAGGTTTACGGACTCTAGCCTCCTCATCTCTCCAAAGACATC
AGGGATGGAGCCTGTGAACTTGTTATAGGACAAGTCAAGTTGCATGAT

CIMBL 157

TAGTCAAATATATATGAAAAAAAACTTTAGTACAAATATATATATGAAAAAATGGGATGGAGAAA
CTGGTAATTCGAGTTACTGAGCTCTTTGTTGTTTTGGAATAGCAGCACCAGGCAAGAGTTGGAGAA
CTAGGAGTCTTCGGTTGTCGCTACGGCCTTCGGATTGCTCCTGTATTCTTCAATGGACAATCCTTGA
CACGGTCACATAAGCACAACAAGAGGAGAACCCTGACCACAGTTCCCAGTCCAAGGGCGACGATG

GCGGATTTGCTTTTATTGCTGAACAATGGGAACCCTCTGTGGGTGACCGCTTGCTCGAGCCATTATT
TGAATTCAAGCTCCTCACCTGTGTGAAGCTAACAGGTAGCTTACCGCTGAAAGAATTGTTTGAGAG
ATCAATGTTGAAGCAGTTGTCTAGCTTCCCCAGCCATGGCAGGATATTCCCATCTAACTTGTTACAT
GAAATTTCCAGCACATTGAGGCTTCCCAAGCTCTGCAGCCACGGGGGAATTACGCCGATGAGTAG
ATAGTTTGTCAGGATGAGCACCTGCATTGTCTCACCGCCGCGGAAATTCCTGGTCAGCACCAAGCT
TGTCAGGTTGGGCAGGTGTTGCAAGACCTGCAATGTCTATGCCAGGTTGTGAAACTATTCCTGGTC
AGCGAGAGGTACGACAGGGATCTCAACTCCTTGAAGCTCTCCGGTATCCCCCACGAGCTTGTTCCT
TGTAAGATTTAGTGTCCTCAACTTGGTGCACACACTGATGCCAGGAGCTATAGCACCACTCAGATA
GTTGGTTCCAACATCAAAAGTATTAAGCTTCGGCAGCAAGCTGGAGTCGATAGCAATCTCACTAGA
AAAGCGAGTTGTTCCTCAAGCTGA

CML122

ATGATTGCCTATAAATAGATGAACAGTACCCCCGTACTGTTCACGCTGGATTATAATTGCACTGGT
GTCATTGCCTTCGAACAAGTCAAAGGTATCATTATAATACAATCTTTGAAGATATATTTATGTTCAT
CATGAAATGAATACATGGCATGAAATTACATCTTGTAAGTATATTTTTATATTTATGAGTAATGTA
AACTTGTTATTTATGACCTTTGTCTAAGATTCATTGTACCCAAGATGGAATAATGCTTTGGAGGATG
AAGGTGTTTAATACATAACAAATGTGTTACCTTGCTCTTGATTCACAACAATTAAGAACAAGTGAA
CGACATTGCCGCCCACCTCCGGTGAACTCAGACGACCACCTTCGACAACAAGAACTCGAATAACT
ACATTCGCCATGCCGCCGAAGAAGGCAATAGTGCTAGGGGTTGCCCTTGCACCGCTGGACATCAA
TCAAGAGGGGCTCCCTCTGAGAGAGGTTACCAAGAAGACTGGAGATCTGGATCCCTCGAGTCTAG
AGTCGACCTGCAGGCATGCAAGCTTGGCGTAATCATGGTCATAGCTGTTTCCTGTGTGAAATTGTT
ATCCGCTCACAATTCCACACAACATACGAGCCGGAAGCATAAAGTGTAAAGCCTGGGGTGCCTAA
TGAGTGAGCTAACTCACATTAATTGCGTTGCGCTCACTGCCCGCTTTCCAGTCGGGAAACCTGTCG
TGCCAGCTGCATTAATGAATCGGCCAACGCGCGGGGAGAGGCGGTTTGCGTATTGGGCGCTCTTCC
GCTTCCTCGCTCACTGACTCGCTGCGCTCGGTCGTTCGGCTGCGGCGAGCGGTATCAGCTCACTCA
AAGGCGGTAATACGGTTATCCACAGA

GEMS39

TAGTCAAATATATATGAAAAAAACTTTAGTACAAATATATATATGAAAAAATGGGATGGAGAAAC
TGGTAATTCGAGTTACTGAACTCTTTGTTGTTTTGGAATAGCAGCACCAGGCAAGAGTTGGAGAAC
TAGGAGTCTTCGGTTGTCGCTACGGCCTTCGGATTGCTCCTGTATTCTTCAATGGACAATCCTTGAC
ACGATCACATAAGCACAACAAGAGGAGAAACCCGACCACAGTTCCTAGTCCAAGGGCAACGATG
GCGGCTTTGCTTTTATTGCTGAACAATGGGAGCCCTCTGTGGGTGATCGCTTGCTCGAGCCATTATT
TGAATTCAAGCTCCTCATCTGTGTGAAGCTGACAGGTAGCTTACCGCTGAAAGAATTGTTTGAGAG
GTCAATGTTGAAGCGGTTGTCTAGCTTCCCCAGCCATGGCGGGATATTCCCATCTAACTTGTTACAT
GAAATTTCCAGCACATTGAGGCTTCCCAACCTCTGCAGCCACGGGGGAATTACGCCGATGAGTAG
ACAGTTTGTCAGGATGAGCACCTGCATTGTCTCACCACCGCGGAAATTCCTGGTCAGCACCAAGCT
TGTCAGGTTGGGCAGGTGTTGCAAGACCTGCAATGTCTATGCCAGGTTGTGAAGCTATTCCTGGTC
AGCGAGAGGTACGACACGGATCTCAACTCCTTGAAGCTCTCTGGTATCTCCCCCATGAGCTTGTTC
CTTGTAAGATTCAGTGTCCTCAACTTGGTGCACACACTGATGCTAGAAGCTATAGCGCCACTCAGA
TAGTTGGTTCCAACATCAAAAGTATTAAGCTTCGGCAGCAAGCTGGAGTCGATAGCAATCTCACCA
GAGAGCGAGTTGTTCCTCAAGCTGATTACCCTCAGAAGTGGACAACTTTACAGGGAATCGGGCAA
CTCACCATCCAGCCTATTGGTGGCCAGGTTTACGGACTCTAGCCTCCTCATCTCTCCAAAGACATC
AAGTATGGAGCCTGTGAACTTATTATAGGACAAGTCAAGTTGCATGAT

CIMBL77

TAGTCAAATATATATGAAAAAAACTTTAGTACAAATATATATATGAAAAAATGGGATGGAGAAAC
TGGTAATTCGAGTTACTGAACTCTTTGTTGTTTTGGAATAGCAGCACCAGGCAAGAGTTGGAGAAC
TAGGAGTCTTCGGCTGTCGCTACGGCCTTCGGATTGCTCCTGTATTCTTCAATGGACAATCCTTGAC
ACGGTCACATAAGCACAACAAGAGGAGAACCCCGACCACAGTTCCCAGTCCAAGGGCGACGATG
GCGGATTTGCTTTTATTGCTGAACAATGGGAACCCTCTGTGGGTGACCGCTTGCTCGAGCCATTATT
TGAATTCAAGCTCCTCATCTGTGTGAAGCTGACAGGTAGCTTACCGCTGAAAGAATTGTTTGAGAG
GTCAATGTTGAAGCAGTTGTCTAGCTTCCCTAGCCATGGCGGGATATTCCCATCTAACTTGTTACAT
GAAATTTCCAGCACATTGAGGCTTCCCAAGCTCTGTAGCCACGGGGGAATTACGCCGATGAGCAG
ACAGTTTGTTAGGATGAGCACCTGCATTGTCTCACCGCCGCGGAAATTCCTGGTCAGCACCAAGCT
TGTCAGGTTGGGCAGGTGTTGCAAGACCTGCAATGTCTATGCCAGGTTGTGAAACTATTCCTGGTC
AGCGAGAGGTACGACAGGGATCTCAACTCCTTGAAGCTCTCCGGTATCCCCCCCCACGAGCTTGTT
CCTTGTAAGATTCAGTGTCCTCAACCTGGTGCACACACTGATGCCAGGAGCTATAGCGCCACTCAG
ATAGTTGGTTCCAACATCAAAAGTATTAAGCTTCGGCAGCAAGCTGGAGTCGATAGCAATCTCACC
AGAGAGCGAGTTGTTCCTCAAGCTGATTACCCTCAGCAGTGGAGAACTTTACAGGGAAGCGGGCA
ACTCACCATCCAGCCTATTGGTGGCCAGGTTTACGAACTCTAGCCTCCTCATCTCTCCAAAGACAT
CAGGGATGGAGCCTGTGAACTTGTTATAGGACAAGTCAAGTTGCATGAT

CML170

TAGTCAAATATATATGAAAAAAACTTCAGTACAAATATATATATGAAAAAATGGGATGGAGAAAC
TGGTAATTCGAGTTACTGAACTCTTTGTTGTTTTGGAATAGCAGCACCAGGCAAGAGTTGGAGAAC
TAGGAGTCTTCGGTTGTCGCTACGGCCTTCGGATTGCTCCTGTATTCTTCAATGGACAATCCTTGAC
ACGATCACATAAGCACAACAAGAGGAGAAACCCGACCACAGTTCCTAGTCCAAGGGCAACGATG
GCGGCTTTGCTTTTATTGCTGAACAATGGGAGCCCTCTGTGGGTGATCGCTTGCTCGAGCCATTATT
TGAATTCAAGCTCCTCATCTGTGTGAAGCTGACAGGTAGCTTACCGCTGAAAGAATTGTTTGAGAG
GTCAATGTTGAAGCGGTTGTCTAGCTTCCCCAGCCATGGCGGGATATTCCCATCTAACTTGTTACAT
GAAATTTCCAGCACATTGAGGCTTCCCAACCTCTGCAGCCACGGGGGAATTACGCCGATGAGTAG
ACAGTTTGTCAGGATGAGCACCTGCATTGTCTCACCACCGCGGAAATTCCTGGTCAGCACCAAGCT
TGTCAGGTTGGGCAGGTGTTGCAAGACCTGCAATGTCTATGCCAGGTTGTGAAGCTATTCCTGGTC
AGCGAGAGGTACGACACGGATCTCAACTCCTTGAAGCTCTCTGGTATCTCCCCCATGAGCTTGTTC
CTTGTAAGATTCAGTGTCCTCAACTTGGTGCACACACTGATGCTAGAAGCTATAGCGCCACTCAGA
TAGTTGGTTCCAACATCAAAAGTATTAAGCTTCGGCAGCAAGCTGGAGTCGATAGCAATCTCACCA
GAGAGCGAGTTGTTCCTCAAGCTGATTACCCTCAGAAGTGGACAACTTTACAGGGAATCGGGCAA
CTCACCATCCAGCCTATTGGTGGCCAGGTTTACGGACTCTAGCCTCCTCATCTCTCCAAAGACATC
AAGTATGGAGCCTGTGAACTTATTATAGGACAAGTCAAGTTGCATGAT

GEMS15

TAGTCAAATATATATGAAAAAAACTTTAGTACAAATATATATATGAAAAAATGGGATGGAGAAAC
TGGTAATTCGAGTTACTGAACTCTTTGTTGTTTTGGAATAGCAGCACCAGGCAAGAGTTGGAGAAC
TAGGAGTCTTCGGCTGTCGCTACGGCCTTCGGATTGCTCCTGTATTCTTCAATGGACAATCCTTGAC
ACGGTCACATAAGCACAACAAGAGGAGAACCCCGACCACAGTTCCCAGTCCAAGGGCGACGATG
GCGGATTTGCTTTTATTGCTGAACAATGGGAACCCTCTGTGGGTGACCGCTTGCTCGAGCCATTATT
TGAATTCAAGCTCCTCATCTGTGTGAAGCTGACAGGTAGCTTACCGCTGAAAGAATTGTTTGAGAG
GTCAATGTTGAAGCAGTTGTCTAGCTTCCCTAGCCATGGCGGGATATTCCCATCTAACTTGTTACAT
GAAATTTCCAGCACATTGAGGCTTCCCAAGCTCTGTAGCCACGGGGGAATTACGCCGATGAGCAG
ACAGTTTGTTAGGATGAGCACCTGCATTGTCTCACCGCCGCGGAAATTCCTGGTCAGCACCAAGCT
TGTCAGGTTGGGCAGGTGTTGCAAGACCTGCAATGTCTATGCCAGGTTGTGAAACTATTCCTGGTC
AGCGAGAGGTACGACAGGGATCTCAACTCCTTGAAGCTCTCCGGTATCCCCCCCCACGAGCTTGTT
CCTTGTAAGATTCAGTGTCCTCAACTTGGTGCACACACTGATGCCAGGAGCTATAGCGCCACTCAG
ATAGTTGGTTCCAACATCAAAGGTATTAAGCTTCGGCAGCAAGCTGGAGTCGATAGCAATCTCACT
AGAGAGCGAGTTGTTCCTCAAGCTGATTACCCTCAGCAGTGGAGAACTTTACAGGGAAGCGGGCA
ACTCACCATCCAGCCTATTGGTGGCCAGGTTTACGAACTCTAGCCTCCTCATCTCTCCAAAGACAT
CAGGGATGGAGCCTGTGAACTTGTTATAGGACAAGTCAAGTTGCATGAT

GEMS55

AAGCTCCGCACTTCGCTCTGAAAGCGAACCCTCCAACTAGAGTTGTGCAGTAATGGCAGCATCACG
AGCGATCGTGAGCCTGTGCTTCTCGTCTTCTGCCACCTTTCTAGTGTCTTGAGCCACATAAAACATC
TTCTAGAGTTCTACAAAAGAGGGAATATGAGCAACCCAATACGTAGAACAACCAAGCACAATTCG
GTTCCTAGAAGGGTGTACCGCTAGACACCTCACGCTCCCAAAGGAGCTCCACGTCTAGTTGCTCTA
GGTGAGCATTAGTACTTGATTTTAATTCCCCTAAAGGTATCAAATGTTGGGGGCCTTCCTCTTCCGA
AGGTCCTCAAATAACATTACTAACCATTTGCTTTCAGCATACTACTAAACATTATAGGAGCTTTGTC
ACCAGAGAAGCTTCATAACAGAAGAGAAGTATGACAAAGAAAGCTGCATCAAGCAAGCTTCATCA
TGGTGAAACAGGGAGACAACGTAAGGAGAAGCTGTTGTTCAAGTCATTAAATAGAAATGGCGATG
CTGTACTTGTGACATTTGTAACCCCTATGTGTAACTGTTGAGGGCATGAATGTAATAACGCACGAA
GCTTTGCGACTGTCTATAAATCGATGAACAGTACCCCCGTATTGTTCACGCTGGATTGTAATTGCCC
TCACGTCATTACCTTCAAACAAGCCGAGGGTATCATTGTAATACAAACTTTGAGAATATATTTATA
ATCATTATGAAATGAATACATGGCATGAAATTACATCTTGCGAGTATGTTTTTATACTTGTGGATA
ATGTAAACTTGTTACTTATGACCTTCGTCTAAGATTCATTATACCTAAGAGGGTATAATGCTTCGAA
GGACGAAGGTCTTTAACATATAACAAATGTGTTGCCTTGTTCTTGATTCACATCAGTTGAGAACAA
GTGAACAACATTGGTGCCCACCTCTGGTGAACTCGAACGACCACCTTCGACAACAAGAACTCGAA
CAACTACCTTCGCCATGCCACCGAAGAAGGTTGTAGTGCCAGGGGCTGCCCTTGCACCGCTGGACA
TCAATTAAGAAGGGCTCCCT

Sy999

TAGTCAAATATATATGAAAAAAACTTTAGTACAAATATATATATGAAAAAATGGGATGGAGAAAC
TGGTAATTCGAGTTACTGAACTCTTTGTTGTTTTGGAATAGCAGCACCAGGCAAGAGTTGGAGAAC
TAGGAGTCTTCGGTTGTCGCTACGGCCTTCGGATTGCTCCTGTATTCTTCAATGGACAATCCTTGAC
ACGGTCACATAAGCACAACAAGAGGAGAACCCCGACCACAGTTCCCAGTCCAAGGGCGATGATGG
CGGATTTGCTTTTATTGCTGAACAATGCGAACCCTCTGTGGGTGACCGCTTGCTCGAACCATTATTT
GAATTCAAGCTCCTCATCTGTGTGAAGCTGACAAGTAGCTTACCGCTGAAAGAATTGTTTGAGAGG
TCAATGTTGAAGCAGTTGTCTAGCTTCCCCATCCATGGCGGGATATTCCCATCTAACTTGTTACATG
AAATTTCCAGCACATTGAGGCTTCCCAAGCTCTGCAGCCACGGGGGAATTACGCCGATGAGTAGA

CAGTTTGTCAAGATGAGCACCTGCATTGTCTCACCGCTGCGGAAATTCCTGGTCAGCACCAAGCTT
GTCAGGTTGGGCAGGTGTTGCAAGACCTGCAATGTCTACGCCAGGTTGTGAAACTATTCCTGGTCA
GCGAAAGGTACGACAGGATCTCAACTCCTTGAAGCTCTCCGGTATCCCCCCAACGAGCTTGTTCCT
TGTAAGATTCAGTGTCCTCAACTTGGTGCACACACTGATGCCAGTAGCTATAGCGCCACTCGGATA
GTTGGTTCCAACATCAAAAGTATTAAGCTTCGGCAGCAAGCTGGAGTCGATAGCAATCTCACTAGA
GAGCGAGTTGTTCCTCAAGGTGATTACCCTCAGCAGTGGAGAACTTTACAGGGAAGCGGGCAACT
CACCATCCAGCCTATTGGTGGCCAGGTTTACGGACTCTAGCCTCCTCATCTCTCCAAAGACATCAG
GGATGGAGCCTGTGAACTTGTTATAGGACAAGTCAAGTTGCATGAT

CIMBL71

TAGTCAAATATATATGAAAAAAAACTTTAGTACAAATATATATATGAAAAAATGGGATGGAGAAA
CTGGTAATTCGAGTTACTGAACTCTTTGTTGTTTTGGAATAGCAGCATCAGGCAAGAGTTGGAGAA
CTAGGAGTCTTCGGCTGTCGCTACGGCCTTCGGATTGCTCCTGTATTCTTCAATGGACAATCCTTGA
CACGGTCACATAAGCACAACAAGAGGAGAACCCCGACCACAGTTCCCAGTCCAAGGGCGATGATG
GCGGATTTGCTTTTATTGCTGAACAATGCGAACCCTCTGTGGGTGACCGCTTGCTCGAGCCATTATT
TGAATTCAAGCTCCTCATCTGTGTGAAGCTGACAAGTAGCTTACCGCTGAAAGAATTGTTTGAGAG
GTCAATGTTGAAGCAGTTGTCTAGCTTCCCCATCCATGGCGGGATATTCCCATCTAACTTGTTACAT
GAAATTTCCAGCACATTGAGGCTTCCCAAGCTCTGCAGCCACGGGGGAATTACGCCGATGAGTAG
ACAGTTTGTCAAGATGAGCACCTGCATTGTCTCACCGCTGCGGAAATTCCTGGTCAGCACCAAGCT
TGTCAGGTTGGGCAGGTGTTGCAAGACCTGCAATGTCTATGCCAGGTTGTGAAACTATTCCTGGTC
AGCGAAAGGTACGACAGGATCTCAACTCCTTGAAGCTCTCCGGTATCCCCCCAACGAGCTTGTTCC
TTGTAAGATTCAGTGTCCTCAACTTGGTGCACACACTGATGCCAGTAGCTATAGCGCCACTCGGAT
AGTTGGTTCCAACATCAAAAGTATTAAGCTTCGGCAGCAAGCTGGAGTCGATAGCAATCTCACTAG
AGAGCGAGTTGTTCCTCAAGGTGATTACCCTCAGCAGTGGAGAACTTTACAGGGAAGCGGGCAAC
TCACCATCCAGCCTATTGGTGGCCAGGTTTACGGACTCTAGCCTCCTCATCTCTCCAAAGACATCA
GGGATGGAGCCTGTGAACTTGTTATAGGACAAGTCAAGTTGCATGAT

CIMBL127

TAGTCAAATATATATGAAAAAAAAACTTTAGTACAAATATATATATATATGAAAAAATGGGATGG
AGAAACTGGTAATTCGAGTTACTGAACTCTTTGTTGTTTTGGAATAGCAGCACCAGGCAAGAGTTG
GAGATCTAGGAGTCTTCGGTTGTCGCTACGGCCTTCGGATTGCTCCTGTATTCTTCAGTGGACAATC
CTTGACACGGTCACATAAGCACAACAAGAGGAGAACCCTGACCACAGTTCCCAGTCCAAGGGCGA
CGATGGCGGATTTGCTTTTATTGCTGAACAATGGGAACCCTCTGTGGGTGACCGCTTGCTCGAGCC
ATTATTTGAATTCAAGCTCCTCATCTGTGTGAAGCTAACAGGTAGCTTACCGCTGAAAGAATTGTT
TGAGAGATCAATGTTGAAGCAGTTGTCTAGCTTCCCCAGCCATGGCAGGATATTCCCATCTAACTT
GTTACATGAAATTTCCAGCACATTGAGGCTTCCCAAGCTCTGCAGCCACGGGGGAATTACGCCGAT
GAGTAGATAGTTTGTCAGGATGAGCACCTGCATTGTCTCACCGCCGCGGAAATTCCTGGTCAGCAC
CAAGCTTGTCAGGTTGGGCAGGTGTTGCAAGACCTGCAATGTCTATGCCAGGTTGTGAAACTATTC
CTGGTCAGCGAGAGGTACGGCAGGGATCTCAACTCCTTGAAGCTCTCCGGTATCCCCCACGAGCTT
GTTCCTTGTAAGATTTAGTGTCCTCAACTTGGTGCACACACTGATGCCAGGAGCTATAGCACCACT
CAGATAGTTGGTTCCAACATCAAAAGTATTAAGCTTCGGCAGCAAGCTGGAGTCGATAGCAATCTC
ACTAGAAGAGCGAGTTGTTCCTCAAGCTGATTACCCTCAGCAGTGGAGAACTTTACAGGGAAGCG
GGCAACTCACCATCCAGCCTATTGGTGGCCAGGTTTACGGACTCTAGCCTCCTCATCTCTCCAAAG
ACATCAGGGATGGAGCCTGTGAACTTGTTATAGGACAAGTCAAGTTGCATGAT

Table S6  Primers for the 10 candidate genes assessed by reverse transcription and fluorescence quantitative PCR

| primer name | sequence |
|---|---|
| GRMZM2G352959-F | TACCACCCTTGCATCTGTGA |
| GRMZM2G352959-R | GAACCCGTGCTTTCTTCTCC |
| GRMZM2G003165-F | GCACGTTCTCGTCGACTACT |
| GRMZM2G003165-R | GATGTTCACGTACCCCGACA |
| GRMZM2G419655-F | AACAACTCGCTCTCTGGTGA |
| GRMZM2G419655-R | CAGTGTCCTCAACTTGGTGC |
| GRMZM2G419660-F | CAGTGTCCTCAACTTGGTGC |
| GRMZM2G419660-R | AACAACTCGCTCTCTGGTGA |
| GRMZM2G542753-F | GGCTCAAGTATGCACTGACG |
| GRMZM2G542753-R | ACGGAAGCAGCCTGTAGTTC |
| GRMZM2G014180-F | CCCATCACCAGGCTCATCC |
| GRMZM2G014180-R | AGACCACTTGTTCTCGGCAT |
| GRMZM2G369956-F | ACCGATATTTTGTTGGCGCA |
| GRMZM2G369956-R | CCCTATCCAACTGCCCAGAT |
| GRMZM2G134597-F | GCTCTCGTCCTTCCTCTCTC |
| GRMZM2G134597-R | GGAGCCTCACCCAAAACAAG |
| GRMZM2G167673-F | ATGGTGGAGCTGATGGTGG |
| GRMZM2G167673-R | ATGACGTCGAAGGAGAAGCT |
| GRMZM2G511067-F | AACGCGAGAAGGCATGTTG |
| GRMZM2G511067-R | TGACACGGGTAATTATAAGGCA |
